# Public–Private engagement and health systems resilience in times of health worker strikes: a Ghanaian case study

**DOI:** 10.1093/heapol/czae018

**Published:** 2024-03-18

**Authors:** Bettina Buabeng-Baidoo, Jill Olivier

**Affiliations:** School of Public Health, University of Cape Town, Rondebosch 7701, South Africa; School of Public Health, University of Cape Town, Rondebosch 7701, South Africa

**Keywords:** Private providers, private sector, public/private, health workers, non-governmental institutions, human resources

## Abstract

In low and middle-income countries like Ghana, private providers, particularly the grouping of faith-based non-profit health providers networked by the Christian Health Association of Ghana (CHAG), play a crucial role in maintaining service continuity during health worker strikes. Poor engagement with the private sector during such strikes could compromise care quality and impose financial hardships on populations, especially the impoverished. This study delves into the engagement between CHAG and the Government of Ghana (GoG) during health worker strikes from 2010 to 2016, employing a qualitative descriptive and exploratory case study approach. By analysing evidence from peer-reviewed literature, media archives, grey literature and interview transcripts from a related study using a qualitative thematic analysis approach, this study identifies health worker strikes as a persistent chronic stressor in Ghana. Findings highlight some system-level interactions between CHAG and GoG, fostering adaptive and absorptive resilience strategies, influenced by CHAG’s non-striking ethos, unique secondment policy between the two actors and the presence of a National Health Insurance System. However, limited support from the government to CHAG member facilities during strikes and systemic challenges with the National Health Insurance System pose threats to CHAG’s ability to provide quality, affordable care. This study underscores private providers’ pivotal role in enhancing health system resilience during strikes in Ghana, advocating for proactive governmental partnerships with private providers and joint efforts to address human-resource-related challenges ahead of strikes. It also recommends further research to devise and evaluate effective strategies for nations to respond to strikes, ensuring preparedness and sustained quality healthcare delivery during such crises.

Key messages

- The collaboration between Christian Health Association of Ghana (CHAG) and Government of Ghana (GoG) highlights the crucial role of faith-based health providers (FBHPs) in enhancing system-level resilience during strikes, indicating a pathway for similar engagements in other low-middle-income countries.
- The GoG should bolster support for private facilities during strikes and reinforce health insurance schemes to ensure timely supplier payments, facilitating patient care in the private sector amid public service disruptions.
- Governments should shift from merely averting health worker strikes to crafting robust preparedness and response policies at multiple levels, addressing both financial resources and nurturing trust with professional unions.
- Proactive engagement with professional bodies and stakeholders (including partnerships with the private sector) to address human resources for health (HRH)-related challenges can contribute to averting strikes and improving overall HRH management.

## Introduction

There is widespread acknowledgement of the role of partnerships between state and non-state actors in strengthening health systems and achieving Universal Health Coverage (Clarke *et al*., 2019; World Health Organization, 2020). In the epoch of the COVID-19 pandemic, health systems resilience has gained attention (Saulnier *et al*., 2021), and the role of partnerships in building resilience amid shocks and stressors highlighted (Casady and Baxter, 2020). In low and middle-income countries (LMICs), where healthcare demand exceeds supply, public resources are often insufficient to satisfy health system goals. In such settings, the private health sector^1^, as part of a plural system, may improve access to health services, provided the right partnership conditions are created (International Finance Corporation, 2011).

The model of public–private engagement (PPE) influences how well private providers might complement the public system (Ravishankar *et al*., 2016). PPE can be defined as ‘the mechanism of engaging with the non-state sector in line with state priorities through a temporary or partial transfer of assets or by sharing the responsibility for service provision between the public and the private sector’ (Whyle and Olivier, 2017). Faith-based health providers (FBHPs)^2^ are a prominent grouping of private usually non-profit providers that have been in existence for decades in Africa (Dimmock *et al*., 2012), dominated by Christian providers who are usually networked under umbrella bodies or ‘Christian Health Associations’ (CHAs) (Olivier *et al*., 2015). Whyle and Olivier (2017) propose that these providers have a unique PPE model with the state, based on years of collaboration—which may mean FBHPs have unique potential for contributing to overall systems resilience.

A consortium of health system experts identified the need for additional research on the partnerships with the private sector (with specific mention of FBHPs) as one of five important evidence gaps on systems resilience (Saulnier *et al*., 2021). Though definitions vary, resilience typically refers to a system’s capacity to absorb, respond to and grow stronger from shocks (Kruk *et al*., 2015). The concept has been applied to diverse health system shocks, such as epidemics, political and economic crises (Barasa *et al*., 2017). The phrase ‘everyday resilience’ characterizes the resilience capacities and strategies required to respond to chronic system stressors (Gilson *et al*., 2017; Kagwanja *et al*., 2020).

In African systems, health worker^3^ strikes (HWS) are both a stressor and a shock—whose impact depends on factors such as duration, extent (reduced hours or complete work stoppage), type of health worker on strike, actions of striking workers, health-seeking behaviours affected and whether there is alternative access to care (Kaguthi *et al*., 2020; Waithaka *et al*., 2020). Strike action is experienced globally but the effects are particularly severe in LMICs due to pre-existing health system fragilities (Russo *et al*., 2019; Waithaka *et al*., 2020). Though there is conflicting evidence on the direct effects of HWS on population mortality (Cunningham *et al*., 2008; Metcalfe *et al*., 2015), strikes in LMICs are thought to have significant health systems effects, for example leading to a decrease in in/out-patient flow, a breakdown in trust between community and health workers, decreasing health worker motivation or entrenching inequalities as strike effects are felt disproportionately by the poor (Waithaka *et al*., 2020; Scanlon *et al*., 2021a; 2021b; Adam *et al*., 2018). HWS are concerning as human resources for health (HRH) are arguably the most fundamental building block of a health system (World Health Organization, 2016). LMICs demonstrate systemic weaknesses in HRH, including chronic shortages and inequitable distribution of workers, are important context for understanding HWS (Chen *et al*., 2004). Stated causes of HWS include inadequate conditions of service, poor working environment, dissatisfaction with leadership and management and the refusal of government to comply with bargaining agreements (Russo *et al*., 2019).

Few studies have explored HWS and systemic responses. Most literature focuses on the impact of HWS on mortality and the ethical nature of strikes (Chima *et al*., 2013). Studies of HWS in LMICs found that alternative care options are vital for population health—but shifting care to private facilities might also have serious consequences, such as catastrophic costs for the poor or the compromise of continuity or quality of care (Scanlon *et al*., 2021a; 2021b; Adam *et al*., 2018). There is currently no study of the role that private providers may play in offering health system resilience during public sector strikes in LMICs or the partnership arrangements involved therein.

Ghana presents a useful context for study as it has almost endemic public-sector HWS in response to persistent HRH challenges (Agyepong *et al*., 2012; Seniwoliba, 2013; Asamani *et al*., 2021). Though strides have been made towards responding to HRH challenges, tensions persist and strikes occur routinely with significant systemic impacts (Ampofo *et al*., 2022). It has been suggested that the private sector in Ghana, especially the grouping of FBHPs networked by the Christian Health Association of Ghana (CHAG), might play an important role, as the Government of Ghana (GoG) has been cited to publicly direct patients to them during HWS (Yeboah and Buckle, 2017). This study explored the case of the interaction between CHAG and the GoG during HWS to answer the question: ‘Does public-private engagement provide health systems resilience during public sector HWS in Ghana?’

### Study context: partnership during strikes in Ghana

Ghana has a pluralistic health system consisting of public and private providers. The public sector comprises two institutions: the Ministry of Health (MoH), which oversees policy formulation and resource mobilization, and the Ghana Health Service (GHS), an independent administrative entity responsible for implementing national policies (Ghana Ministry of Health, 2020). The GHS provides primary and secondary services, and private and quasi-governmental institutions^4^ supplement these services (Asamani *et al*., 2021). Thus, the MoH regulates and interacts with the private sector. The private sector, comprising varied for- and non-profit providers, supplies an estimated 50–60% of all health services in Ghana (Bitran, 2011; Amo-Adjei, 2016). Estimates on healthcare service distribution between for-profit and not-for-profit providers vary (Olivier *et al*., 2014; Grieve and Olivier, 2018). In 2011, administrative data indicated that FBHPs contributed 30–40% of healthcare provision, contrasting with household survey data which suggested less than 10% (Olivier *et al*., 2014).

Table 1 shows key policy reforms in Ghana impacting PPE context and health worker strikes, with the 2003-introduced National Health Insurance Scheme (NHIS) notably shaping public–private interactions (Bitran, 2011). By 2013, the scheme covered 60% of the population and offered a benefits package covering nearly 95% of the disease burden (Addae-Korankye, 2013). Public and most private providers are part of the NHIS, allowing cardholders to access care at approved facilities (Ghana Ministry of Health, 2020). While credited for bolstering healthcare utilization and reducing patients’ financial strain, the NHIS faces criticisms over issues like fund mismanagement and payment delays, affecting both public and private providers (Bitran, 2011; Addae-Korankye, 2013).

**Table 1.** Policy reforms influencing PPE and health workers strikes prior to and during 2010–2016

Policy	Year	Description	Outcome	Additional notes
**Additional Duty Hours Allowance** (ADHA) Policy (Agyepong *et al*., 2012)	1998	Initiated to address wage disparities by compensating extra hours beyond the standard work week, targeting retention and recruitment of younger doctors amidst salary hikes in military hospitals.	Initially applicable to doctors, but extended to other health staff following industrial actions. Phased out in 2005 due to financial burden on the economy.	Policy led to a cycle of strikes arising from payment delays, impacting healthcare service delivery adversely. Transitioning ADHA into regular salaries in 2005 spawned further industrial actions.
**Private Health Sector Policy Plan** (Bitran, 2011; Ghana Ministry of Health, 2012)	2003	Launched to accelerate the growth of the private healthcare sector and nurture public–private partnerships, augmenting healthcare accessibility and efficiency.	Achieved better integration of private sector into the national healthcare system, promoting a collaborative approach to healthcare delivery.	Policy marked a significant step towards leveraging private sector resources and expertise in addressing healthcare challenges in Ghana.
**National Health Insurance Act** (Act 2003, Act 650) (Bitran, 2011; Gobah and Liang, 2011; Nguyen *et al*., 2011; Addae-Korankye, 2013; Ghana Ministry of Health, 2020)	2003	Instituted to establish the NHIS covering a broad spectrum of healthcare services to reduce out-of-pocket expenses for individuals.	Facilitated access to healthcare services at private facilities, especially during health worker strikes, enhancing healthcare accessibility.	Significantly improved healthcare accessibility, although challenges like funding and operational efficiency remain.
**Single Spine Salary Structure** (SSSS) (Aliu and Fuseini, 2014; Seniwoliba, 2014; Asare and Mpere, 2017)	2010	A pivotal element of a broader public sector reform (Single Spine Pay Policy) aimed at achieving a fair and coherent salary determination system based on job classification, responsibility levels, among other factors.	Encountered initial resistance from health workers with a notable strike in 2011 led by the GMA over grading distortions.	SSSS was crucial in streamlining salary structures, but its implementation faced hurdles including dissatisfaction over the migration process and perceived grading anomalies.
**Private Health Sector Development Policy** (Ghana Ministry of Health, 2012)	2012	Addressed the shortcomings of the 2003 policy by formalizing more partnership agreements to foster cooperation between public and private healthcare providers.	Broadened the scope of engagement with private providers through MOUs enhancing the public–private collaborative framework.	The policy facilitated a more structured engagement with private providers, fostering a conducive environment for collaborative initiatives in healthcare delivery.

ADHA, Additional Duty Hours Allowance Policy; NHIS, National Health Insurance; SSSS, Single Spine Salary Structure; GMA, Ghana Medical Association; MOU, Memorandum of Understanding.

While there may be other institutions not included in this table, our focus predominantly lies on policy reforms in Ghana that are central to understanding our analysis and results.

FBHPs in Ghana are predominantly networked under the 1967-founded CHAG, with members being the facilities who are also linked to various denominational bodies (CHAG Member Institutions, or CMIs) (Grieve and Olivier, 2018). In the 1960s, there were approximately 21 CMIs; this number has grown to about 300 (Yeboah and Buckle, 2017). CHAG’s National Secretariat is the coordinating body for CMIs and is the entity which communicates directly with the MoH—this unique PPE relationship, in place for half a century (Whyle and Olivier, 2017), is built around an agreement on HRH. Following the foundational Adibo Report’s^5^ recommendations in the 1970s, the GoG began subsidizing health professional’s salaries in CMIs, and currently covers about 92% of these wages (Yeboah and Buckle, 2017). Their cooperation was formalized in a 2003 MOU, with subsequent amendments in 2006 (Box 1) (Dimmock *et al*., 2017). Today, CHAG is informally seen as an agency within the MoH with its own personnel similar (though to a lesser extent) to the GHS (Yeboah and Buckle, 2017; Grieve and Olivier, 2018).

Box 1. Key features of CHAG–MOH memorandum of understanding (source Ghana–MOH and, 2006)

**HRH management**

Responsibilities of CHAG

- Support human resource requirements of MOH.
- Agree to MOH staffing norms.

Responsibilities of MOH

- Provide support and access to training facilities for CMIs.
- Facilitate equitable distribution of health professionals among non-government providers.
- Will supplement financial requirements of CHAG and CMIs based on approved budgets.

**Finance**: CHAG will submit annual plans and budgets to MOH for financial assistance.

**Information sharing**: CHAG will be involved in the development of national health policies and programmes.

**Conflict resolution**: Agreement to settle disputes outside of court.

**Duration and termination**: MOU will be operational from December 2003. Can be terminated by either party by giving notice in writing 3 months prior.

Reports of HWS can be seen in Ghana since independence. An important part of this history was the unionization of public sector workers (Table 2). The 1992 Constitution of Ghana enshrines the unionization of workers as a right; but the 2003 Labor Act restricts most ‘essential’ health workers from striking—ordering disputes to be negotiated for 3 days, then sent to the National Labour Commission (NLC) for compulsory arbitration (Ghana, 2003). However, in reality, many parties bypass this process, resulting in persistent strikes despite the policy constraints (Seniwoliba, 2014).

**Table 2.** Important institutions and professional unions in the context of health worker strikes in Ghana

Institution	Year established	Description	Relevance to health worker strikes
Ghana Medical Association (GMA) (Ghana Medical Association, 2021)	1957	Represents over 7000 medical and dental practitioners in Ghana.	Often at the forefront of negotiations during health worker strikes, advocating for better pay and working conditions for medical practitioners.
Ghana Registered Nurses and Midwives Association (GRNMA, 2023)	1960	The largest professional health body in Ghana representing professional nurses and midwives.	Represents nurses and midwives during strikes and negotiations, seeking better terms of employment and working conditions.
Government Hospital and Pharmacist Association (GHOSPA)		Represents professional pharmacists working in government hospitals across Ghana.	Engages in industrial actions and negotiations for better remuneration and working conditions for pharmacists.
Fair Wages and Salary Commission (FWSC) (Ghana Ministry of Employment and Labour Relations, 2022)	1996	Independent commission promoting fair and equitable wages and salaries in the public sector under Fair Wages and Salaries Act 525.	Plays a crucial role in negotiations during strikes, addressing wage disparities and working to resolve salary-related grievances.
National Labour Commission (NLC) (Ghana Ministry of Employment and Labour Relations, 2022)	2003	Established under Part XVIII of the Labour Act, 2003 (Act 651). Mediates industrial relations and disputes between employers and employees.	Mediates during health worker strikes, striving to find amicable resolutions to disputes and ensuring the enforcement of labour laws.

While there may be other institutions not included in this table, our focus predominantly lies on the institutions that are central to understanding our analysis and results.

The context of strikes is also influenced by Ghana’s complex HRH system. While technically decentralized, the MoH still controls most HRH functions (Sumah and Baatiema, 2019). Health worker salaries, market premiums and pension contributions are decided by the engagement of various public sector policies and institutions such as the Fair Wages and Salary Commission, the MoH and the Ministry of Finance which are often the target of HWS (Asare and Mpere, 2017).

## Methods

This study was a flexible, primarily qualitative, descriptive and exploratory single case study (with three embedded cases). This approach was appropriate for an in-depth enquiry about an unusual phenomenon in context and requiring combination of varied evidence sources (Yin, 2009). The case was defined as ‘the engagement^6^ between CHAG and the GoG during HWS’ (**Box 2**) in the period of 2010–2016 (outlined below), selected due to the high frequency of HWS in this period. We had the following objectives: (1) to describe the context of public sector HWS in Ghana (2); to describe the role of CHAG in responding to public sector HWS; (3) to explore the factors influencing the engagement between CHAG and the GoG during HWS; and (4) to explore how the engagement between CHAG and GoG may have contributed to health systems’ resilience during HWS.

This was a sub-study of a larger project (see acknowledgements), which set out to investigate the historical relationship between the public sector of Ghana and non-state non-profit organizations. The initial findings indicated that the contribution of CHAG to health systems resilience during HWS was a recurring and underexplored theme.

**Figure 1.**
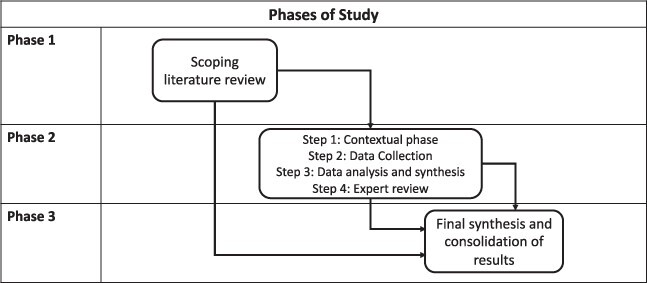
Phases of study

This study was conducted in three phases (Figure 1): (1) a scoping review, (2) a single case study of CHAG-GoG interaction, and (3) results analysis and synthesis. The scoping literature review investigated the background of HWS, the public–private mix and health systems’ resilience literature related to Ghana and LMICs. The analysis revealed adequate publicly accessible but poorly collated data to conduct the case. The scoping review (**see Suppl 1**) sourced evidence, operationalized key terms and identified conceptual frames for case analysis, including the Russo *et al*., (2019) conceptual framework for understanding health sector strikes in low-income countries, the CHA-State PPE model as described by Whyle and Olivier (2017) and the Kagwanja et al.’s (2018) health system resilience framework.

As part of the case study, we historically contextualized the public–private mix and the background of HWS in Ghana. In the scoping review process, we found limited academic peer-reviewed literature of direct relevance from traditional sources, so broadened the search to repositories of grey materials such as institutional databases and media archives (**Box 3**). We followed a qualitative document analysis approach—which recognizes the importance of understanding the perspective, scope and reliability of documents (Bowen, 2009). Though the unique nature of this study necessitated extracting information from a wide range of documents, 229 documents were found to be particularly relevant to the context of HWS in Ghana from 2010 to 2016 (see **Suppl 2**). Of these, 56 were specifically relevant to the engagement between the CHAG and the GoG during HWS. From this phase, the embedded case selection of 3 (of 10) strike incidents were purposively selected based on the inclusion criteria of: (1) strike incidents that were of relevance to the case description, and (2) availability of sufficient ‘reliable’ evidence. Our analysis focused on these selected embedded units but was not exclusive to these strike incidents.

The case was built through the iterative collection and analysis of varied data types. In addition, we conducted secondary analyses of interview transcripts from the primary study. The primary study involved one-on-one semi-structured interviews with senior managers and policy makers working in the MoH and CHAG. A rapid appraisal process was followed to extract transcripts with relevant information relating to strikes from the broader study—and 12 key informant transcripts were selected for review and secondary analysis (responses were anonymized). Collected data were systematically categorized and thematically analysed according to the research objectives. Themes were identified deductively, against the analytical framework developed earlier, and inductively, by examining emerging patterns. Data collection from multiple sources facilitated the triangulation of evidence to enhance rigour. In addition, results and analysis were reviewed by two experts who initially participated in the broader study and are currently involved in CHAG and MoH engagement. Importantly, the expert verification process occurred after data collection and analysis, and no interviews were conducted as part of the data collection phase of this sub-study.

Box 2. Single embedded case study clarification

**Context**: Ghana 2010–2016.

**The Case**: ‘engagement between CHAG and GoG during health worker strikes’.

**Embedded cases**: Individual strike incidents.

## Results

Reporting on case analysis is always challenging, given the level of contextual detail involved. In this section, we present summaries of key results—and provide fuller descriptions in the Supplementary materials, as well as more detailed evidencing of claims. Tables here highlight sources contributing to major themes, with expanded tables in the supplementary data. Supplementary 2 lists all sources and their unique identifiers (Box 4) used in our analysis. The case description stems from three embedded cases selected from 10 options during this period.

### Case description: the engagement between CHAG and GoG during strikes 2010–2016

**Figure 2.**
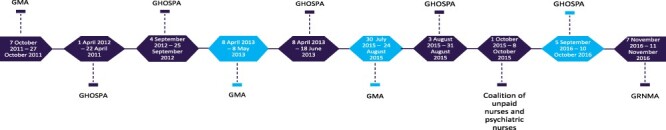
Timeline health worker strikes in Ghana 2010–2016 (source: author)

From 1999 into the 2000s, Ghana experienced a major series of HWS, with 10 separate strike incidents identified between 2010 and 2016, the majority lasting between 2 and 3 weeks (Figure 2). To address the concerns about wage disparities, the GoG introduced the Single Spine Salary Structure (SSSS) (Table 1) in 2010, centralizing salaries for public-sector workers. Challenges with the SSSS (including payment delays and salary dissatisfaction) and the absence of a standardized service conditions persisted, which fuelled strikes between 2010 and 2016. In 2013, the SSSS implementation led to conflict between the GoG and the GMA (Table 3). Doctors protested a reduction in their basic salaries, leading to a 3-week strike. The standoff ended when the NLC mandated a salary adjustment. In 2015, the GMA (see Table 2) went on strike again for 3 weeks over service conditions. It concluded after intervention by civil society groups and an NLC directive for compulsory arbitration. In 2016, Government Hospital and Pharmacist Association (GHOSPA) protested against perceived wage and service disparities stemming from the SSSS again—and was called off 1 month later after an investigative committee’s recommendations. Most strike incidents followed a progressive pattern: outpatient services were initially withdrawn, followed by in-patient services and emergency services in 6 of the 10 strike incidents. In the 2015 doctor strike, there were threats of a mass resignation of doctors if their complaints were not addressed, although this never occurred (MB31; MB45).

Figure 2 represents strikes organized by professional worker unions or coalition of workers in Ghana between 2010 and 2016. This timeline excludes the persistent threats of strikes by professional unions throughout this time, as well as any regional or hospital-specific strikes. For simplicity, we also excluded strikes that were not primarily related to health workers or strike incidents by unemployed health workers. The start and end date were decided based on official communication from the professional union (indicating when they were officially on strike and when the strike ended). The colour code blue represents the three embedded strike incidents selected for further analysis. Purple represents other strike incidents that occurred in this period but were not selected as embedded units. Abbreviation: GMA, Ghana Medical Association; GHOSPA, Government Hospital and Pharmacist Association; GRNMA, Ghana Registered Nurses and Midwives Association.

**Table 3.** Characteristics of embedded strike incidents

Strike incident	Striking body	Onset characteristics	Macro socio-economic and political factors	Resolution	Impact
8 April–8 May 2013Code: A	GMA	Doctors striking for payment of their market arrears which were outstanding since being migrated onto the SSSS. On 27 March 2013, the NLC adopted a payment schedule drawn up by the FWSC in which payment of outstanding arrears would occur in three instalments. This payment plan was rejected by the GMA. GMA also striking against reduction in their pension after migration onto the SSSS. MA1, MA4, MA8, MA12, MA12, J5, J11, J8, J9, J11(212), J12.	NDC President Mahama won December 2012 elections with a narrow margin. Several public sector strikes occurring in the nation-primary, secondary teachers and university lecturers on strike. Energy crisis, economic slowdown and budget deficit. MA4.	Strike called off after a ruling by the NLC that the gov should restore the conversion difference being sought after by the striking doctors. [MA3, MA7, J9, J11(212)].	Out-patient services were withdrawn by doctors from 8–14 April. Doctors threatened that after the 22 April they would suspend all emergency services in hospitals. Disproportionate harm on vulnerable patient groups, i.e. diabetic patients. Crippling public sector as strike occurred congruent with other strikes in the primary and tertiary sector. MA1, MA7, MA8, MA12, MA34.
30 July 2024 August 2015Code: B	GMA	GMA demanding gov finalize their conditions of service; an issue which had been outstanding including how additional doctors work each month was calculated and reimbursed. According to the GMA, they received no response from government after seven months. MB1, MB4, MB5, MB9, MB14, MB20, MB22, MB28, MB30, MB1, MB9, MB14, MB16, MB30, J8, P5, C1.	Political tension rising as 2016 due to contentious elections. Slow economic growth in Ghana characterized by rising debt and a ballooning public wage bill. IMF loan condition for gov to reduce spending on public wages. MB1, MB4, MB5, MB9, MB14, MB20, MB22, MB28, MB30 MB17, MB22, MB24, MB47, MB54 J1, J2.	GMA called off strike after calls from multiple groups for the doctors to return to hospitals. Executive meeting held by GMA where decision to end strike was taken; but decision not welcomed by all in the GMA. Directive issued by NLC for GMA to engage in compulsory arbitration. MB1, MB2, MB6, MB7, MB35, MB41, MB3, MB8, P5.	In the first week of the strike, outpatient services were withdrawn with inpatient and emergency services continuing. In the second week, emergency services were. Reports that 500 patients had died since onset of strike. MB17, MB8, MB9, MB14, MB19, MB21, MB28, MB31, MB42, MB45, MB61. Tension and aggravating language between GMA and government communicators. MB3, MB6, MB7, MB10, MB15, MB20, MB28, MB30, MB31, MB34, MB38, MB59.
5 September– 10 October 2016Code: C	GHOSPA	GHOSPA demanding that their salary grade structure, interim market premium and conditions of service should be reviewed by the FWSC. MC1, MC2, MC3, MC6, T1, MC3, MC4, MC5, MC6, MC9, MC10, MC11, MC12, MC13, MC14, J8.	Rising political tension over National elections to be held in October. Continued slow economic growth. MC12, MC14, J8.	Appeals from civil society for an end to the strikes. At a National Executive meeting of GHOSPA on 10 October where members agreed to end strike after mounting public pressure. MOH instituted a committee to investigate strike. MC12, MC14, J8.	Shutdown of gov pharmacies. Only inpatients attended to. MC1, MC5, MC7, MC11, MC14, MC6, MC7.

This table is a summary of the sources that contributed to the main themes we identified in the description of characteristics of health worker strikes in the three embedded cases. The main themes were determined based on the Russo et al.’s framework for describing strike incidents in low-income countries. **Supplementary 3** shows an expanded table with all the sources that contributed to themes across all 10 strike incidents we identified.

Unique identification codes were assigned to sources (**see Suppl 2**): the first letter denoting type of source (**Box 4**), the second letter (if applicable) to a specific strike incident and numbers in brackets (if present) indicate specific page numbers.

NDC, National Democratic Congress; Doc, Doctor; GMA, Ghana Medical Association; GHOSPA, Government Hospital Pharmacists Association; GNRMA, Ghana Registered Nurses and Midwives Association; QHI, Quasi-governmental health institutions; Gov, Government; SSSS, Single Spine Salary Structure.

There is limited quantitative data on the effects of HWS on health system outcomes, although some sources indicate the impact was severe and disproportionately detrimental to the poor and vulnerable (MA7; MA22; MB14; MB19; MB34). It was estimated that 500 deaths occurred during the first 17 days of the 2015 GMA strike to strike-related issues, with potential undercounting in rural areas (MB17, MB14). It was reported that during pharmacist strikes, services for maternal patients and patients with diabetes/HIV&AIDS were withdrawn (MC11, MC13, MC14, ME8, ME9). The strikes were also perceived to lead to significant financial losses for the health system, especially the pharmacist strikes, as sales from pharmaceutical drugs are a major revenue source. Strikes also reportedly increased tensions and led to a breakdown of trust particularly between hospital administrators and striking workers [MF2, MF3, J5(2)].

Box 3. Data sources

**Institutional databases sources**

- Ghana Medical Association
- Fair Wages and Salary Commission
- Ghana National Labor Commission
- Ghana Ministry of Health
- Christian Health Association of Ghana
- Ministry of Labor

**Media archives sources**

- Ghanaweb
- Myjoyonline
- AllAfrica
- Citinews

**Peer-reviewed literature sources**

- Google Scholar
- PubMed
- Ebscohost

Box 4. Data unique identifiers

M#: Media Report

J#: Peer-reviewed Journal Article

P#: Policy document

C#: Communication

G#: Government reports

N#: Non-government reports

B#: Books

I#: Secondary Interview Transcripts

T#: Theses

Notably, almost all strike incidents occurred after a breakdown in negotiations between professional groups and governmental bodies, where workers felt their interests were not taken seriously by government. An important theme was the aggravating role of language. The occasionally violent and disrespectful language used by government officials (especially government communicators) and union leaders heightened tensions and was perceived to prolong strikes (MB3, MB6, MB7). For example, during the 2015 strikes, it was reported that ‘a senior member of the Ghana Medical Association has slammed former Chief of Defense…for asking doctors to go to China and play football if they want money’ (MB6).

Interrelated macro-contextual factors, particularly political tensions, influenced the severity of strikes. During 2012 and 2016, two contentious elections occurred, with the political landscape dominated by the polarized National Democratic Congress (NDC) and the National Patriotic Party (NPP). The passing of President John Atta Mills in 2012 saw President John Mahama’s succession. The NPP heavily criticized the NDC’s handling of the doctor strikes (MB24, MB27, MC12), leading to accusations and the 2015 and 2016 strikes becoming a political battleground.

From 2010 to 2016, Ghana grappled with economic challenges, including an energy crisis, stagnated growth and increasing debt. A soaring public sector wage bill, exacerbated in part by the SSSS, resulted in public sector salaries consuming 70% of government revenues in 2012 (MB17, MB54). In 2015, the International Monetary Fund’s $918 million loan to Ghana came with wage reduction conditions, placing added fiscal pressure on the GoG influencing the duration and intensity of subsequent strikes (MB54, MB22).

### CHAG and GoG interaction during health worker strikes 2010–2016

Tables 4 and 5 show the sources that contributed to the key themes we describe below.

**Table 4.** Summary of important sources engagement between CHAG and GoG during strikes

Code and source	Type	Title	Strike incident	Findings
MB33 (KasapaFm, 2015)	Media Report (GNA)	Catholic Church adopting slave labor in Ghana—ex-Minister	2015	Ex minister of health challenges National Catholic Health Service dismissal of 14 employees for joining the national doctor strike.
J14 (Yeboah and Buckle, 2017)	Journal paper	The evolving partnership between the GoG and national faith-based health providers: leadership perspective and experiences from the Christian Health Association of Ghana	General	(1) CHAG has a strict non-striking position, and this position allows for the delivery of 24-hour health services during health worker strikes in Ghana, helping to mitigate impact on vulnerable populations. (2) Tensions between CHAG and MOH due to delayed payments in the NHIA complicates the relationship. (3) Role of CHAG during 2016 strike.
J17 (Olivier and Kwamie, 2017)	Journal paper	The history of public‐ (faith‐based) private health sector partnership in Ghana. Report for the Alliance for Health Policy and Systems Research, World Health Organisation: Geneva	General	Amendment of the secondment strategy between CHAG and MOH meant the majority of the staff in CHAG facilities receive their salaries from the MOH but managed and administrated by CHAG. The amendment also means that CHAG staff obtain the same conditions of service and benefits package as the GHS staff.
MB18 (The Finder, 2015)	Government Briefing	Negotiations: No invite from govt to GMA	2015	Public directed to seek care from private and QGHI that are still in operation and released a list of these facilities. Gov mentions that NHIS card holders are eligible for treatment in NHIS credentialed CHAG facilities.
MC1 (Duho, 2016)	Media Report (GNA)	Pharmacists in govt hospitals begin indefinite strike	2016	Public directed to receive pharmaceutical services from private providers and facilities.
P5 (Ghana Ministry of Health, 2015)	MOH Draft Document	Draft conditions of service for medical doctors and dentists	General	Context for 2015 strikes and the nature of the HRH crisis persistent in Ghana, and the history of this crisis.
MB70 (Joy News, 2015)	Press statement	Press statement delivered by the Minister of Health, Hon. Alex Sebgefia on the ongoing illegal strike action by the GMA on 18th August	2015	Press statement by the Minister of Health directing patients seeking care to CHAG facilities. Minister of Health notes steps taken by gov to respond to strikes, including providing resources to private facilities and re-enlisting retired doctors. Publication of list of facilities still in operation during the strike.
MB72 (Joy Online, 2015)	Media report	Christian health facilities struggle to deal with increased patient numbers	2015	Difficulties experienced by CHAG facilities during strikes. CHAG leadership advocates for resolution to the 2015 doctor strikes.
MB73 (Kojo, 2015)	Media Report	Sacked doctors are not being punished—CHAG	2015	CHAG executive leader defends decision to fire 14 medical doctors after their decision to join strikes
MA23 (Dogbevi, 2013)	Media Report	Health Ministry commends doctors for ending strike	2013	MOH statement thanking CHAG for role during health worker strikes.
MC33 (GHONE, 2016)	Media report	GHOSPA strike: CHAG to take over service delivery—MOH	2016	MOH reports that CHAG will take over service delivery of pharmacies during strike.
CB1 (Christian Health Association of Ghana, 2015a)	CHAG Communication	Support for Doctors n CHAG-member facilities within the context of the ongoing doctors’ strike action	2015	Communication sent by CHAG secretariat to CMIs to maintain services and management teams to support health workers manage the excess load.
CB2 (Christian Health Association of Ghana, 2015b)	CHAG communication	Withdrawal of OPD services by medical in government hospitals and clinics	2015	Communication sent by CHAG secretariat to CMIs to Christian identity and not embark on strikes. Requests facility heads to monitor situation and report any additional resources they may need.
MC31 (Kasapa, 2016)	Media Report	GHOSPA strike: Health Ministry directs GHS, CHAG to ensure uninterrupted pharm. services delivery	2016	MOH recognizes and affirms CHAG’s non-striking convention indicating that CHAG facilities have remained open due to their non-striking tradition.
MC16 (ClassFm, 2016)	Media Report	Nurses, midwives start ‘red week’	2016	GNRMA criticizes CHAG for workers not participating in strikes and calls on all workers to join in the strike action.
MB17 (The Finder, 2015)	Media Report	Doctors’ strike: 500 dead in 17 days	2015	HIPSAG executive director describes the challenges providers are facing with the NHIS and warns that if challenges are not addressed, private hospitals may deny NHIS subscribers services during the strike period.
MA10 (Essel, 2013)	Media Report	Doctors strike is illegal and insensitive- govt asserts	2013	Gov briefing on the 2013 GMA strike. Gov directs patients to access care at private facilities through the NHIS and lists remedial actions to ensure service continuation during strikes.
MC17 (The Finder, 2016)	Media Report	NHIA in Ghc420m arrears	2013	States NHIS indebtedness to private facilities in 2016 and outlines challenges of continuing to provide services to patients in light of the debt owed.

Gov, Government; MOH, Ministry of Health; CHAG, Christian Health Association of Ghana; QGHI, Quasi-Governmental Health Institutions; GRNMA, Ghana Registered Nurses and Midwives Association; HIPSAG, Health Insurance Service Providers Association; NHIS, National Health Insurance Scheme.

**Table 5.** Themes from embedded cases—engagement between CHAG and MOH during health worker strikes

Theme	2013 (8 Apr–8 May 2013)	2015 (30 July–2024 Aug)	2016 (5 Sep–10 Oct)
**Coordination at national level to continue service delivery**	**Patients publicly redirected to CHAG facilities**(MA20, E7, S1, J14, J15, T2) ‘Managers of health facilities must be in constant communication with QHI to ensure the delivery of seamless emergency care during the period’ **CHAG non-striking position ensures facilities remain open** MA20, E7 ‘The gov is grateful to the Police, Military, CHAG hospitals, all other health professionals and traditional medicine practitioners for the dedicated services they rendered during the strike action’ (MA20) (E7) **Continuation of services in public facilities (**E5, MA22, S3) Doctors from Cuba- ‘government is bringing in more doctors from Cuba’ (E5) **Sub-theme: Traditional Healers** ‘And traditional medicine practitioners for the dedicated services they rendered during the strike action’ (MA20, MA22)	**Patients publicly redirected to CHAG facilities (**MB44, MB18, MB70, MB71, J14, J15, T2, C1) ‘CHAG Secretariat wishes to remind all our member institutions that we are obliged and restrained by our convention, which is inspired by Christian values, ethics and unique identity, to not embark upon strike action’ (C1) ‘Friends from the media, additionally, about 25 QGHIs, 178 hospitals/clinics of CHAG, across the country are supporting in delivering healthcare’ (MB70) (MB71) **CHAG non-striking position ensures facilities remain open** MB33, MB73, T2, C1 ‘In line with our convention, inspired by Christian values, ethics and unique identity, we are obliged and restraint from embarking upon strike action to resolve matters of such nature’ (MB33) **Challenges to CHAG non-striking position** M33, C1 ‘The GMA should take them (NCHS) on. I am not amused by the stance taken by the Catholic Church. The principle that they have adopted means they are adopting slave labor in Ghana’ (MB33) **CHAG facilities difficulty managing patient load** MB44, MB72, C1 ‘Already there was pressure on us before the GMA strike and currently the situation is getting worse day in day out’ Peter Yeboah stated’ (MB72) **Continuation of services in public facilities (**MB49, MB18, MB70, MB71, MB74, MB46)	**Patients publicly redirected to CHAG facilities** MC1, MC31, J14(770), T2, P3 ‘CHAG will take over service delivery while the pharmacist strike persists’ (MC32) ‘It also directed all service delivery Agencies (GHS, CHAG, Teaching Hospitals) to comply with some interim directives whilst they try to resolve the issues’ (MC31) **CHAG non-striking position ensures facilities remain open** MC31, J14(770) ‘We are also pleased to announce to the public that CHAG Facilities are fully operational in line with their non-strike tradition’ Minister of Health (MC31) **Sub-theme: Challenges to CHAG non-striking positionE**MC16 Workers at CHAG facilities asked to stand in solidarity **Sub-theme: Continuation of services in public facilities MC20, MC21** ‘Deploying other professionals: To open space for pharmacist clerks to work, but appears contingency plans are not being followed’ (MC20) (MC21)
**Financial Protection**	**NHIS card holders assured free service at NHIS facilities** (MA14) **Challenges with NHIS system**(MA24, MA25, MA26, MA27, MA28, MA29, MA30) ‘CHAG announced medical facilities will not continue to provide services to NHIS cardholders effective Monday, March 2013. The association said that the NHIA owed its members GH50million as of 31 January 2013’ (MA25)	**NHIS card holders assured free service at NHIS facilities**(MB18, MB70, MB71, MB76) ‘NHIS card holders are eligible for treatment in 1000 NHIS0credentialed private and faith-based healthcare facilities and community-based health planning services across the country’.**Challenges with NHIS** (MB17, MB78, MC22, E54) ‘He (Executive secretary of HIPSAG) warned that should private providers deny NHIS subscribers due to indebtedness, it could result in civil strife in the country’ (MB17) ‘Private hospital denies patients with NHIS cards during strike due to debt owed to them’ (E54) **Affordability of services in CHAG facilities** (MC18) Expense of services at CHAG facilities (MC18)	**Challenges with NHIS system (**MC27) NHIS tariffs to increase by 27% **Patients turning to traditional healers (**MC19) Extend NHIS to cover herbal medicines—Monic Star Center CEO (E39) (MC24)
**Advocacy**	**CHAG acting as mediator** ‘Concerned Clergy Association of Ghana, appealing to the leadership of the GMA, to reconsider intended industrial’ (MA14)	**CHAG acting as mediator** (MB72, C1) ‘We cannot joke with people’s lives, so the FWSC, the Ministry for Employment and Labour Relations, and the GMA must come together to reach a concrete deal to halt the strike action’. ‘We are all appealing to the government and the GMA to settle the issue amicably’.	

Gov, Government; CHAG, Christian Health Association of Ghana; QHI, Quasi-governmental Health Institutions; GMA, Ghana Medical Association.

Table 5 is summary of the most important quotes that contributed to our analysis. For expanded table and sources, see **Supplementary 4 and 5**.

#### Redirection of patients to CHAG facilities

In all these strike incidents, there were instances of national-level interaction between the GoG and CHAG, with the MoH publicly directing patients to CMIs—although it is unclear to what extent patients headed that (noting other options in Ghana such as for-profit providers, traditional healers or self-medication). ‘…about 25 quasi health facilities, 178 hospitals/clinics of CHAG … are supporting in delivering healthcare’ (MB70).

Particularly during the pharmacists’ strike, it was announced that CHAG would assume service delivery responsibility as only registered pharmacists can dispense certain drugs (MC1, MC31, MC33). In a press briefing, the Minister of Health stated, ‘CHAG will take over service delivery while the pharmacist strike persists’ (MC33).

#### CHAG’s non-striking convention

CHAG maintained a non-striking position in all embedded cases, during which the CHAG Secretariat communicated to CMIs to maintain their institution’s non-striking convention. ‘CHAG Secretariat wishes to remind all our member institutions that we are obliged and restrained by our convention, which is inspired by Christian values, ethics and unique identity, to not embark upon strike action’ (CB2). And which was further affirmed by the MoH: ‘We are also pleased to announce to the public that CMIs are fully operational in line with their non-strike tradition’ (MC31, MC33).

CMIs strict adherence to this ethos led on one occasion to the dismissal of workers who chose to join the strikes. During the 2015 strike, the National Catholic Health Service (NCHS), the largest denominational sub-body within CHAG, dismissed 14 medical doctors for striking (MB33, MB73). Some, including the former Minister of Health Dr Kwaku Afiyie, viewed the NCHS’s dismissal of the striking doctors as unconstitutional. ‘I am not amused by the stance taken by the Catholic Church. The principle that they have adopted means they are adopting slave labor in Ghana’ (MB33). And there were further challenges to this position, for example in 2016 when there were appeals in the media from striking workers to CHAG workers to join the strike action. A press statement by the GNRMA stated, ‘CHAG or whatever agency, so far as you are paid by the government, you must join the action to secure your welfare’ (MC16).

#### CHAG facilities struggling to manage increased patient-load

An emerging theme was CHAG’s challenges with the increased patient load, with reports suggesting private hospitals including CMIs faced challenges managing the influx (MB72, CB1, CB2). The Executive Director of CHAG stated in news report 2015: ‘Already there was pressure on us before the GMA strike, and currently, the situation is getting worse day in and day out’.

In a communication sent from CHAG secretariat to CMIs, there was mention of the difficulty CMIs faced in managing the increasing numbers during the 2015 strike. ‘We also noted, and quite expectedly, that CMIs are recording unprecedented increases in the number of patients’ (CB1).

Beyond the verbal directives issued by the government to patients to use CMIs, it is unclear from the data whether GoG provided additional support. There were reports that the MoH provided increased resources to public and quasi-government hospitals (including assigning more nurses and midwives) (MB46, MB18), but there was no indication the same was true for CMIs.

Within CHAG, there were reported attempts to manage the rising patient load. Communication provided by CHAG secretariat to CMIs identified the following measures: supporting doctors with lunch and snacks and recalling personnel who were on leave (CB1, CB2). Management teams were instructed to request any resources they might require. However, the data doesn’t provide clarity on the extent of additional resources provided.

#### Financial protection

One of the key benefits of CMIs was their relative affordability compared with for-profit providers. During the investigated strike incidents, the GoG reportedly used the NHIS system to direct patients to these hospitals for free medical care (MA10, MB18). The NHIA’s director of communications, Selim Adonoo, stated in a press release: ‘NHIS cardholders are not just eligible in government establishments, but also in over 1000…faith-based healthcare facilities with NHIS accreditation’ (MB18).

However, it was unclear to what extent NHIS cardholders would not be required to pay costs at CMIs. A key theme was the CHAG-GoG tension due to challenges with the NHIS, such as acute delays on reimbursing health service providers (MC17). During the 2015 HWS, the government owed providers approximately GH$125 million. In 2014, CHAG created a coalition of NHIS service providers to protest 10-month-overdue claims and discontinued services to NHIS cardholders. According to some reports in 2015, private hospitals required NHIS cardholders to make a co-payment before receiving care, while others demanded payment in full due to NHIS payment delays. ‘Executive secretary of HIPSAG warned that should private providers deny NHIS subscribers due to indebtedness, it could result in civil strife…’ (MB17).

#### Participate in advocacy to encourage resolution to strikes

An emerging theme was CHAG’s advocacy role. During the 2015 trikes, CHAG leadership called for an end to the strikes. The executive director of CHAG stated: ‘We cannot joke with people’s lives, so the FWSC……and the GMA must come together……to halt the strike action’ (MB72).

### Factors influencing CHAG–GoG engagement during strikes

Table 6 describes key features of the CHA–State PPE model related themes based on our findings.

**Table 6.** Themes CHA–State public–private engagement

Feature	Themes
Transfer of decision-making responsibilities	The ability of CHAG to remain autonomous was a central theme. This autonomy allows CHAG to act as a collaborator and critique of gov. Implications of integration in gov system (especially over HRH) for autonomy.
Hierarchical relationship—public sector buys services	MOH is still the highest decision-making body: it issues directives for agencies during strikes. CHAG is expected to act within the policies and framework of the MOH – CHAG’s policy mission is to complement public system.
No knowledge transfer due to low levels of collaboration	Low levels of collaboration during health worker strikes; lack of evidence to suggest a formal collaboration. Limited evidence of knowledge sharing. In non-strike periods evidence of extensive collaboration and knowledge sharing.
Sharing of financial infrastructure and/or human resources	The nature of HRH management between CHAG and MOH. Adaption of the secondment policy. Conditions of service of CHAG workers unclear. Gov funding of staff salaries in CMIs. Unclear sharing of resources during strike incidents outside of verbal directives to CHAG facilities.
Private partner paid directly by public authority; not necessarily performance-based	NHIS is central to CHAG operations. Challenges with NHIS: delayed payments and poor accreditation.
Long-term contract	CHA–State PPE formalized by MOU. Long-term relationship between CHAG and MOH—a partnership built on trust and shared values. CHAG seen as a partner of MOH.

Gov, Government; NHIS, National Health Insurance Scheme; CHAG, Christian Health Association of Ghana; HRH, human resources for health (source adapted from Whyle and Olivier, 2017).

#### Trust and shared values

A key aspect of the CHA–State PPE is its relational engagement, with CHAG–GoG PPE seen by respondents^7^ as rooted in trust and shared values, making CHAG a dependable partner for MoH during HWS. This trust was attributed to CHAG’s long-standing, reliable and innovative reputation. ‘In my time, the trust increased as we were part of the decision-making process at the national level’ (Respondent F).

#### Autonomy and collaboration

The CHA–State PPE often shows limited collaboration and knowledge sharing, illustrated during HWS. Although CHAG and MoH collaborated extensively on other policies like the NHIS, during strikes, apart from government directives for CHAG to maintain service delivery, there isn’t much evidence of broader collaboration or knowledge sharing.

As a service agency of the MoH, CHAG was to follow MoH directives during HWS to continue service delivery. Adhering to Ghana’s laws and policies, CMIs may have faced pressure to remain operational during strikes, as the government often views them as illegal. CHAG needs to balance its agency role with its workers’ interests, who are often part of professional unions with grievances similar to state institution workers. The perception of CHAG ‘selling out’ can intensify during HWS, with some in the public sector viewing CHAG’s non-striking stance as a betrayal. ‘In times of strike action, CHAG remains open. Seen as traitors – but the MoH them sees us as partners and says well done’ (Respondent H).

An emerging theme was the instances of strikes by CHAG. Some respondents commented on CHAG staff strikes in the 1980s by health workers to receive the same benefits as those in public facilities. ‘So, there were later open strikes in early and mid-1980s, when people saw that the government workers got more than MBP HWs’ (Respondent G). Respondents also spoke of the instance in 2014 when CMIs ceased services to NHIS cardholders ‘Now, with NHIS, we’ve withdrawn services—we’ve striked—and that has fatally affected things, people’ (Respondent F).

Respondents also acknowledged CHAG’s organizational complexity and the diversity of its entities and actors. Moreover, the non-striking ethos of CHAG appears to be a practice that has evolved over the years as opposed to a contractual agreement between CHAG and health workers in CMIs. The communication sent out by CHAG to CMIs stated: ‘Our principles and ethical considerations as a Christian Health Service encourages health workers to remain at post in matters of industrial disputes. Over the years, health workers within CHAG have been very kind to heed to this principle’ (CB1).

#### CHAG–GoG balance of power

Power and the dynamics of negotiation were recurring themes. Respondents discussed the years of negotiations between the two actors, notably regarding HRH management. ‘CHAG is always at the table. Even with negotiations with health partners – it is always GHS, MoH and CHAG’ (Respondent A).

The CHAG–MoH engagement significantly depended on the personal power of specific actors, with relationship strength varying with the leadership. ‘Occasionally there are issues of mistrust – it depends on who is in charge, leadership’ (Respondent E). For instance, one respondent appreciated a leader’s charismatic decision to deny services to NHIS cardholders, which enhanced trust towards the institution.

The nature of the CHA–GoG MOU also affects the balance of power. The CHAG–MoH MOU **(Box 1)** provides that either party may cancel the agreement by providing a 3-month notice. Even the fact that CHAG employees receive the same service conditions as GHS employees is, according to Yeboah and Buckle (2017), a ‘partnership practice’ and not a contractual requirement. The absence of enforceability mechanisms may affect the balance of power between the two actors. Yeboah and Buckle (2017) comment that in some instances when CHAG played more of a critical role in the relationship—i.e. the withdrawal of services to the NHIS card holders—there were subtle threats from the GoG to withdraw financial support. However, some respondents saw CHAG’s ‘non-striking convention’ as a significant negotiating power. ‘And CHAG does not go on strike, that is one big negotiating power’ (Respondent C).

#### Sharing of resources

Managing HRH between CHAG and the GoG was an essential element of the PPE during strikes. At the time, the GoG covered 90% of CMI staff salaries, though CHAG staff aren’t MoH employees. The CHAG Secretariat handles administrative matters related to employment in consultation with MoH. To attract health personnel to remote areas, CHAG offers additional benefits for staff like fuel and housing allowances.

Another theme that emerged was the evolution of the secondment policy between CHAG and GoG. In the 2000s, GoG implemented a new HR management process through the secondment policy which meant that government-paid personnel recruited by the MoH/GHS were assigned to positions in CMIs. CHAG management and the seconded employees experienced several challenges with the secondment policy. Some facility managers felt the seconded staff lacked discipline and could not adhere to the institution’s organizational culture. After significant negotiation between CHAG and the GoG, the secondment policy was phased out over time (although there was no formal cancellation of the policy). ‘Secondment – we scrapped that – it was an HR and management decision, way before CHAG got rid of it, we stopped it … the identity and commitment was terrible’ (Respondent I).

The amended secondment policy may contribute to contention over how employees identify in CMIs (as government or CHAG staff). It may also influence the tensions observed in strike incidents when CHAG staff wish to join their striking counterparts.

## Discussion

Our findings suggest that HWS in Ghana were a chronic system stressor. Shocks are sudden and unexpected event that severely disrupts the functioning of the health system while stressors refer to ongoing or chronic challenges that strain the capacity and functionality of the health system (Barasa *et al*., 2017). HWS were rarely unexpected and usually followed prolonged failed negotiations with the government over long-term HR management issues like service conditions, delayed premium payments and salary disputes, aligning with previous studies in Ghana (Gyamfi, 2011; Awori and Tettey-Enyo, 2015; Ampofo *et al*., 2022) and findings in other LMICs (Russo *et al*., 2019). Strikes were exacerbated by political tensions from contentious elections aggressive language from officials and diminished trust from the government’s perceived neglect of agreements. Similarly, health workers in Nigeria often went on strike because they felt their concerns were not genuinely addressed by the government (Oleribe *et al*., 2016).

HWS in Ghana often stemmed from legislative changes with unintended consequences, especially issues with the SSSS. This policy, though well-intended, amplified the public sector wage bill, exacerbating slow economic growth and a high budget deficit. Agyepong *et al*. (2012) highlighted a similar pattern where an initial policy meant to address doctor strikes inadvertently led to more strikes—highlighting the complex adaptive nature of health systems.

PPE in health is vital for maintaining services when public sector halts, seen during Ghana’s strikes. Workers withdrew emergency services in 6 of the 10 strike incidents analysed. In the global literature, the continuation of emergency services was cited as one of the primary resilience mechanisms that led to constant or decreased mortality rates (Cunningham *et al*., 2008; Metcalfe *et al*., 2015). The withdrawal of emergency services is thus a concerning finding, making continuing services in private hospitals all the more crucial. Similarly, the withholding of emergency services was cited as a major concern in Kenya (Njuguna, 2015; Ong’ayo *et al*., 2019).

Given that HWS in Ghana may be viewed as a chronic system stressor, there is a need to nurture everyday resilience. Building on the concept of ‘everyday resilience’, Kagwanja *et al*.’s (2020) conceptual framework integrates two major concepts in resilience thinking: ‘resilience strategies’ (which can be absorptive, adaptive or transformative) and ‘resilience capacities’ (contextual, behavioural or cognitive). The question then is when a resilience strategy is beneficial to the health system. According to Chima *et al*. (2013), the goal is for ‘ethical’ strikes in which effective responses safeguard the poorest households from health-related financial losses and, at the very least, preserve core and emergency services.

This case study found evidence of system-level interactions between CHAG and the GoG to maintain service continuity during strikes which allowed for mostly ‘absorptive’ resilience strategies, including public announcements encouraging patients to seek care in CMIs. In contrast to studies exploring health system resilience during strikes in Kenya (Waithaka *et al*., 2020; Scanlon *et al*., 2021a; 2021b; Adam *et al*., 2018), these interactions were not ad-hoc, taken on by individual frontline managers and inconsistent but occurred on a national level.

However, the population’s use of these services and the extent to which CMIs could continue providing quality services is questionable. There is limited evidence of government actions to assist CMIs during HWS. Evidence also suggests that the rising patient load sometimes overburdened CMIs. A study by Adam *et al*. (2018) at a FBHP noted an increase in paediatric and obstetric mortality rates during a 2017 doctor strike in Kenya—raising concern about private facility’s ability to provide quality care when overburdened.

Though it was noted that CHAG lobbied its social capital to advocate for conclusion of the strikes, there was limited evidence of further ‘adaptive’ and ‘transformative’ strategies. The approach to strikes throughout the time analysed followed the same pattern, with little indication of the system becoming better at preventing or responding to strikes. We could not find any mention of HWS in key policy documents in Ghana. There was scant indication of the government’s preparedness for future strike incidents.

CHAG–GoG engagement also conferred important resilience capacities that the system could draw on during strikes. The ability of the government to use the NHIS as a mechanism to protect some individuals from paying excessive costs in CMIs can be regarded as a ‘contextual capacity’ (aiding the health system to absorb the shock). However, there were significant structural issues with the NHIS, including conflicts between CHAG and the GoG over unpaid claims and some reports of private facilities denying services to NHIS cardholders as a result.

Moreover, CMIs remained open due to the ‘non-striking convention’ of the organization which can arguably be regarded as a ‘cognitive capacity’. The consistency of CHAG’s convention and approach to strikes may also be regarded as a ‘behavioural capacity’ as behavioural capacities rely partly on the development of learned routines that provide a first response to a shock or stressor (Lengnick-Hall *et al*., 2011). However, stating CHAG has a ‘non-striking convention’ may be an oversimplification and not capture the nuances of interaction and views within the vast and complex organization. In some instances, CHAG secretariat itself had engaged in forms of protest when it deemed necessary, i.e. withdrawal of services to NHIS card holders.

### Implications of (and for) the CHA–State PPE

Our findings also further our understanding of the CHA–State model (Whyle and Olivier, 2017) and the implications of this model for health systems resilience. The CHA–State model, as shown in the context of Ghana, is founded on trust, shared values and the nature of the long-term interaction between the two actors. Whyle and Olivier (2017) argue that there is an atypical balance of power between CHAs and their respective governments due to their mutual dependence. Some respondents in the larger AHPSR study highlighted that CHAG’s non-striking position is an essential negotiating instrument for the organization, sometimes used to gain certain benefits from the government. This mutual dependence between CHAGs and their governments explains this relationship’s absence of institutional accountability mechanisms (Whyle and Olivier, 2016). Some have noted the CHA–State model’s lack of enforceability to be a strength (Boulenger *et al*., 2014). However, important elements of fragility within that balance may need to be considered. For example, the reporting of subtle indications from the government to withdraw funding when CHAG takes on more of a critical position. Noting the lack of binding agreements in CHAG–MoH MOU, these threats could materialize without legal ramifications.

Though effective integration of private actors, particularly FBPHs, is cited as essential for resilience, it has been argued that increased interdependencies between FBHPs and the public sector have the potential to render health systems more vulnerable and less resilient (Chirwa *et al*., 2013). The concern is that increased integration may expose the private sector to the same challenges as the public sector. The amended secondment policy meant that CHAG staff were on the government payroll and were also affected by salary disputes and contestations in the public sector. It also meant that some (including some CHAG staff) might view CHAG employees as public sector employees and the non-striking position as a betrayal of their interests. Some union members have strongly criticized CHAG for discouraging staff participation in strikes. These findings are relevant for several countries in Africa as the issue of secondment has been contentious, with many nations experiencing difficulties with the implementation and others scraping the practice entirely (Dimmock *et al*., 2012).

In addition, Gilson (2012) notes that policies are not just formal written policies but the actual activities on the ground that become policy. This can be seen in both the amendment of the secondment policy and the non-striking convention of CHAG, which are both not documented and formal policy positions. The implication for HWS is the extent to which CHAG can continue to enforce this position on staff members who may seek to join strikes.

### Relevance of findings for Ghana post-2016

HWS remain a persistent chronic stressor in Ghana. In recent years, there have been strikes by other professional unions, such as physician assistants (ClassFM, 2018a; KasapaFM, 2018b) and anaesthetists (Ghana News Agency, 2020), reportedly over similar HRH challenges. Like before, CHAG sent communications to CMIs to remain open (Christian Health Association of Ghana, 2020). Tensions with CHAG’s non-stirking convention continue. In 2020, the Ghana Registered Nurses and Midwives Association (GRNMA) association attorneys responded harshly to CHAG’s convention insisting that CHAG’s behaviour showed it was ‘in bed with the government’ (Appiah, 2020).

Though there have been some notable improvements in the accreditation of providers and registration of the population onto the NHIS, there are still reports of frequently delayed payments to providers (Okoroh *et al*., 2018). In recent strikes, CHAG has taken more of a mediatory role. In 2020, CHAG facilitated meetings between striking anaesthetists and the MoH, which was acknowledged as a major factor in the strike’s conclusion (Annan, 2022; Arhinful, 2022).

### Limitations

The research was notably affected by the scarce investigations on HWS in LMICs, prompting a recommendation for future studies to explore patient behaviour during strikes and evaluate the broader systemic impacts of HWS. The scope of this study was limited to exploring the macro-level CHAG–MoH interactions and could go much further into meso- and micro-level resiliencies strategies. While all quality controls were practised, this study was hampered by the scarcity of available evidence and the resulting reliance on grey materials such as media reports. Future research with a broader scope, which could, for example, gather primary evidence on ‘closed-door’ HWS negotiations, would be highly beneficial.

## Conclusions

Though our results were unique to Ghana and CHAG–GoG PPE, we believe there are important takeaways that can be used to guide future strategies regarding strikes and PPE arrangements.

While addressing the root HRH challenges to prevent strikes should be a priority for governments, relying solely on this is no longer viable as health systems may not self-correct. Our study shows persistent strikes in Ghana, some lasting nearly 2 months, underline a need for better preparedness and response policies at all government levels to ensure service continuity during strikes. These policies should consider both hardware and software factors contributing to strikes, like aggravating language, to foster ‘everyday resilience’ against future and prolonged HWS.

In cultivating resilience during strikes, our study highlights the key role partnerships with private providers (particularly FBHPs) can contribute. The engagement between CHAG and GoG provided important resilience capacities and strategies that the health system could draw on. We found evidence of system-level interactions between CHAG and the GoG during strikes that allowed for adaptive and absorptive strategies. This interaction was partly attributed to the unique CHA–State PPE and amended secondment policy in addition to CHAG’s non-striking convention.

In ensuring preparedness for future strikes, we identified important challenges and complexities within the CHAG–GoG engagement that need to be addressed. There was limited evidence to suggest that the GoG provided additional resources and support to private facilities. There is significant room for the government to ensure adequate support of private facilities during strikes and for innovative adaptive and transformative strategies.

Our analysis showed the importance of the NHIS as a link between Ghana’s public and private health systems, allowing patients to seek care in the private sector when public services are interrupted. This is pertinent for numerous African nations that have or are contemplating contributory health insurance programmes. The challenges facing the scheme in Ghana concerning late payment of suppliers also highlight the fragility of these schemes and the necessity for system-based initiatives to enhance these schemes towards the objective of Universal Health Coverage.

There are also important considerations for CHAG—and similar private providers. CHAG must assess the type of support CMIs require during strikes and proactively seek collaboration with government to ensure this support exists. CHAG also needs to consider the challenges to its non-striking position and may find it beneficial to engage in open dialogue with CMI workers to find ways for health workers to demonstrate solidarity with public sector workers while ensuring the continuation of essential health services. We recommend that CHAG utilize the software features of its engagement with the GoG (trust, negotiation and innovative skills) to expand its role as an advocate and mediator between the government and health workers for improved working conditions to contribute to transformative strategies.

Lastly, in averting strikes, we recommend government proactively and, with a keen appreciation of the complex adaptive nature of the health system, build collaborative solutions with professional bodies and other stakeholders (including the private sector) to manage HRH-related challenges in the nation.

## Supplementary Material

czae018_Supp

## Data Availability

Data are available in a repository and can be accessed via a DOI link (submitted now as supplementary data).

## Footnotes

1. In this paper, the term ‘private health sector’ refers to entities engaged in delivering health services, operating independently from the government’s direct oversight.

2. FBHPs refer to a grouping of non-state providers identifying as faith aligned and usually non-profit in configuration (Olivier *et al*., 2015). FBHPs refer to health facilities that provide health services, along with their governing bodies, linking networks and affiliated entities (Whyle and Olivier, 2017).

3. For the purposes of this study, we only focused on health workers who have direct contact with patients such as doctors, nurses, physician assistants and pharmacists.

4. Quasi-Government Health Institutions consist of government institutions, agencies and departments whose primary focus is not on health but which engage in health service operations, such as Ghana’s ports and the military (Ghana Ministry of Health, 2020).

5. In 1975, the GoG commissioned a study on the role of FBHPPs in the health system. The resultant ‘Adibo report’ presented three key options for the government in how it could engage with the FBHPs, including taking over the hospitals entirely, providing partial support with the possibility of taking over in future and allowing FBHPs to operate independently but with the support of government. Following the report, the GoG decided to continue collaboration with FBHPs, allowing them to operate independently. The report also suggested that the government subsidize the salaries of FBHP staff (Yeboah and Buckle, 2017; Grieve and Olivier, 2018).

6. Engagement refers to an interaction or involvement between actors. Thus, we use the term engagement and interactions interchangeably.

7. Respondents here refer individuals who took part in the AHPSR interviews—secondary analyses of the transcripts were used for this study (see Methods).

## Reflexivity statement

Reflecting on the journal’s commitment to diminishing inequities in global health research, we have introspectively examined the composition of our author group in terms of gender, seniority and regional location.

- Seniority: Our duo consists of one senior researcher and one early-career researcher, fostering a rich inter-generational dialogue and mentorship throughout the research journey. The senior researcher infused the process with extensive experience and profound contextual understanding, while the early-career researcher introduced fresh perspectives and innovative analytical approaches.
- Regional Location: The corresponding author has a bicultural identity, being Ghanaian and South African, with lived experiences in both nations. The supervising author, a South African, boasts extensive research experience within the Ghanaian health system. This geographical and cultural diversity enriched our discussions, ensuring our analysis was grounded in local realities while being informed by a broader, global context.
- Gender: Acknowledging the small size of our research team, it is pertinent to note that both authors identify as women. Our gendered perspectives, while not encompassing the full spectrum of gender identities, brought a unique lens through which we approached our research, aligning with the broader goal of promoting gender representation in global health research.
