# Supplementary material for: Public–Private engagement and health systems resilience in times of health worker strikes: a Ghanaian case study
Source: Health Policy Plan. 2024 Mar 18;39(5):469–85. doi: 10.1093/heapol/czae018 (PMC11095267; doi:10.1093/heapol/czae018)
Supplement: czae018_Supp [file czae018_supp.zip › Supplementary 2. Unique identifiers.docx]

## Supplementary 2: Unique identifier sources

| **Unique Identifier** | **Article Title** | **Date** | **Source/Author** | **Links/Citation** |
| --- | --- | --- | --- | --- |
| MA1 | Doctors in Ghana continue to strike over salary dispute | 12/04/2013 | BBC News | <https://www.bbc.com/news/av/world-africa-22128960> |
| MA2 | Ghana: GMA Reacts To Government's Position On Doctors' Strike | 12/04/2013 | All Africa | <https://allafrica.com/stories/201304150896.html> |
| MA3 | Ghana's public doctors call of one month strike | 08/05/2013 | Ghana Business news | [https://www.ghanabusinessnews.com/2013/05/08/ghana](https://www.ghanabusinessnews.com/2013/05/08/ghanas-public-doctors-call-off-one-month-strike/) [s-public-doctors-call-off-one-month-strike/](https://www.ghanabusinessnews.com/2013/05/08/ghanas-public-doctors-call-off-one-month-strike/) |
| MA4 | Ghana hit by waves of public sector strikes | 25/04/2013 | Equal Time | [https://www.equaltimes.org/ghana-hit-by-wave-of-public-](https://www.equaltimes.org/ghana-hit-by-wave-of-public-sector-strikes?lang=en&.Y0wHR6hBxpQ) [sector-strikes?lang=en#.Y0wHR6hBxpQ](https://www.equaltimes.org/ghana-hit-by-wave-of-public-sector-strikes?lang=en&.Y0wHR6hBxpQ) |
| MA5 | Doctors strike, never again | 11/05/2013 | Ghana News (opinion) | <https://newsghana.com.gh/doctors-strike-never-again/> |
| MA6 | Doctors Call of Strike | 07/05/2013 | Joy News | <https://www.myjoyonline.com/doctors-call-off-strike/> |
| MA7 | Ghana Doctors Strike, Kofi Annan intervenes | 24.04/2013 | IGIHE | [https://en.igihe.com/health/ghana-doctors-strike-kofi-](https://en.igihe.com/health/ghana-doctors-strike-kofi-annan-intervenes) [annan-intervenes](https://en.igihe.com/health/ghana-doctors-strike-kofi-annan-intervenes) |
| MA8 | Doctors declare another strike, pharmacists set to follow | 07/04/2013 | Berekumcity.com | [https://www.berekumcity.com/news/national-](https://www.berekumcity.com/news/national-news/doctors-declare-another-strike-pharmacists-set-to-follow) [news/doctors-declare-another-strike-pharmacists-set-to-](https://www.berekumcity.com/news/national-news/doctors-declare-another-strike-pharmacists-set-to-follow)  [follow](https://www.berekumcity.com/news/national-news/doctors-declare-another-strike-pharmacists-set-to-follow) |
| MA9 | Ghana federation of labour slams doctors strike action | 30/04/2013 | Peace FM | [https://www.peacefmonline.com/pages/local/news/2013](https://www.peacefmonline.com/pages/local/news/201304/162785.php) [04/162785.php](https://www.peacefmonline.com/pages/local/news/201304/162785.php) |
| MA10 | Doctors strike is illegal and insensitive- govt asserts | 10/04/2013 | Myjoyonline.com | [https://www.modernghana.com/news/457729/doctors-](https://www.modernghana.com/news/457729/doctors-strike-is-illegal-and-insensitive-govt-asserts.html) [strike-is-illegal-and-insensitive-govt-asserts.html](https://www.modernghana.com/news/457729/doctors-strike-is-illegal-and-insensitive-govt-asserts.html) |
| MA11 | Doctors strike in the offing if | 06/04/2013 | Kessben | [https://kessbenonline.com/2013/04/06/doctors-strike-in-](https://kessbenonline.com/2013/04/06/doctors-strike-in-the-offing-if/) [the-offing-if/](https://kessbenonline.com/2013/04/06/doctors-strike-in-the-offing-if/) |
| MA12 | Chief of Staff meets parties to the doctors strike | 13/02/2013 | Joy Online | [http://www.justiceghana.com/index.php/en/2012-01-24-](http://www.justiceghana.com/index.php/en/2012-01-24-13-46-34/6262-doctors-end-strike-action-resume-work-on-Wednesday)  [13-46-34/6262-doctors-end-strike-action-resume-work-](http://www.justiceghana.com/index.php/en/2012-01-24-13-46-34/6262-doctors-end-strike-action-resume-work-on-Wednesday) [on-Wednesday](http://www.justiceghana.com/index.php/en/2012-01-24-13-46-34/6262-doctors-end-strike-action-resume-work-on-Wednesday) |
| MA13 | GHANA: DOCTORS STRIKE-WHY AGAIN? | 22/04/2013 | Kobby Blay | [https://kobbyblay.wordpress.com/2013/04/22/ghana-](https://kobbyblay.wordpress.com/2013/04/22/ghana-doctors-strike-why-again/)  [doctors-strike-why-again/](https://kobbyblay.wordpress.com/2013/04/22/ghana-doctors-strike-why-again/) |
| MA14 | Clergy pleads with doctors to call off intended strike | 08/02/2013 | Joy Online | [https://www.myjoyonline.com/clergy-pleads-with-](https://www.myjoyonline.com/clergy-pleads-with-doctors-to-call-off-intended-strike/) [doctors-to-call-off-intended-strike/](https://www.myjoyonline.com/clergy-pleads-with-doctors-to-call-off-intended-strike/) |
| MA15 | Patient surge as Ghana’s doctors strike | 21/04/2013 | Buisiness Recorder | <https://fp.brecorder.com/2013/04/201304211176962/> |
| MA16 | Ghana’s Budget Deficit Target Under Threat Amid Strikes | 18/04/2013 | Bloomberg | [https://www.bloomberg.com/news/articles/2013-04-](https://www.bloomberg.com/news/articles/2013-04-18/ghanaian-public-workers-strike-may-threaten-deficit?leadSource=uverify%20wall)  [18/ghanaian-public-workers-strike-may-threaten-](https://www.bloomberg.com/news/articles/2013-04-18/ghanaian-public-workers-strike-may-threaten-deficit?leadSource=uverify%20wall) [deficit?leadSource=uverify%20wall](https://www.bloomberg.com/news/articles/2013-04-18/ghanaian-public-workers-strike-may-threaten-deficit?leadSource=uverify%20wall) |

| MA17 | Public sector strikes test Ghana's deficit-cutting mettle | 16/04/2013 | Reuters | [https://www.reuters.com/article/ghana-strikes-](https://www.reuters.com/article/ghana-strikes-idUSL3N0D20RA20130416) [idUSL3N0D20RA20130416](https://www.reuters.com/article/ghana-strikes-idUSL3N0D20RA20130416) |
| --- | --- | --- | --- | --- |
| MA19 | Doctors warn Nunoo-Mensah to be mindful of lis language | 21/10/2013 | Ghana Web Database | [https://www.ghanaweb.com/GhanaHomePage/NewsArchi](https://www.ghanaweb.com/GhanaHomePage/NewsArchive/Doctors-warn-Nunoo-Mensah-to-be-mindful-of-lis-language-289491)  [ve/Doctors-warn-Nunoo-Mensah-to-be-mindful-of-lis-](https://www.ghanaweb.com/GhanaHomePage/NewsArchive/Doctors-warn-Nunoo-Mensah-to-be-mindful-of-lis-language-289491) [language-289491](https://www.ghanaweb.com/GhanaHomePage/NewsArchive/Doctors-warn-Nunoo-Mensah-to-be-mindful-of-lis-language-289491) |
| MA20 | Health Ministry commends doctors for ending strike | 08/05/2013 | Ghana Business news | [https://www.ghanabusinessnews.com/2013/05/08/health](https://www.ghanabusinessnews.com/2013/05/08/health-ministry-commends-doctors-for-ending-strike/)  [-ministry-commends-doctors-for-ending-strike/](https://www.ghanabusinessnews.com/2013/05/08/health-ministry-commends-doctors-for-ending-strike/) |
| MA21 | Government to bring in more Cuban doctors | 30/04/2013 | Joy Online | [https://www.myjoyonline.com/government-to-bring-in-](https://www.myjoyonline.com/government-to-bring-in-more-cuban-doctors/)  [more-cuban-doctors/](https://www.myjoyonline.com/government-to-bring-in-more-cuban-doctors/) |
| MA22 | Doctors’ strike hits Komfo Anokye hard, worst in 59-year history of hospital | 06/05/2013 | Graphic.com | [https://www.graphic.com.gh/news/health/doctors-strike-](https://www.graphic.com.gh/news/health/doctors-strike-hits-komfo-anokye-hard-worst-in-59-year-history-of-hospital.html) [hits-komfo-anokye-hard-worst-in-59-year-history-of-](https://www.graphic.com.gh/news/health/doctors-strike-hits-komfo-anokye-hard-worst-in-59-year-history-of-hospital.html)  [hospital.html](https://www.graphic.com.gh/news/health/doctors-strike-hits-komfo-anokye-hard-worst-in-59-year-history-of-hospital.html) |
| MA23 | Ministry commends doctors for ending strike | 08/05/2013 | Peace FM | [https://www.peacefmonline.com/pages/local/health/201](https://www.peacefmonline.com/pages/local/health/201305/163690.php)  [305/163690.php](https://www.peacefmonline.com/pages/local/health/201305/163690.php) |
| MA24 | Mahama's economic policy inspires strike action - O. B Amoah | 04/04/2013 | Ghana Web | [https://www.ghanaweb.com/GhanaHomePage/business/](https://www.ghanaweb.com/GhanaHomePage/business/Mahama-s-economic-policy-inspires-strike-action-O-B-Amoah-269930)  [Mahama-s-economic-policy-inspires-strike-action-O-B-](https://www.ghanaweb.com/GhanaHomePage/business/Mahama-s-economic-policy-inspires-strike-action-O-B-Amoah-269930) [Amoah-269930](https://www.ghanaweb.com/GhanaHomePage/business/Mahama-s-economic-policy-inspires-strike-action-O-B-Amoah-269930) |
| MA25 | Missionary Hospitals will treat NHIS patients | 11/03/2013 | Joy FM | [https://www.ghanaweb.com/GhanaHomePage/NewsArchi](https://www.ghanaweb.com/GhanaHomePage/NewsArchive/Missionary-Hospitals-will-treat-NHIS-patients-267317) [ve/Missionary-Hospitals-will-treat-NHIS-patients-267317](https://www.ghanaweb.com/GhanaHomePage/NewsArchive/Missionary-Hospitals-will-treat-NHIS-patients-267317) |
| MA26 | BoasiakoCash and Carry system back in B/A from Monday | 08/03/2013 | Ghana Web | [https://www.ghanaweb.com/GhanaHomePage/health/Cas](https://www.ghanaweb.com/GhanaHomePage/health/Cash-and-Carry-system-back-in-B-A-from-Monday-267009) [h-and-Carry-system-back-in-B-A-from-Monday-267009](https://www.ghanaweb.com/GhanaHomePage/health/Cash-and-Carry-system-back-in-B-A-from-Monday-267009) |
| MA27 | Missionary hospitals restore services to Health Insurance card holders | 15/03/2013 | Ghana Web | [https://www.ghanaweb.com/GhanaHomePage/NewsArchi](https://www.ghanaweb.com/GhanaHomePage/NewsArchive/Missionary-hospitals-restore-services-to-Health-Insurance-card-holders-267789) [ve/Missionary-hospitals-restore-services-to-Health-](https://www.ghanaweb.com/GhanaHomePage/NewsArchive/Missionary-hospitals-restore-services-to-Health-Insurance-card-holders-267789) [Insurance-card-holders-267789](https://www.ghanaweb.com/GhanaHomePage/NewsArchive/Missionary-hospitals-restore-services-to-Health-Insurance-card-holders-267789) |
| MA28 | NHIA pays outstanding claims | 23/03/2013 | Ghana News Agency | [https://www.ghanaweb.com/GhanaHomePage/health/NH](https://www.ghanaweb.com/GhanaHomePage/health/NHIA-pays-outstanding-claims-268759) [IA-pays-outstanding-claims-268759](https://www.ghanaweb.com/GhanaHomePage/health/NHIA-pays-outstanding-claims-268759) |
| MA29 | Missionary hospitals will refuse NHIS patients | 10/03/2013 | Joy Online | [https://www.ghanaweb.com/GhanaHomePage/NewsArchi](https://www.ghanaweb.com/GhanaHomePage/NewsArchive/Missionary-hospitals-will-refuse-NHIS-patients-267207) [ve/Missionary-hospitals-will-refuse-NHIS-patients-267207](https://www.ghanaweb.com/GhanaHomePage/NewsArchive/Missionary-hospitals-will-refuse-NHIS-patients-267207) |
| MA30 | Current state of NHIS is very frightening – Dr. Anane | 11/03/2013 | Joy Online | [https://www.ghanaweb.com/GhanaHomePage/NewsArchi](https://www.ghanaweb.com/GhanaHomePage/NewsArchive/Current-state-of-NHIS-is-very-frightening-Dr-Anane-267352)  [ve/Current-state-of-NHIS-is-very-frightening-Dr-Anane-](https://www.ghanaweb.com/GhanaHomePage/NewsArchive/Current-state-of-NHIS-is-very-frightening-Dr-Anane-267352) [267352](https://www.ghanaweb.com/GhanaHomePage/NewsArchive/Current-state-of-NHIS-is-very-frightening-Dr-Anane-267352) |
| MB1 | Patients happy to meet doctors as they resume work | 24/08/2015 | tv3network.com | [https://3news.com/patients-happy-to-meet-doctors-as-](https://3news.com/patients-happy-to-meet-doctors-as-they-resume-work/) [they-resume-work/](https://3news.com/patients-happy-to-meet-doctors-as-they-resume-work/) |
| MB2 | We're resuming duty with a heavy heart - Doctors | 24/08/2015 | starrfmonline.com | [https://www.ghanaweb.com/GhanaHomePage/NewsArchi](https://www.ghanaweb.com/GhanaHomePage/NewsArchive/We-re-resuming-duty-with-a-heavy-heart-Doctors-377267)  [ve/We-re-resuming-duty-with-a-heavy-heart-Doctors-](https://www.ghanaweb.com/GhanaHomePage/NewsArchive/We-re-resuming-duty-with-a-heavy-heart-Doctors-377267) [377267](https://www.ghanaweb.com/GhanaHomePage/NewsArchive/We-re-resuming-duty-with-a-heavy-heart-Doctors-377267) |
| MB3 | GMA leadership to be impeached over strike | 24/08/2015 | starrfmonline.com | [https://www.ghanaweb.com/GhanaHomePage/NewsArchi](https://www.ghanaweb.com/GhanaHomePage/NewsArchive/GMA-leadership-to-be-impeached-over-strike-377341) [ve/GMA-leadership-to-be-impeached-over-strike-377341](https://www.ghanaweb.com/GhanaHomePage/NewsArchive/GMA-leadership-to-be-impeached-over-strike-377341) |
| MB4 | Doctors to sign conditions of service Sept. 30 | 22/08/2015 | starrfmonline.com | [https://www.ghanaweb.com/GhanaHomePage/NewsArchi](https://www.ghanaweb.com/GhanaHomePage/NewsArchive/Doctors-to-sign-conditions-of-service-Sept-30-376968) [ve/Doctors-to-sign-conditions-of-service-Sept-30-376968](https://www.ghanaweb.com/GhanaHomePage/NewsArchive/Doctors-to-sign-conditions-of-service-Sept-30-376968) |
| MB5 | Doctors to receive August salaries despite strike | 22/08/2015 | starrfmonline.com | [https://www.ghanaweb.com/GhanaHomePage/NewsArchi](https://www.ghanaweb.com/GhanaHomePage/NewsArchive/Doctors-to-receive-August-salaries-despite-strike-376982)  [ve/Doctors-to-receive-August-salaries-despite-strike-](https://www.ghanaweb.com/GhanaHomePage/NewsArchive/Doctors-to-receive-August-salaries-despite-strike-376982) [376982](https://www.ghanaweb.com/GhanaHomePage/NewsArchive/Doctors-to-receive-August-salaries-despite-strike-376982) |

| MB6 | Doctors slam Nunoo-Mensah over football comment | 21/08/2015 | starrfmonline.com | [https://www.ghanaweb.com/GhanaHomePage/NewsArchi](https://www.ghanaweb.com/GhanaHomePage/NewsArchive/Doctors-slam-Nunoo-Mensah-over-football-comment-376833) [ve/Doctors-slam-Nunoo-Mensah-over-football-comment-](https://www.ghanaweb.com/GhanaHomePage/NewsArchive/Doctors-slam-Nunoo-Mensah-over-football-comment-376833)  [376833](https://www.ghanaweb.com/GhanaHomePage/NewsArchive/Doctors-slam-Nunoo-Mensah-over-football-comment-376833) |
| --- | --- | --- | --- | --- |
| MB7 | Doctors’ strike: House of Chiefs meets GMA, FWSC | 21/08/2015 | starrfmonline.com | [https://www.ghanaweb.com/GhanaHomePage/NewsArchi](https://www.ghanaweb.com/GhanaHomePage/NewsArchive/Doctors-strike-House-of-Chiefs-meets-GMA-FWSC-376845)  [ve/Doctors-strike-House-of-Chiefs-meets-GMA-FWSC-](https://www.ghanaweb.com/GhanaHomePage/NewsArchive/Doctors-strike-House-of-Chiefs-meets-GMA-FWSC-376845) [376845](https://www.ghanaweb.com/GhanaHomePage/NewsArchive/Doctors-strike-House-of-Chiefs-meets-GMA-FWSC-376845) |
| MB8 | Doctors end strike, go back to work on Monday | 21/08/2015 | Citi FM Online | [https://www.ghanaweb.com/GhanaHomePage/NewsArchi](https://www.ghanaweb.com/GhanaHomePage/NewsArchive/Doctors-end-strike-go-back-to-work-on-Monday-376877)  [ve/Doctors-end-strike-go-back-to-work-on-Monday-](https://www.ghanaweb.com/GhanaHomePage/NewsArchive/Doctors-end-strike-go-back-to-work-on-Monday-376877) [376877](https://www.ghanaweb.com/GhanaHomePage/NewsArchive/Doctors-end-strike-go-back-to-work-on-Monday-376877) |
| MB9 | Doctors defy Gov't order | 20/08/2015 | Daily Guide | [https://www.ghanaweb.com/GhanaHomePage/NewsArchi](https://www.ghanaweb.com/GhanaHomePage/NewsArchive/Doctors-defy-Gov-t-order-376416) [ve/Doctors-defy-Gov-t-order-376416](https://www.ghanaweb.com/GhanaHomePage/NewsArchive/Doctors-defy-Gov-t-order-376416) |
| MB10 | Doctors deliberately slaughtering patients – Austin Gamey | 20/08/2015 | peacefmonline | [https://www.ghanaweb.com/GhanaHomePage/NewsArchi](https://www.ghanaweb.com/GhanaHomePage/NewsArchive/Doctors-deliberately-slaughtering-patients-Austin-Gamey-376484)  [ve/Doctors-deliberately-slaughtering-patients-Austin-](https://www.ghanaweb.com/GhanaHomePage/NewsArchive/Doctors-deliberately-slaughtering-patients-Austin-Gamey-376484) [Gamey-376484](https://www.ghanaweb.com/GhanaHomePage/NewsArchive/Doctors-deliberately-slaughtering-patients-Austin-Gamey-376484) |
| MB11 | Minority pleads with doctors to resume work | 20/08/2015 | kasapafmonline.com | [https://www.ghanaweb.com/GhanaHomePage/NewsArchi](https://www.ghanaweb.com/GhanaHomePage/NewsArchive/Minority-pleads-with-doctors-to-resume-work-376547)  [ve/Minority-pleads-with-doctors-to-resume-work-376547](https://www.ghanaweb.com/GhanaHomePage/NewsArchive/Minority-pleads-with-doctors-to-resume-work-376547) |
| MB12 | GMA parries Health Minister’s threats and intimidation | 19/08/2015 | tv3network.com | [https://www.ghanaweb.com/GhanaHomePage/NewsArchi](https://www.ghanaweb.com/GhanaHomePage/NewsArchive/GMA-parries-Health-Minister-s-threats-and-intimidation-376325)  [ve/GMA-parries-Health-Minister-s-threats-and-](https://www.ghanaweb.com/GhanaHomePage/NewsArchive/GMA-parries-Health-Minister-s-threats-and-intimidation-376325) [intimidation-376325](https://www.ghanaweb.com/GhanaHomePage/NewsArchive/GMA-parries-Health-Minister-s-threats-and-intimidation-376325) |
| MB13 | Minister begs doctors to go back to work | 19/08/2015 | Today Newspaper | [https://www.ghanaweb.com/GhanaHomePage/NewsArchi](https://www.ghanaweb.com/GhanaHomePage/NewsArchive/Minister-begs-doctors-to-go-back-to-work-376102) [ve/Minister-begs-doctors-to-go-back-to-work-376102](https://www.ghanaweb.com/GhanaHomePage/NewsArchive/Minister-begs-doctors-to-go-back-to-work-376102) |
| MB14 | Blame gov't for 'unfortunate' loss of lives – Dr. Ankobea | 18/08/2015 | peacefmonline | [https://www.ghanaweb.com/GhanaHomePage/NewsArchi](https://www.ghanaweb.com/GhanaHomePage/NewsArchive/Blame-gov-t-for-unfortunate-loss-of-lives-Dr-Ankobea-375912) [ve/Blame-gov-t-for-unfortunate-loss-of-lives-Dr-Ankobea-](https://www.ghanaweb.com/GhanaHomePage/NewsArchive/Blame-gov-t-for-unfortunate-loss-of-lives-Dr-Ankobea-375912) [375912](https://www.ghanaweb.com/GhanaHomePage/NewsArchive/Blame-gov-t-for-unfortunate-loss-of-lives-Dr-Ankobea-375912) |
| MB15 | Gov't must be serious in solving doctors’ strike - Kabila | 18/08/2015 | peacefmonline | [https://www.ghanaweb.com/GhanaHomePage/politics/G](https://www.ghanaweb.com/GhanaHomePage/politics/Gov-t-must-be-serious-in-solving-doctors-strike-Kabila-375919)  [ov-t-must-be-serious-in-solving-doctors-strike-Kabila-](https://www.ghanaweb.com/GhanaHomePage/politics/Gov-t-must-be-serious-in-solving-doctors-strike-Kabila-375919) [375919](https://www.ghanaweb.com/GhanaHomePage/politics/Gov-t-must-be-serious-in-solving-doctors-strike-Kabila-375919) |
| MB16 | We won’t fall for your tricks – GMA to Gov't | 18/08/2015 | kasapafmonline.com | [https://www.ghanaweb.com/GhanaHomePage/NewsArchi](https://www.ghanaweb.com/GhanaHomePage/NewsArchive/We-won-t-fall-for-your-tricks-GMA-to-Gov-t-375944) [ve/We-won-t-fall-for-your-tricks-GMA-to-Gov-t-375944](https://www.ghanaweb.com/GhanaHomePage/NewsArchive/We-won-t-fall-for-your-tricks-GMA-to-Gov-t-375944) |
| MB17 | Doctors’ strike: 500 dead in 17 days | 17/08/2015 | The Finder | [https://www.ghanaweb.com/GhanaHomePage/NewsArchi](https://www.ghanaweb.com/GhanaHomePage/NewsArchive/Doctors-strike-500-dead-in-17-days-375648) [ve/Doctors-strike-500-dead-in-17-days-375648](https://www.ghanaweb.com/GhanaHomePage/NewsArchive/Doctors-strike-500-dead-in-17-days-375648) |
| MB18 | Negotiations: No invite from govt to GMA | 17/08/2015 | The Finder | [https://www.ghanaweb.com/GhanaHomePage/NewsArchi](https://www.ghanaweb.com/GhanaHomePage/NewsArchive/Negotiations-No-invite-from-govt-to-GMA-375651) [ve/Negotiations-No-invite-from-govt-to-GMA-375651](https://www.ghanaweb.com/GhanaHomePage/NewsArchive/Negotiations-No-invite-from-govt-to-GMA-375651) |
| MB19 | Doctors strike: Mad rush for native doctors | 17/08/2015 | Daily Heritage | [https://www.ghanaweb.com/GhanaHomePage/health/Do](https://www.ghanaweb.com/GhanaHomePage/health/Doctors-strike-Mad-rush-for-native-doctors-375725)  [ctors-strike-Mad-rush-for-native-doctors-375725](https://www.ghanaweb.com/GhanaHomePage/health/Doctors-strike-Mad-rush-for-native-doctors-375725) |
| MB20 | Jawula slams doctors over stance on conditions of service | 17/08/2015 | tv3network.com | [https://www.ghanaweb.com/GhanaHomePage/NewsArchi](https://www.ghanaweb.com/GhanaHomePage/NewsArchive/Jawula-slams-doctors-over-stance-on-conditions-of-service-375755)  [ve/Jawula-slams-doctors-over-stance-on-conditions-of-](https://www.ghanaweb.com/GhanaHomePage/NewsArchive/Jawula-slams-doctors-over-stance-on-conditions-of-service-375755) [service-375755](https://www.ghanaweb.com/GhanaHomePage/NewsArchive/Jawula-slams-doctors-over-stance-on-conditions-of-service-375755) |
| MB21 | Doctors' front cracks over strike | 17/08/2015 | Public Agenda | [https://www.ghanaweb.com/GhanaHomePage/NewsArchi](https://www.ghanaweb.com/GhanaHomePage/NewsArchive/Doctors-front-cracks-over-strike-375760) [ve/Doctors-front-cracks-over-strike-375760](https://www.ghanaweb.com/GhanaHomePage/NewsArchive/Doctors-front-cracks-over-strike-375760) |
| MB22 | Doctors likely to call off strike, union says | 14/08/2015 | Reuters | [https://www.ghanaweb.com/GhanaHomePage/NewsArchi](https://www.ghanaweb.com/GhanaHomePage/NewsArchive/Doctors-likely-to-call-off-strike-union-says-375209) [ve/Doctors-likely-to-call-off-strike-union-says-375209](https://www.ghanaweb.com/GhanaHomePage/NewsArchive/Doctors-likely-to-call-off-strike-union-says-375209) |

| MB23 | Doctors' Strike: Hearing of suit against GMA adjourned | 13/08/2015 | tv3network.com | [https://www.ghanaweb.com/GhanaHomePage/NewsArchi](https://www.ghanaweb.com/GhanaHomePage/NewsArchive/Doctors-Strike-Hearing-of-suit-against-GMA-adjourned-374989) [ve/Doctors-Strike-Hearing-of-suit-against-GMA-adjourned-](https://www.ghanaweb.com/GhanaHomePage/NewsArchive/Doctors-Strike-Hearing-of-suit-against-GMA-adjourned-374989)  [374989](https://www.ghanaweb.com/GhanaHomePage/NewsArchive/Doctors-Strike-Hearing-of-suit-against-GMA-adjourned-374989) |
| --- | --- | --- | --- | --- |
| MB24 | Negotiating with a “dead goat” is difficult – Sir John | 12/08/2015 | peacefmonline | [https://www.ghanaweb.com/GhanaHomePage/NewsArchi](https://www.ghanaweb.com/GhanaHomePage/NewsArchive/Negotiating-with-a-dead-goat-is-difficult-Sir-John-374618)  [ve/Negotiating-with-a-dead-goat-is-difficult-Sir-John-](https://www.ghanaweb.com/GhanaHomePage/NewsArchive/Negotiating-with-a-dead-goat-is-difficult-Sir-John-374618) [374618](https://www.ghanaweb.com/GhanaHomePage/NewsArchive/Negotiating-with-a-dead-goat-is-difficult-Sir-John-374618) |
| MB25 | Withdrawal of doctors’ salary on point – Mahama | 12/08/2015 | kasapafmonline.com | [https://www.ghanaweb.com/GhanaHomePage/NewsArchi](https://www.ghanaweb.com/GhanaHomePage/NewsArchive/Withdrawal-of-doctors-salary-on-point-Mahama-374700)  [ve/Withdrawal-of-doctors-salary-on-point-Mahama-](https://www.ghanaweb.com/GhanaHomePage/NewsArchive/Withdrawal-of-doctors-salary-on-point-Mahama-374700) [374700](https://www.ghanaweb.com/GhanaHomePage/NewsArchive/Withdrawal-of-doctors-salary-on-point-Mahama-374700) |
| MB26 | Prof. Delle appeals to doctors to return to work | 11/08/2015 | Today Newspaper | [https://www.ghanaweb.com/GhanaHomePage/NewsArchi](https://www.ghanaweb.com/GhanaHomePage/NewsArchive/Prof-Delle-appeals-to-doctors-to-return-to-work-374301) [ve/Prof-Delle-appeals-to-doctors-to-return-to-work-](https://www.ghanaweb.com/GhanaHomePage/NewsArchive/Prof-Delle-appeals-to-doctors-to-return-to-work-374301)  [374301](https://www.ghanaweb.com/GhanaHomePage/NewsArchive/Prof-Delle-appeals-to-doctors-to-return-to-work-374301) |
| MB27 | Negotiate with doctors — PPP advises gov’t | 11/08/2015 | Today Newspaper | [https://www.ghanaweb.com/GhanaHomePage/NewsArchi](https://www.ghanaweb.com/GhanaHomePage/NewsArchive/Negotiate-with-doctors-PPP-advises-gov-t-374303)  [ve/Negotiate-with-doctors-PPP-advises-gov-t-374303](https://www.ghanaweb.com/GhanaHomePage/NewsArchive/Negotiate-with-doctors-PPP-advises-gov-t-374303) |
| MB28 | Strike: Doctors study gov’t counter-proposals | 11/08/2015 | tv3network.com | [https://www.ghanaweb.com/GhanaHomePage/NewsArchi](https://www.ghanaweb.com/GhanaHomePage/NewsArchive/Strike-Doctors-study-gov-t-counter-proposals-374310)  [ve/Strike-Doctors-study-gov-t-counter-proposals-374310](https://www.ghanaweb.com/GhanaHomePage/NewsArchive/Strike-Doctors-study-gov-t-counter-proposals-374310) |
| MB29 | Doctors don't strike beyond 24hrs in civilised societies | 11/08/2015 | atinkafmonline | [https://www.ghanaweb.com/GhanaHomePage/NewsArchi](https://www.ghanaweb.com/GhanaHomePage/NewsArchive/Doctors-don-t-strike-beyond-24hrs-in-civilised-societies-374389)  [ve/Doctors-don-t-strike-beyond-24hrs-in-civilised-](https://www.ghanaweb.com/GhanaHomePage/NewsArchive/Doctors-don-t-strike-beyond-24hrs-in-civilised-societies-374389) [societies-374389](https://www.ghanaweb.com/GhanaHomePage/NewsArchive/Doctors-don-t-strike-beyond-24hrs-in-civilised-societies-374389) |
| MB30 | GMA to Gamey: 'You are part of our problem' | 11/08/2015 | kasapafmonline.com | [https://www.ghanaweb.com/GhanaHomePage/NewsArchi](https://www.ghanaweb.com/GhanaHomePage/NewsArchive/GMA-to-Gamey-You-are-part-of-our-problem-374399) [ve/GMA-to-Gamey-You-are-part-of-our-problem-374399](https://www.ghanaweb.com/GhanaHomePage/NewsArchive/GMA-to-Gamey-You-are-part-of-our-problem-374399) |
| MB31 | We plan embarking on an indefinite strike - GMA Boss | 11/08/2015 | peacefmonline | [https://www.ghanaweb.com/GhanaHomePage/NewsArchi](https://www.ghanaweb.com/GhanaHomePage/NewsArchive/We-plan-embarking-on-an-indefinite-strike-GMA-Boss-374428) [ve/We-plan-embarking-on-an-indefinite-strike-GMA-Boss-](https://www.ghanaweb.com/GhanaHomePage/NewsArchive/We-plan-embarking-on-an-indefinite-strike-GMA-Boss-374428) [374428](https://www.ghanaweb.com/GhanaHomePage/NewsArchive/We-plan-embarking-on-an-indefinite-strike-GMA-Boss-374428) |
| MB32 | Doctors to announce next line of action Wednesday | 11/08/2015 | kasapafmonline.com | [https://www.ghanaweb.com/GhanaHomePage/NewsArchi](https://www.ghanaweb.com/GhanaHomePage/NewsArchive/Doctors-to-announce-next-line-of-action-Wednesday-374509)  [ve/Doctors-to-announce-next-line-of-action-Wednesday-](https://www.ghanaweb.com/GhanaHomePage/NewsArchive/Doctors-to-announce-next-line-of-action-Wednesday-374509) [374509](https://www.ghanaweb.com/GhanaHomePage/NewsArchive/Doctors-to-announce-next-line-of-action-Wednesday-374509) |
| MB33 | Catholic Church adopting slave labour in Ghana – ex- Minister | 11/08/2015 | kasapafmonline.com | [https://www.ghanaweb.com/GhanaHomePage/NewsArchi](https://www.ghanaweb.com/GhanaHomePage/NewsArchive/Catholic-Church-adopting-slave-labour-in-Ghana-ex-Minister-374511)  [ve/Catholic-Church-adopting-slave-labour-in-Ghana-ex-](https://www.ghanaweb.com/GhanaHomePage/NewsArchive/Catholic-Church-adopting-slave-labour-in-Ghana-ex-Minister-374511) [Minister-374511](https://www.ghanaweb.com/GhanaHomePage/NewsArchive/Catholic-Church-adopting-slave-labour-in-Ghana-ex-Minister-374511) |
| MB34 | Doctors’ strike:Diabetic patient pleads for quick end | 11/08/2015 | Ghana News Agency | [https://www.ghanaweb.com/GhanaHomePage/health/Do](https://www.ghanaweb.com/GhanaHomePage/health/Doctors-strike-Diabetic-patient-pleads-for-quick-end-374512) [ctors-strike-Diabetic-patient-pleads-for-quick-end-374512](https://www.ghanaweb.com/GhanaHomePage/health/Doctors-strike-Diabetic-patient-pleads-for-quick-end-374512) |
| MB35 | Dr Tetteh urges striking doctors to go back to work | 10/08/2015 | Ghana News Agency | [https://www.ghanaweb.com/GhanaHomePage/NewsArchi](https://www.ghanaweb.com/GhanaHomePage/NewsArchive/Dr-Tetteh-urges-striking-doctors-to-go-back-to-work-374159)  [ve/Dr-Tetteh-urges-striking-doctors-to-go-back-to-work-](https://www.ghanaweb.com/GhanaHomePage/NewsArchive/Dr-Tetteh-urges-striking-doctors-to-go-back-to-work-374159) [374159](https://www.ghanaweb.com/GhanaHomePage/NewsArchive/Dr-Tetteh-urges-striking-doctors-to-go-back-to-work-374159) |
| MB36 | Mahama owes striking doctors no apology - Kwakye- Ofosu | 10/08/2015 | peacefmonline | [https://www.ghanaweb.com/GhanaHomePage/NewsArchi](https://www.ghanaweb.com/GhanaHomePage/NewsArchive/Mahama-owes-striking-doctors-no-apology-Kwakye-Ofosu-374191)  [ve/Mahama-owes-striking-doctors-no-apology-Kwakye-](https://www.ghanaweb.com/GhanaHomePage/NewsArchive/Mahama-owes-striking-doctors-no-apology-Kwakye-Ofosu-374191) [Ofosu-374191](https://www.ghanaweb.com/GhanaHomePage/NewsArchive/Mahama-owes-striking-doctors-no-apology-Kwakye-Ofosu-374191) |
| MB37 | Gov’t hindering progress of negotiations - GMA | 10/08/2015 | peacefmonline | [https://www.ghanaweb.com/GhanaHomePage/NewsArchi](https://www.ghanaweb.com/GhanaHomePage/NewsArchive/Gov-t-hindering-progress-of-negotiations-GMA-374197) [ve/Gov-t-hindering-progress-of-negotiations-GMA-374197](https://www.ghanaweb.com/GhanaHomePage/NewsArchive/Gov-t-hindering-progress-of-negotiations-GMA-374197) |
| MB38 | We’ll insult anyone who insults us – GMA | 10/08/2015 | kasapafmonline.com | [https://www.ghanaweb.com/GhanaHomePage/NewsArchi](https://www.ghanaweb.com/GhanaHomePage/NewsArchive/We-ll-insult-anyone-who-insults-us-GMA-374234) [ve/We-ll-insult-anyone-who-insults-us-GMA-374234](https://www.ghanaweb.com/GhanaHomePage/NewsArchive/We-ll-insult-anyone-who-insults-us-GMA-374234) |

| MB39 | Go to NLC or resign now - Gamey to striking doctors | 10/08/2015 | kasapafmonline.com | [https://www.ghanaweb.com/GhanaHomePage/NewsArchi](https://www.ghanaweb.com/GhanaHomePage/NewsArchive/Go-to-NLC-or-resign-now-Gamey-to-striking-doctors-374235) [ve/Go-to-NLC-or-resign-now-Gamey-to-striking-doctors-](https://www.ghanaweb.com/GhanaHomePage/NewsArchive/Go-to-NLC-or-resign-now-Gamey-to-striking-doctors-374235)  [374235](https://www.ghanaweb.com/GhanaHomePage/NewsArchive/Go-to-NLC-or-resign-now-Gamey-to-striking-doctors-374235) |
| --- | --- | --- | --- | --- |
| MB40 | Sack Nunoo-Mensah; he doesn't deserve his salary - Ken Kuranchie | 10/08/2015 | kasapafmonline.com | [https://www.ghanaweb.com/GhanaHomePage/politics/Sa](https://www.ghanaweb.com/GhanaHomePage/politics/Sack-Nunoo-Mensah-he-doesn-t-deserve-his-salary-Ken-Kuranchie-374239)  [ck-Nunoo-Mensah-he-doesn-t-deserve-his-salary-Ken-](https://www.ghanaweb.com/GhanaHomePage/politics/Sack-Nunoo-Mensah-he-doesn-t-deserve-his-salary-Ken-Kuranchie-374239) [Kuranchie-374239](https://www.ghanaweb.com/GhanaHomePage/politics/Sack-Nunoo-Mensah-he-doesn-t-deserve-his-salary-Ken-Kuranchie-374239) |
| MB41 | Strikes: Christian Council appeals to churches to intervene | 09/08/2015 | Ghana News Agency | [https://www.ghanaweb.com/GhanaHomePage/NewsArchi](https://www.ghanaweb.com/GhanaHomePage/NewsArchive/Strikes-Christian-Council-appeals-to-churches-to-intervene-373952)  [ve/Strikes-Christian-Council-appeals-to-churches-to-](https://www.ghanaweb.com/GhanaHomePage/NewsArchive/Strikes-Christian-Council-appeals-to-churches-to-intervene-373952) [intervene-373952](https://www.ghanaweb.com/GhanaHomePage/NewsArchive/Strikes-Christian-Council-appeals-to-churches-to-intervene-373952) |
| MB42 | Pain & anguish as Doctors dump hospitals | 08/08/2015 | dailyguideghana.com | [https://www.ghanaweb.com/GhanaHomePage/NewsArchi](https://www.ghanaweb.com/GhanaHomePage/NewsArchive/Pain-anguish-as-Doctors-dump-hospitals-373844) [ve/Pain-anguish-as-Doctors-dump-hospitals-373844](https://www.ghanaweb.com/GhanaHomePage/NewsArchive/Pain-anguish-as-Doctors-dump-hospitals-373844) |
| MB43 | ‘Gov’t not truthful to Ghanaians’ | 08/08/2015 | dailyguideghana.com | [https://www.ghanaweb.com/GhanaHomePage/NewsArchi](https://www.ghanaweb.com/GhanaHomePage/NewsArchive/Gov-t-not-truthful-to-Ghanaians-373852) [ve/Gov-t-not-truthful-to-Ghanaians-373852](https://www.ghanaweb.com/GhanaHomePage/NewsArchive/Gov-t-not-truthful-to-Ghanaians-373852) |
| MB44 | Doctors’ Strike: Out-Patient Departments empty (Photos) | 08/08/2015 | [www.ghanaweb.com](http://www.ghanaweb.com/) | [https://www.ghanaweb.com/GhanaHomePage/NewsArchi](https://www.ghanaweb.com/GhanaHomePage/NewsArchive/Doctors-Strike-Out-Patient-Departments-empty-Photos-373860)  [ve/Doctors-Strike-Out-Patient-Departments-empty-](https://www.ghanaweb.com/GhanaHomePage/NewsArchive/Doctors-Strike-Out-Patient-Departments-empty-Photos-373860) [Photos-373860](https://www.ghanaweb.com/GhanaHomePage/NewsArchive/Doctors-Strike-Out-Patient-Departments-empty-Photos-373860) |
| MB45 | Striking Doctors to meet Parliament | 08/08/2015 | tv3network.com | [https://www.ghanaweb.com/GhanaHomePage/NewsArchi](https://www.ghanaweb.com/GhanaHomePage/NewsArchive/Striking-Doctors-to-meet-Parliament-373576)  [ve/Striking-Doctors-to-meet-Parliament-373576](https://www.ghanaweb.com/GhanaHomePage/NewsArchive/Striking-Doctors-to-meet-Parliament-373576) |
| MB46 | No pressure on us – 37 Military Hospital | 07/08/2015 | tv3network.com | [https://www.ghanaweb.com/GhanaHomePage/NewsArchi](https://www.ghanaweb.com/GhanaHomePage/NewsArchive/No-pressure-on-us-37-Military-Hospital-373577) [ve/No-pressure-on-us-37-Military-Hospital-373577](https://www.ghanaweb.com/GhanaHomePage/NewsArchive/No-pressure-on-us-37-Military-Hospital-373577) |
| MB47 | NPP manipulating Doctors – Anita | 07/08/2015 | dailyguideghana.com | [https://www.ghanaweb.com/GhanaHomePage/NewsArchi](https://www.ghanaweb.com/GhanaHomePage/NewsArchive/NPP-manipulating-Doctors-Anita-373644) [ve/NPP-manipulating-Doctors-Anita-373644](https://www.ghanaweb.com/GhanaHomePage/NewsArchive/NPP-manipulating-Doctors-Anita-373644) |
| MB48 | Don’t reduce our demands to partisan politics - GMA | 07/08/2015 | peacefmonline | [https://www.ghanaweb.com/GhanaHomePage/NewsArchi](https://www.ghanaweb.com/GhanaHomePage/NewsArchive/Don-t-reduce-our-demands-to-partisan-politics-GMA-373679)  [ve/Don-t-reduce-our-demands-to-partisan-politics-GMA-](https://www.ghanaweb.com/GhanaHomePage/NewsArchive/Don-t-reduce-our-demands-to-partisan-politics-GMA-373679) [373679](https://www.ghanaweb.com/GhanaHomePage/NewsArchive/Don-t-reduce-our-demands-to-partisan-politics-GMA-373679) |
| MB49 | GHS boss appeals to doctors to end strike | 07/08/2015 | Ghanaian Times | [https://www.ghanaweb.com/GhanaHomePage/NewsArchi](https://www.ghanaweb.com/GhanaHomePage/NewsArchive/GHS-boss-appeals-to-doctors-to-end-strike-373707) [ve/GHS-boss-appeals-to-doctors-to-end-strike-373707](https://www.ghanaweb.com/GhanaHomePage/NewsArchive/GHS-boss-appeals-to-doctors-to-end-strike-373707) |
| MB50 | GMA strike is 'crude and wrong' - Kofi Adams | 06/08/2015 | peacefmonline | [https://www.ghanaweb.com/GhanaHomePage/NewsArchi](https://www.ghanaweb.com/GhanaHomePage/NewsArchive/GMA-strike-is-crude-and-wrong-Kofi-Adams-373447) [ve/GMA-strike-is-crude-and-wrong-Kofi-Adams-373447](https://www.ghanaweb.com/GhanaHomePage/NewsArchive/GMA-strike-is-crude-and-wrong-Kofi-Adams-373447) |
| MB51 | Mahama’s Gov't is lazy - Nana Akomea | 06/08/2015 | peacefmonline |  |
| MB52 | 'Workers shouldn’t be allowed to strike for more than 72hrs' | 06/08/2015 | peacefmonline | [https://www.ghanaweb.com/GhanaHomePage/NewsArchi](https://www.ghanaweb.com/GhanaHomePage/NewsArchive/Workers-shouldn-t-be-allowed-to-strike-for-more-than-72hrs-373458) [ve/Workers-shouldn-t-be-allowed-to-strike-for-more-](https://www.ghanaweb.com/GhanaHomePage/NewsArchive/Workers-shouldn-t-be-allowed-to-strike-for-more-than-72hrs-373458)  [than-72hrs-373458](https://www.ghanaweb.com/GhanaHomePage/NewsArchive/Workers-shouldn-t-be-allowed-to-strike-for-more-than-72hrs-373458) |
| MB53 | Mahama’s comment on doctors strike, a wrong move – Dr Amoako Baah | 06/08/2015 | kasapafmonline.com | [https://www.ghanaweb.com/GhanaHomePage/NewsArchi](https://www.ghanaweb.com/GhanaHomePage/NewsArchive/Mahama-s-comment-on-doctors-strike-a-wrong-move-Dr-Amoako-Baah-373460)  [ve/Mahama-s-comment-on-doctors-strike-a-wrong-move-](https://www.ghanaweb.com/GhanaHomePage/NewsArchive/Mahama-s-comment-on-doctors-strike-a-wrong-move-Dr-Amoako-Baah-373460) [Dr-Amoako-Baah-373460](https://www.ghanaweb.com/GhanaHomePage/NewsArchive/Mahama-s-comment-on-doctors-strike-a-wrong-move-Dr-Amoako-Baah-373460) |
| MB54 | No cash for striking doctors | 05/08/2015 | Daily Guide | [https://www.ghanaweb.com/GhanaHomePage/NewsArchi](https://www.ghanaweb.com/GhanaHomePage/NewsArchive/No-cash-for-striking-doctors-373261) [ve/No-cash-for-striking-doctors-373261](https://www.ghanaweb.com/GhanaHomePage/NewsArchive/No-cash-for-striking-doctors-373261) |
| MB55 | No Gov't can meet GMAs demands - Kennedy Agyapong | 05/08/2015 | Daily Post | [https://www.ghanaweb.com/GhanaHomePage/NewsArchi](https://www.ghanaweb.com/GhanaHomePage/NewsArchive/No-Gov-t-can-meet-GMAs-demands-Kennedy-Agyapong-373277)  [ve/No-Gov-t-can-meet-GMAs-demands-Kennedy-](https://www.ghanaweb.com/GhanaHomePage/NewsArchive/No-Gov-t-can-meet-GMAs-demands-Kennedy-Agyapong-373277) [Agyapong-373277](https://www.ghanaweb.com/GhanaHomePage/NewsArchive/No-Gov-t-can-meet-GMAs-demands-Kennedy-Agyapong-373277) |

| MB56 | Striking doctors adopted bad negotiation tactics – Ben Ephson | 05/08/2015 | kasapafmonline.com | [https://www.ghanaweb.com/GhanaHomePage/NewsArchi](https://www.ghanaweb.com/GhanaHomePage/NewsArchive/Striking-doctors-adopted-bad-negotiation-tactics-Ben-Ephson-373279) [ve/Striking-doctors-adopted-bad-negotiation-tactics-Ben-](https://www.ghanaweb.com/GhanaHomePage/NewsArchive/Striking-doctors-adopted-bad-negotiation-tactics-Ben-Ephson-373279)  [Ephson-373279](https://www.ghanaweb.com/GhanaHomePage/NewsArchive/Striking-doctors-adopted-bad-negotiation-tactics-Ben-Ephson-373279) |
| --- | --- | --- | --- | --- |
| MB57 | Mahama to Doctors: 'I’m determined, no matter the political cost' | 05/08/2015 | tv3network.com | [https://www.ghanaweb.com/GhanaHomePage/NewsArchi](https://www.ghanaweb.com/GhanaHomePage/NewsArchive/Mahama-to-Doctors-I-m-determined-no-matter-the-political-cost-373286)  [ve/Mahama-to-Doctors-I-m-determined-no-matter-the-](https://www.ghanaweb.com/GhanaHomePage/NewsArchive/Mahama-to-Doctors-I-m-determined-no-matter-the-political-cost-373286) [political-cost-373286](https://www.ghanaweb.com/GhanaHomePage/NewsArchive/Mahama-to-Doctors-I-m-determined-no-matter-the-political-cost-373286) |
| MB58 | NPP to doctors: 'Consider the poor; call off strike' | 05/08/2015 | kasapafmonline.com | [https://www.ghanaweb.com/GhanaHomePage/NewsArchi](https://www.ghanaweb.com/GhanaHomePage/NewsArchive/NPP-to-doctors-Consider-the-poor-call-off-strike-373178)  [ve/NPP-to-doctors-Consider-the-poor-call-off-strike-](https://www.ghanaweb.com/GhanaHomePage/NewsArchive/NPP-to-doctors-Consider-the-poor-call-off-strike-373178) [373178](https://www.ghanaweb.com/GhanaHomePage/NewsArchive/NPP-to-doctors-Consider-the-poor-call-off-strike-373178) |
| MB59 | NDC slams GMA's Dr. Serebour | 04/08/2015 | kasapafmonline.com | [https://www.ghanaweb.com/GhanaHomePage/NewsArchi](https://www.ghanaweb.com/GhanaHomePage/NewsArchive/NDC-slams-GMA-s-Dr-Serebour-372995) [ve/NDC-slams-GMA-s-Dr-Serebour-372995](https://www.ghanaweb.com/GhanaHomePage/NewsArchive/NDC-slams-GMA-s-Dr-Serebour-372995) |
| MB60 | Dissenting doctors tell Serebour to resign alone | 04/08/2015 | The Republic | [https://www.ghanaweb.com/GhanaHomePage/NewsArchi](https://www.ghanaweb.com/GhanaHomePage/NewsArchive/Dissenting-doctors-tell-Serebour-to-resign-alone-373001)  [ve/Dissenting-doctors-tell-Serebour-to-resign-alone-](https://www.ghanaweb.com/GhanaHomePage/NewsArchive/Dissenting-doctors-tell-Serebour-to-resign-alone-373001) [373001](https://www.ghanaweb.com/GhanaHomePage/NewsArchive/Dissenting-doctors-tell-Serebour-to-resign-alone-373001) |
| MB61 | The worst is yet to come - Doctors warn | 12/02/2013 | Ghana News Agency | [https://www.ghanaweb.com/GhanaHomePage/NewsArchi](https://www.ghanaweb.com/GhanaHomePage/NewsArchive/The-worst-is-yet-to-come-Doctors-warn-264790)  [ve/The-worst-is-yet-to-come-Doctors-warn-264790](https://www.ghanaweb.com/GhanaHomePage/NewsArchive/The-worst-is-yet-to-come-Doctors-warn-264790) |
| MB62 | GMA strike is wrong and "uncivilized" - Austin Gamey | 12/02/2013 | CitiFM | [https://www.ghanaweb.com/GhanaHomePage/NewsArchi](https://www.ghanaweb.com/GhanaHomePage/NewsArchive/GMA-strike-is-wrong-and-uncivilized-Austin-Gamey-264791)  [ve/GMA-strike-is-wrong-and-uncivilized-Austin-Gamey-](https://www.ghanaweb.com/GhanaHomePage/NewsArchive/GMA-strike-is-wrong-and-uncivilized-Austin-Gamey-264791) [264791](https://www.ghanaweb.com/GhanaHomePage/NewsArchive/GMA-strike-is-wrong-and-uncivilized-Austin-Gamey-264791) |
| MB63 | Prof. Akosa slams GMA over strike | 12/02/2013 | radioxyzonline.com | [https://www.ghanaweb.com/GhanaHomePage/NewsArchi](https://www.ghanaweb.com/GhanaHomePage/NewsArchive/Prof-Akosa-slams-GMA-over-strike-264824) [ve/Prof-Akosa-slams-GMA-over-strike-264824](https://www.ghanaweb.com/GhanaHomePage/NewsArchive/Prof-Akosa-slams-GMA-over-strike-264824) |
| MB64 | GMA strike is illegal - NLC | 11/02/2013 | CitiFM | [https://www.ghanaweb.com/GhanaHomePage/NewsArchi](https://www.ghanaweb.com/GhanaHomePage/NewsArchive/GMA-strike-is-illegal-NLC-264712) [ve/GMA-strike-is-illegal-NLC-264712](https://www.ghanaweb.com/GhanaHomePage/NewsArchive/GMA-strike-is-illegal-NLC-264712) |
| MB65 | Ghana's doctors demanding better working conditions | 2015 |  | <https://www.youtube.com/watch?v=VWR9NtODQCw> |
| MB66 | Ghanaian Doctors Strike Over Conditions of Service | 08/08/2015 |  | [https://www.voanews.com/a/ghanaian-doctors-strike-](https://www.voanews.com/a/ghanaian-doctors-strike-over-conditions-of-service-/2908499.html) [over-conditions-of-service-/2908499.html](https://www.voanews.com/a/ghanaian-doctors-strike-over-conditions-of-service-/2908499.html) |
| MB67 | GHOSPA declares strike | 04/08/2015 | Pulse.com | [https://www.pulse.com.gh/ece-frontpage/another-strike-](https://www.pulse.com.gh/ece-frontpage/another-strike-ghospa-declares-strike/fyrw7kl) [ghospa-declares-strike/fyrw7kl](https://www.pulse.com.gh/ece-frontpage/another-strike-ghospa-declares-strike/fyrw7kl) |
| MB68 | Government pharmacists embark on strike across the country | 29/07/2015 | News Ghana | [https://newsghana.com.gh/government-pharmacists-](https://newsghana.com.gh/government-pharmacists-embark-on-strike-across-the-country/) [embark-on-strike-across-the-country/](https://newsghana.com.gh/government-pharmacists-embark-on-strike-across-the-country/) |
| MB69 | Pharmacists to strike Friday | 29/07/2015 | starrfmonline.com | [http://www.ghanareview.com/Restyle/index2.php?class=](http://www.ghanareview.com/Restyle/index2.php?class=News&date=2015-07-29&id=64570) [News&date=2015-07-29&id=64570](http://www.ghanareview.com/Restyle/index2.php?class=News&date=2015-07-29&id=64570) |
| MB70 | Press statement delivered by the Minister of Health,  Hon. Alex Sebgefia on the ongoing illegal strike action by the GMA on 18th August | 18/06/2015 | Joy News | [https://www.facebook.com/JoyNewsOnTV/posts/press-](https://www.facebook.com/JoyNewsOnTV/posts/press-statement-delivered-by-the-minister-for-health-hon-alex-segbefia-on-the-on/943787905693116/)  [statement-delivered-by-the-minister-for-health-hon-alex-](https://www.facebook.com/JoyNewsOnTV/posts/press-statement-delivered-by-the-minister-for-health-hon-alex-segbefia-on-the-on/943787905693116/) [segbefia-on-the-on/943787905693116/](https://www.facebook.com/JoyNewsOnTV/posts/press-statement-delivered-by-the-minister-for-health-hon-alex-segbefia-on-the-on/943787905693116/) |
| MB71 | Ghana: Government issues directives to minimize effects of the GMA strike | 18/08/2015 | all Africa | <https://allafrica.com/stories/201508191000.html> |
| MB72 | Christian health facilities struggle to deal with increased patient numbers | 10/08/2015 | Joy Online | [https://www.myjoyonline.com/christian-health-facilities-](https://www.myjoyonline.com/christian-health-facilities-struggle-to-deal-with-increased-patient-numbers/) [struggle-to-deal-with-increased-patient-numbers/](https://www.myjoyonline.com/christian-health-facilities-struggle-to-deal-with-increased-patient-numbers/) |
| MB73 | Sacked doctors are not being punished- CHAG | 11/08/2015 | Pu;se.com | [https://www.pulse.com.gh/ece-frontpage/sacked-doctors-](https://www.pulse.com.gh/ece-frontpage/sacked-doctors-sacked-doctors-are-not-being-punished-chag/86mxxlv) [sacked-doctors-are-not-being-punished-chag/86mxxlv](https://www.pulse.com.gh/ece-frontpage/sacked-doctors-sacked-doctors-are-not-being-punished-chag/86mxxlv) |

| MB74 | Come, let’s save lives; 37 Hospital invites 148 doctors who voted against strike | 15/08/2015 | Graphic Online | [https://www.graphic.com.gh/news/general-news/come-](https://www.graphic.com.gh/news/general-news/come-let-s-save-lives-37-hospital-invites-148-doctors-who-voted-against-strike.html) [let-s-save-lives-37-hospital-invites-148-doctors-who-](https://www.graphic.com.gh/news/general-news/come-let-s-save-lives-37-hospital-invites-148-doctors-who-voted-against-strike.html)  [voted-against-strike.html](https://www.graphic.com.gh/news/general-news/come-let-s-save-lives-37-hospital-invites-148-doctors-who-voted-against-strike.html) |
| --- | --- | --- | --- | --- |
| MB75 | Doctors strike: Ministry releases list of operational health facilities | 14/08/2015 | Graphic Online | [https://www.graphic.com.gh/news/general-news/doctors-](https://www.graphic.com.gh/news/general-news/doctors-strike-ministry-releases-list-of-operational-health-facilities.html)  [strike-ministry-releases-list-of-operational-health-](https://www.graphic.com.gh/news/general-news/doctors-strike-ministry-releases-list-of-operational-health-facilities.html) [facilities.html](https://www.graphic.com.gh/news/general-news/doctors-strike-ministry-releases-list-of-operational-health-facilities.html) |
| MB76 | Korle Bu Cardio Centre overstretched due to doctors strike | 13/08/2015 | Graphic Online | [https://www.graphic.com.gh/news/general-news/korle-](https://www.graphic.com.gh/news/general-news/korle-bu-cardio-centre-overstretched-due-to-doctors-strike.html) [bu-cardio-centre-overstretched-due-to-doctors-strike.html](https://www.graphic.com.gh/news/general-news/korle-bu-cardio-centre-overstretched-due-to-doctors-strike.html) |
| MB77 | Ghana to Bring Cuban Doctors to Mitigate Medical Strike | 19/08/2015 | Al Jazeera | <https://allafrica.com/stories/201508201287.html> |
| MB78 | Catholic Health Services threatens legal action against NHIA | 15/10/2015 | Graphic Online | [https://www.graphic.com.gh/news/general-](https://www.graphic.com.gh/news/general-news/catholic-health-services-threatens-legal-action-against-nhia.html) [news/catholic-health-services-threatens-legal-action-](https://www.graphic.com.gh/news/general-news/catholic-health-services-threatens-legal-action-against-nhia.html)  [against-nhia.html](https://www.graphic.com.gh/news/general-news/catholic-health-services-threatens-legal-action-against-nhia.html) |
| MB79 | Private hospitals reject NHIS cards | 25/08/2015 | Daily Guide | [https://www.ghanaweb.com/GhanaHomePage/NewsArchi](https://www.ghanaweb.com/GhanaHomePage/NewsArchive/Private-hospitals-reject-NHIS-cards-373150)  [ve/Private-hospitals-reject-NHIS-cards-373150](https://www.ghanaweb.com/GhanaHomePage/NewsArchive/Private-hospitals-reject-NHIS-cards-373150) |
| MC1 | Pharmacists in govt hospitals begin indefinite strike | 09/06/2016 | Graphic Online | [https://www.graphic.com.gh/news/general-](https://www.graphic.com.gh/news/general-news/pharmacists-in-govt-hospitals-begin-indefinite-strike.html) [news/pharmacists-in-govt-hospitals-begin-indefinite-](https://www.graphic.com.gh/news/general-news/pharmacists-in-govt-hospitals-begin-indefinite-strike.html) [strike.html](https://www.graphic.com.gh/news/general-news/pharmacists-in-govt-hospitals-begin-indefinite-strike.html) |
| MC2 | GHOSPA to consult members on strike over market premium | 11/10/2016 | Graphic Online | [https://www.graphic.com.gh/news/general-news/ghospa-](https://www.graphic.com.gh/news/general-news/ghospa-to-consult-members-on-strike-over-market-premium.html) [to-consult-members-on-strike-over-market-premium.html](https://www.graphic.com.gh/news/general-news/ghospa-to-consult-members-on-strike-over-market-premium.html) |
| MC3 | We will call of the strike if government shows commitment- GHOSPA | 13/09/2016 | Pulse.com.gh | [https://www.pulse.com.gh/news/local/ghospa-strike-we-](https://www.pulse.com.gh/news/local/ghospa-strike-we-will-call-off-strike-if-government-shows-commitment-ghospa/1khrvc6) [will-call-off-strike-if-government-shows-commitment-](https://www.pulse.com.gh/news/local/ghospa-strike-we-will-call-off-strike-if-government-shows-commitment-ghospa/1khrvc6) [ghospa/1khrvc6](https://www.pulse.com.gh/news/local/ghospa-strike-we-will-call-off-strike-if-government-shows-commitment-ghospa/1khrvc6) |
| MC4 | We are ready to face NLC in court- stiking pharmacists | 13/09/2016 | Pulse.com.gh | [https://www.pulse.com.gh/ece-frontpage/ghospa-strike-](https://www.pulse.com.gh/ece-frontpage/ghospa-strike-we-are-ready-to-face-nlc-in-court-striking-pharmacists/2n057h9)  [we-are-ready-to-face-nlc-in-court-striking-](https://www.pulse.com.gh/ece-frontpage/ghospa-strike-we-are-ready-to-face-nlc-in-court-striking-pharmacists/2n057h9) [pharmacists/2n057h9](https://www.pulse.com.gh/ece-frontpage/ghospa-strike-we-are-ready-to-face-nlc-in-court-striking-pharmacists/2n057h9) |
| MC5 | GHOSPA strike bad? Pharmacy Chamber | 14/09/2016 | Peace FM | [https://www.peacefmonline.com/pages/local/news/2016](https://www.peacefmonline.com/pages/local/news/201609/292029.php) [09/292029.php](https://www.peacefmonline.com/pages/local/news/201609/292029.php) |
| MC6 | Government and hospital pharmacists to strike September 5 | 02/09/2016 | Ghana Web | [https://www.ghanaweb.com/GhanaHomePage/NewsArchi](https://www.ghanaweb.com/GhanaHomePage/NewsArchive/Government-hospital-pharmacists-to-strike-September-5-466897) [ve/Government-hospital-pharmacists-to-strike-](https://www.ghanaweb.com/GhanaHomePage/NewsArchive/Government-hospital-pharmacists-to-strike-September-5-466897)  [September-5-466897](https://www.ghanaweb.com/GhanaHomePage/NewsArchive/Government-hospital-pharmacists-to-strike-September-5-466897) |
| MC7 | Pharmacy shop attendants record high sales over GHOSPA strike | 08/09/3016 | TV43 | <https://www.youtube.com/watch?v=GqxSpmbEUfU> |
| MC8 | We can'r pay striking pharmacists | 09/09/2016 | Daily Guide | [https://www.modernghana.com/news/718444/we-cant-](https://www.modernghana.com/news/718444/we-cant-pay-striking-pharmacists.html) [pay-striking-pharmacists.html](https://www.modernghana.com/news/718444/we-cant-pay-striking-pharmacists.html) |
| MC9 | Govt pharmacists lay down tools | 05/09/2016 | Ghana Review International | [http://www.ghanareview.com/Restyle/index2.php?class=a](http://www.ghanareview.com/Restyle/index2.php?class=all&date=2016-09-05&id=66633) [ll&date=2016-09-05&id=66633](http://www.ghanareview.com/Restyle/index2.php?class=all&date=2016-09-05&id=66633) |
| MC10 | GHOSPA asks government for clear roadmap | 14/09/2016 | Peace FM | [https://www.peacefmonline.com/pages/local/health/201](https://www.peacefmonline.com/pages/local/health/201609/292007.php) [609/292007.php](https://www.peacefmonline.com/pages/local/health/201609/292007.php) |
| MC11 | Don't engage non-experts as pharmacists | 27/09/2016 | Ghanaian Times | [https://www.businessghana.com/site/news/General/1360](https://www.businessghana.com/site/news/General/136034/Don%E2%80%99t%20engage%20non-experts%20as%20pharmacists%20%E2%80%93%20GHOSPA) [34/Don%E2%80%99t%20engage%20non-](https://www.businessghana.com/site/news/General/136034/Don%E2%80%99t%20engage%20non-experts%20as%20pharmacists%20%E2%80%93%20GHOSPA)  [experts%20as%20pharmacists%20%E2%80%93%20GHOSP](https://www.businessghana.com/site/news/General/136034/Don%E2%80%99t%20engage%20non-experts%20as%20pharmacists%20%E2%80%93%20GHOSPA) [A](https://www.businessghana.com/site/news/General/136034/Don%E2%80%99t%20engage%20non-experts%20as%20pharmacists%20%E2%80%93%20GHOSPA) |

| MC12 | Address our concerns or face our wrath-GHOSPA threatens | 11/07/2016 | Pulse.com.gh | [https://www.pulse.com.gh/ece-frontpage/aggrieved-](https://www.pulse.com.gh/ece-frontpage/aggrieved-pharmacist-address-our-concerns-or-face-our-wrath-ghospa-threatens/3b0dvnw) [pharmacist-address-our-concerns-or-face-our-wrath-](https://www.pulse.com.gh/ece-frontpage/aggrieved-pharmacist-address-our-concerns-or-face-our-wrath-ghospa-threatens/3b0dvnw)  [ghospa-threatens/3b0dvnw](https://www.pulse.com.gh/ece-frontpage/aggrieved-pharmacist-address-our-concerns-or-face-our-wrath-ghospa-threatens/3b0dvnw) |
| --- | --- | --- | --- | --- |
| MC13 | Pharmacists Leave Hospitals Empty | 06/09/2016 | Daily Guide Network | [https://dailyguidenetwork.com/pharmacists-leave-](https://dailyguidenetwork.com/pharmacists-leave-hospital-empty/) [hospital-empty/](https://dailyguidenetwork.com/pharmacists-leave-hospital-empty/) |
| MC14 | Ghanians should forgive us - striking pharmacists | 13/09/2016 | Ghana Web | [https://www.ghanaweb.com/GhanaHomePage/NewsArchi](https://www.ghanaweb.com/GhanaHomePage/NewsArchive/Ghanaians-should-forgive-us-striking-Pharmacists-469381) [ve/Ghanaians-should-forgive-us-striking-Pharmacists-](https://www.ghanaweb.com/GhanaHomePage/NewsArchive/Ghanaians-should-forgive-us-striking-Pharmacists-469381)  [469381](https://www.ghanaweb.com/GhanaHomePage/NewsArchive/Ghanaians-should-forgive-us-striking-Pharmacists-469381) |
| MC15 | Gov´t hails CHAG's contribution to healthcare | 09/06/2016 | Ghana News Agency | [https://www.ghanaweb.com/GhanaHomePage/health/Go](https://www.ghanaweb.com/GhanaHomePage/health/Gov-t-hails-CHAG-s-contribution-to-healthcare-446069) [v-t-hails-CHAG-s-contribution-to-healthcare-446069](https://www.ghanaweb.com/GhanaHomePage/health/Gov-t-hails-CHAG-s-contribution-to-healthcare-446069) |
| MC16 | Nurses, midwives start 'red week' | 31/10/2016 | Class FM | [https://www.ghanaweb.com/GhanaHomePage/NewsArchi](https://www.ghanaweb.com/GhanaHomePage/NewsArchive/Nurses-midwives-start-red-week-482322) [ve/Nurses-midwives-start-red-week-482322](https://www.ghanaweb.com/GhanaHomePage/NewsArchive/Nurses-midwives-start-red-week-482322) |
| MC17 | NHIA in Ghc420m arrears | 16/10/2016 | The Finder | [https://www.ghanaweb.com/GhanaHomePage/health/NH](https://www.ghanaweb.com/GhanaHomePage/health/NHIA-in-Ghc420m-arrears-415883)  [IA-in-Ghc420m-arrears-415883](https://www.ghanaweb.com/GhanaHomePage/health/NHIA-in-Ghc420m-arrears-415883) |
| MC18 | Bishop Titi Offei can’t think far; I also can’t think far | 05/10/2016 | Opinion Piece | [https://www.ghanaweb.com/GhanaHomePage/features/B](https://www.ghanaweb.com/GhanaHomePage/features/Bishop-Titi-Offei-can-t-think-far-I-also-can-t-think-far-474916)  [ishop-Titi-Offei-can-t-think-far-I-also-can-t-think-far-](https://www.ghanaweb.com/GhanaHomePage/features/Bishop-Titi-Offei-can-t-think-far-I-also-can-t-think-far-474916) [474916](https://www.ghanaweb.com/GhanaHomePage/features/Bishop-Titi-Offei-can-t-think-far-I-also-can-t-think-far-474916) |
| MC19 | Extend NHIS to cover herbal medicines - Monic Star Center CEO | 26/11/2016 | The Chronicle | [https://www.ghanaweb.com/GhanaHomePage/health/Ext](https://www.ghanaweb.com/GhanaHomePage/health/Extend-NHIS-to-cover-herbal-medicines-Monic-Star-Center-CEO-488855)  [end-NHIS-to-cover-herbal-medicines-Monic-Star-Center-](https://www.ghanaweb.com/GhanaHomePage/health/Extend-NHIS-to-cover-herbal-medicines-Monic-Star-Center-CEO-488855) [CEO-488855](https://www.ghanaweb.com/GhanaHomePage/health/Extend-NHIS-to-cover-herbal-medicines-Monic-Star-Center-CEO-488855) |
| MC20 | Hospitals ignore directive to ease GHOSPA strike | 22/09/2016 | 3 News | [https://www.ghanaweb.com/GhanaHomePage/health/Ho](https://www.ghanaweb.com/GhanaHomePage/health/Hospitals-ignore-directive-to-ease-GHOSPA-strike-471497) [spitals-ignore-directive-to-ease-GHOSPA-strike-471497](https://www.ghanaweb.com/GhanaHomePage/health/Hospitals-ignore-directive-to-ease-GHOSPA-strike-471497) |
| MC21 | We are worried patients can’t access drugs - Pharmacists | 26/09/2016 | Ultimate FM | [https://www.ghanaweb.com/GhanaHomePage/health/We](https://www.ghanaweb.com/GhanaHomePage/health/We-are-worried-patients-can-t-access-drugs-Pharmacists-472512)  [-are-worried-patients-can-t-access-drugs-Pharmacists-](https://www.ghanaweb.com/GhanaHomePage/health/We-are-worried-patients-can-t-access-drugs-Pharmacists-472512) [472512](https://www.ghanaweb.com/GhanaHomePage/health/We-are-worried-patients-can-t-access-drugs-Pharmacists-472512) |
| MC22 | Committee on NHIS presents report to Health Ministry | 17/09/2016 | Ghana News Agency | [https://www.ghanaweb.com/GhanaHomePage/health/Co](https://www.ghanaweb.com/GhanaHomePage/health/Committee-on-NHIS-presents-report-to-Health-Ministry-470504) [mmittee-on-NHIS-presents-report-to-Health-Ministry-](https://www.ghanaweb.com/GhanaHomePage/health/Committee-on-NHIS-presents-report-to-Health-Ministry-470504)  [470504](https://www.ghanaweb.com/GhanaHomePage/health/Committee-on-NHIS-presents-report-to-Health-Ministry-470504) |
| MC23 | Dispense drugs to patients despite GHOSPA strike - Hospitals told | 11/09/2016 | Class FM | [https://www.ghanaweb.com/GhanaHomePage/health/Dis](https://www.ghanaweb.com/GhanaHomePage/health/Dispense-drugs-to-patients-despite-GHOSPA-strike-Hospitals-told-469045)  [pense-drugs-to-patients-despite-GHOSPA-strike-Hospitals-](https://www.ghanaweb.com/GhanaHomePage/health/Dispense-drugs-to-patients-despite-GHOSPA-strike-Hospitals-told-469045) [told-469045](https://www.ghanaweb.com/GhanaHomePage/health/Dispense-drugs-to-patients-despite-GHOSPA-strike-Hospitals-told-469045) |
| MC24 | Herbal medicine: The shortcomings of professional association | 19/08/2016 | Opinion Piece | [https://www.ghanaweb.com/GhanaHomePage/features/H](https://www.ghanaweb.com/GhanaHomePage/features/Herbal-medicine-The-shortcomings-of-professional-association-1-463751) [erbal-medicine-The-shortcomings-of-professional-](https://www.ghanaweb.com/GhanaHomePage/features/Herbal-medicine-The-shortcomings-of-professional-association-1-463751) [association-1-463751](https://www.ghanaweb.com/GhanaHomePage/features/Herbal-medicine-The-shortcomings-of-professional-association-1-463751) |
| MC25 | NHIS cards issued to hit 17m by end of 2016 | 02/07/2016 | Class FM | [https://www.ghanaweb.com/GhanaHomePage/health/NH](https://www.ghanaweb.com/GhanaHomePage/health/NHIS-cards-issued-to-hit-17m-by-end-of-2016-452252) [IS-cards-issued-to-hit-17m-by-end-of-2016-452252](https://www.ghanaweb.com/GhanaHomePage/health/NHIS-cards-issued-to-hit-17m-by-end-of-2016-452252) |
| MC26 | GNAInvest more in NHIS – NGO to gov’t | 14/03/2016 | Ghana News Agency | [https://www.ghanaweb.com/GhanaHomePage/health/Inv](https://www.ghanaweb.com/GhanaHomePage/health/Invest-more-in-NHIS-NGO-to-gov-t-423161) [est-more-in-NHIS-NGO-to-gov-t-423161](https://www.ghanaweb.com/GhanaHomePage/health/Invest-more-in-NHIS-NGO-to-gov-t-423161) |
| MC27 | NHIS: Tariffs increased by 27% | 23/02/2016 | Class FM | [https://www.ghanaweb.com/GhanaHomePage/health/NH](https://www.ghanaweb.com/GhanaHomePage/health/NHIS-Tariffs-increased-by-27-418030) [IS-Tariffs-increased-by-27-418030](https://www.ghanaweb.com/GhanaHomePage/health/NHIS-Tariffs-increased-by-27-418030) |
| MC28 | Patient surge as Ghana’s doctors strike | 21/04/2013 | The Business Recorder | <https://fp.brecorder.com/2013/04/201304211176962/> |

| MC29 | NHIA pays outstanding claims | 23/03/2013 | Ghana News Agency | [https://www.ghanaweb.com/GhanaHomePage/health/NH](https://www.ghanaweb.com/GhanaHomePage/health/NHIA-pays-outstanding-claims-268759) [IA-pays-outstanding-claims-268759](https://www.ghanaweb.com/GhanaHomePage/health/NHIA-pays-outstanding-claims-268759) |
| --- | --- | --- | --- | --- |
| MC30 | Missionary hospitals will refuse NHIS patients | 10/03/2013 | Joy Online | [https://www.ghanaweb.com/GhanaHomePage/NewsArchi](https://www.ghanaweb.com/GhanaHomePage/NewsArchive/Missionary-hospitals-will-refuse-NHIS-patients-267207) [ve/Missionary-hospitals-will-refuse-NHIS-patients-267207](https://www.ghanaweb.com/GhanaHomePage/NewsArchive/Missionary-hospitals-will-refuse-NHIS-patients-267207) |
| MC31 | GHOSPA strike: Health Ministry directs GHS, CHAG to  ensure uninterrupted pharm. services delivery | 14/09/2016 | Kasapa Fm | <https://kasapafmonline.com/?p=70910> |
| MC32 | Ghana: Pharmacists to Continue Strike After Impasse  With Govt | 08/06/2016 | all Africa | <https://allafrica.com/stories/201609091218.html> |
| MC33 | CHAG to take over service delivery – MOH – GHOne TV | 2016 | GHONE | <http://ghonetv.com/ghospa-strike-chag-take-service-delivery-moh/> |
| MD1 | Lawyers, Doctors Gear for A Showdown | 01/11/2011 | The Republic Newspaper | [https://www.ghanaweb.com/GhanaHomePage/NewsArchi](https://www.ghanaweb.com/GhanaHomePage/NewsArchive/Lawyers-Doctors-Gear-for-A-Showdown-222775) [ve/Lawyers-Doctors-Gear-for-A-Showdown-222775](https://www.ghanaweb.com/GhanaHomePage/NewsArchive/Lawyers-Doctors-Gear-for-A-Showdown-222775) |
| MD2 | Labour Commission rules on GMA and FWSC disputes | 14/11/2011 | GNA- Frank Atiase | [https://www.ghanaweb.com/GhanaHomePage/business/L](https://www.ghanaweb.com/GhanaHomePage/business/Labour-Commission-rules-on-GMA-and-FWSC-disputes-223566) [abour-Commission-rules-on-GMA-and-FWSC-disputes-](https://www.ghanaweb.com/GhanaHomePage/business/Labour-Commission-rules-on-GMA-and-FWSC-disputes-223566) [223566](https://www.ghanaweb.com/GhanaHomePage/business/Labour-Commission-rules-on-GMA-and-FWSC-disputes-223566) |
| MD3 | NLC gives ruling on Ghana Medical Association’s compulsory arbitration process | 08/11/2011 | Ghana Business News | [https://www.ghanaweb.com/GhanaHomePage/business/L](https://www.ghanaweb.com/GhanaHomePage/business/Labour-Commission-rules-on-GMA-and-FWSC-disputes-223566)  [abour-Commission-rules-on-GMA-and-FWSC-disputes-](https://www.ghanaweb.com/GhanaHomePage/business/Labour-Commission-rules-on-GMA-and-FWSC-disputes-223566) [223566](https://www.ghanaweb.com/GhanaHomePage/business/Labour-Commission-rules-on-GMA-and-FWSC-disputes-223566) |
| MD4 | PUSAG Urges Doctors To End Strike | 25/10/2011 | GNA- Lens | [https://www.ghanaweb.com/GhanaHomePage/regional/P](https://www.ghanaweb.com/GhanaHomePage/regional/PUSAG-Urges-Doctors-To-End-Strike-222339) [USAG-Urges-Doctors-To-End-Strike-222339](https://www.ghanaweb.com/GhanaHomePage/regional/PUSAG-Urges-Doctors-To-End-Strike-222339) |
| MD5 | Doctors earn a fraction of what presidential aides get | 24/10/2011 | New Crusading guide | [https://www.ghanaweb.com/GhanaHomePage/NewsArchi](https://www.ghanaweb.com/GhanaHomePage/NewsArchive/Doctors-earn-a-fraction-of-what-presidential-aides-get-222209)  [ve/Doctors-earn-a-fraction-of-what-presidential-aides-get-](https://www.ghanaweb.com/GhanaHomePage/NewsArchive/Doctors-earn-a-fraction-of-what-presidential-aides-get-222209) [222209](https://www.ghanaweb.com/GhanaHomePage/NewsArchive/Doctors-earn-a-fraction-of-what-presidential-aides-get-222209) |
| MD6 | Doctors’ strike, Kadhafi’s death highlighted in media | 23/10/2011 | Pana | [https://www.ghanaweb.com/GhanaHomePage/NewsArchi](https://www.ghanaweb.com/GhanaHomePage/NewsArchive/Doctors-strike-Kadhafi-s-death-highlighted-in-media-222177) [ve/Doctors-strike-Kadhafi-s-death-highlighted-in-media-](https://www.ghanaweb.com/GhanaHomePage/NewsArchive/Doctors-strike-Kadhafi-s-death-highlighted-in-media-222177) [222177](https://www.ghanaweb.com/GhanaHomePage/NewsArchive/Doctors-strike-Kadhafi-s-death-highlighted-in-media-222177) |
| MD7 | Bishop Appeals to GMA to call off strike | 20/10/2011 | GNA | [https://www.ghanaweb.com/GhanaHomePage/health/Bis](https://www.ghanaweb.com/GhanaHomePage/health/Bishop-Appeals-to-GMA-to-call-off-strike-222017) [hop-Appeals-to-GMA-to-call-off-strike-222017](https://www.ghanaweb.com/GhanaHomePage/health/Bishop-Appeals-to-GMA-to-call-off-strike-222017) |
| MD8 | Doctors Say Strike Still Not Over | 19/10/2011 | GNA | [https://www.ghanaweb.com/GhanaHomePage/NewsArchi](https://www.ghanaweb.com/GhanaHomePage/NewsArchive/Doctors-Say-Strike-Still-Not-Over-221972) [ve/Doctors-Say-Strike-Still-Not-Over-221972](https://www.ghanaweb.com/GhanaHomePage/NewsArchive/Doctors-Say-Strike-Still-Not-Over-221972) |
| MD9 | Coalition Of Ngos In Health Appeals To Doctors To End Strike Action | 17/10/2011 | GNA? | [https://www.ghanaweb.com/GhanaHomePage/regional/C](https://www.ghanaweb.com/GhanaHomePage/regional/Coalition-Of-Ngos-In-Health-Appeals-To-Doctors-To-End-Strike-Action-221870)  [oalition-Of-Ngos-In-Health-Appeals-To-Doctors-To-End-](https://www.ghanaweb.com/GhanaHomePage/regional/Coalition-Of-Ngos-In-Health-Appeals-To-Doctors-To-End-Strike-Action-221870) [Strike-Action-221870](https://www.ghanaweb.com/GhanaHomePage/regional/Coalition-Of-Ngos-In-Health-Appeals-To-Doctors-To-End-Strike-Action-221870) |
| MD10 | NPP man Wants Doctor's Salaries Withheld | 17/10/2011 | Daily Post | [https://www.ghanaweb.com/GhanaHomePage/NewsArchi](https://www.ghanaweb.com/GhanaHomePage/NewsArchive/NPP-man-Wants-Doctor-s-Salaries-Withheld-221865) [ve/NPP-man-Wants-Doctor-s-Salaries-Withheld-221865](https://www.ghanaweb.com/GhanaHomePage/NewsArchive/NPP-man-Wants-Doctor-s-Salaries-Withheld-221865) |
| MD11 | Christian Counncil appeals to doctors to end strike | 16/10/2011 | GNA | [https://www.ghanaweb.com/GhanaHomePage/health/Chr](https://www.ghanaweb.com/GhanaHomePage/health/Christian-Counncil-appeals-to-doctors-to-end-strike-221761) [istian-Counncil-appeals-to-doctors-to-end-strike-221761](https://www.ghanaweb.com/GhanaHomePage/health/Christian-Counncil-appeals-to-doctors-to-end-strike-221761) |
| MD12 | Doctors strike impacts on Police and 37 Military Hospitals | 12/10/2011 | GNA | [https://www.ghanaweb.com/GhanaHomePage/health/Do](https://www.ghanaweb.com/GhanaHomePage/health/Doctors-strike-impacts-on-Police-and-37-Military-Hospitals-221477)  [ctors-strike-impacts-on-Police-and-37-Military-Hospitals-](https://www.ghanaweb.com/GhanaHomePage/health/Doctors-strike-impacts-on-Police-and-37-Military-Hospitals-221477) [221477](https://www.ghanaweb.com/GhanaHomePage/health/Doctors-strike-impacts-on-Police-and-37-Military-Hospitals-221477) |
| MD13 | Forum appeals to GMA to end Strike | 12/10/2011 | GNA | [https://www.ghanaweb.com/GhanaHomePage/regional/F](https://www.ghanaweb.com/GhanaHomePage/regional/Forum-appeals-to-GMA-to-end-Strike-221475) [orum-appeals-to-GMA-to-end-Strike-221475](https://www.ghanaweb.com/GhanaHomePage/regional/Forum-appeals-to-GMA-to-end-Strike-221475) |
| MD14 | Statement by the Forum for Governance & Justice on GMA strike | 12/10/2011 | myjoyonline | [https://www.myjoyonline.com/statement-by-the-forum-](https://www.myjoyonline.com/statement-by-the-forum-for-governance-justice-on-gma-strike/) [for-governance-justice-on-gma-strike/](https://www.myjoyonline.com/statement-by-the-forum-for-governance-justice-on-gma-strike/) |

| MD15 | Mills blames NPP for doctors’ strike | 12/10/2011 | Statesman | [https://www.ghanaweb.com/GhanaHomePage/NewsArchi](https://www.ghanaweb.com/GhanaHomePage/NewsArchive/Mills-blames-NPP-for-doctors-strike-221544) [ve/Mills-blames-NPP-for-doctors-strike-221544](https://www.ghanaweb.com/GhanaHomePage/NewsArchive/Mills-blames-NPP-for-doctors-strike-221544) |
| --- | --- | --- | --- | --- |
| MD16 | President Mills announces measures to contain doctors’ strike | 12/10/2011 | Ghana Business News | [https://www.ghanabusinessnews.com/2011/10/17/presid](https://www.ghanabusinessnews.com/2011/10/17/president-mills-announces-measures-to-contain-doctors-strike/) [ent-mills-announces-measures-to-contain-doctors-strike/](https://www.ghanabusinessnews.com/2011/10/17/president-mills-announces-measures-to-contain-doctors-strike/) |
| MD17 | Gov’t to prevent doctors from going on strike in the  future – Haruna Iddrisu | 17/10/2011 | myjoyonline | [https://www.myjoyonline.com/govt-to-prevent-doctors-](https://www.myjoyonline.com/govt-to-prevent-doctors-from-going-on-strike-in-the-future-haruna-iddrisu/)  [from-going-on-strike-in-the-future-haruna-iddrisu/](https://www.myjoyonline.com/govt-to-prevent-doctors-from-going-on-strike-in-the-future-haruna-iddrisu/) |
| MD18 | Mills will meet striking doctors when necessary- Council  of State Member | 18/10/2011 | myjoyonline | [https://www.myjoyonline.com/mills-will-meet-striking-](https://www.myjoyonline.com/mills-will-meet-striking-doctors-when-necessary-council-of-state-member/)  [doctors-when-necessary-council-of-state-member/](https://www.myjoyonline.com/mills-will-meet-striking-doctors-when-necessary-council-of-state-member/) |
| MD19 | Mills blamed for prolonged doctors' strike | 26/10/2011 | Modern Ghana | [https://www.modernghana.com/news/357796/mills-](https://www.modernghana.com/news/357796/mills-blamed-for-prolonged-doctors-strike.html) [blamed-for-prolonged-doctors-strike.html](https://www.modernghana.com/news/357796/mills-blamed-for-prolonged-doctors-strike.html) |
| MD20 | Doctor says the strike action will continue | 11/10/2011 | GNA | [https://www.ghanaweb.com/GhanaHomePage/regional/D](https://www.ghanaweb.com/GhanaHomePage/regional/Doctor-says-the-strike-action-will-continue-221396) [octor-says-the-strike-action-will-continue-221396](https://www.ghanaweb.com/GhanaHomePage/regional/Doctor-says-the-strike-action-will-continue-221396) |
| MD21 | Doctors deny refusing to negotiate with FWSC | 11/10/2011 | GNA | [https://www.ghanaweb.com/GhanaHomePage/NewsArchi](https://www.ghanaweb.com/GhanaHomePage/NewsArchive/Doctors-deny-refusing-to-negotiate-with-FWSC-221386)  [ve/Doctors-deny-refusing-to-negotiate-with-FWSC-221386](https://www.ghanaweb.com/GhanaHomePage/NewsArchive/Doctors-deny-refusing-to-negotiate-with-FWSC-221386) |
| MD22 | Full text of statement by Ghana Medical Association | 10/10/2011 | GNA | [https://www.ghanaweb.com/GhanaHomePage/NewsArchi](https://www.ghanaweb.com/GhanaHomePage/NewsArchive/Full-text-of-statement-by-Ghana-Medical-Association-221521) [ve/Full-text-of-statement-by-Ghana-Medical-Association-](https://www.ghanaweb.com/GhanaHomePage/NewsArchive/Full-text-of-statement-by-Ghana-Medical-Association-221521)  [221521](https://www.ghanaweb.com/GhanaHomePage/NewsArchive/Full-text-of-statement-by-Ghana-Medical-Association-221521) |
| MD23 | KATH rejects critically injured accident victim | 11/10/2011 | GNA | [https://www.ghanaweb.com/GhanaHomePage/NewsArchi](https://www.ghanaweb.com/GhanaHomePage/NewsArchive/KATH-rejects-critically-injured-accident-victim-221565) [ve/KATH-rejects-critically-injured-accident-victim-221565](https://www.ghanaweb.com/GhanaHomePage/NewsArchive/KATH-rejects-critically-injured-accident-victim-221565) |
| MD24 | Ministry of Health ask striking doctors to resume work | 11/10/2011 | GNA | [https://www.ghanaweb.com/GhanaHomePage/NewsArchi](https://www.ghanaweb.com/GhanaHomePage/NewsArchive/Ministry-of-Health-ask-striking-doctors-to-resume-work-221352)  [ve/Ministry-of-Health-ask-striking-doctors-to-resume-](https://www.ghanaweb.com/GhanaHomePage/NewsArchive/Ministry-of-Health-ask-striking-doctors-to-resume-work-221352) [work-221352](https://www.ghanaweb.com/GhanaHomePage/NewsArchive/Ministry-of-Health-ask-striking-doctors-to-resume-work-221352) |
| MD25 | Patients in Ho laud doctors for keeping faith with them | 11/10/2011 | GNA | [https://www.ghanaweb.com/GhanaHomePage/health/Pat](https://www.ghanaweb.com/GhanaHomePage/health/Patients-in-Ho-laud-doctors-for-keeping-faith-with-them-221387)  [ients-in-Ho-laud-doctors-for-keeping-faith-with-them-](https://www.ghanaweb.com/GhanaHomePage/health/Patients-in-Ho-laud-doctors-for-keeping-faith-with-them-221387) [221387](https://www.ghanaweb.com/GhanaHomePage/health/Patients-in-Ho-laud-doctors-for-keeping-faith-with-them-221387) |
| MD26 | Tarkwa and Prestea doctors are not on strike | 10/10/2011 | GNA | [https://www.ghanaweb.com/GhanaHomePage/NewsArchi](https://www.ghanaweb.com/GhanaHomePage/NewsArchive/Tarkwa-and-Prestea-doctors-are-not-on-strike-221303) [ve/Tarkwa-and-Prestea-doctors-are-not-on-strike-221303](https://www.ghanaweb.com/GhanaHomePage/NewsArchive/Tarkwa-and-Prestea-doctors-are-not-on-strike-221303) |
| MD27 | Gov’t surprised at doctors, junior nurses strike | 09/10/2011 | Joy News | [https://www.ghanaweb.com/GhanaHomePage/NewsArchi](https://www.ghanaweb.com/GhanaHomePage/NewsArchive/Gov-t-surprised-at-doctors-junior-nurses-strike-221205) [ve/Gov-t-surprised-at-doctors-junior-nurses-strike-221205](https://www.ghanaweb.com/GhanaHomePage/NewsArchive/Gov-t-surprised-at-doctors-junior-nurses-strike-221205) |
| MD28 | KATH closes door to new patients | 08/11/2011 | GNA | [https://www.ghanaweb.com/GhanaHomePage/NewsArchi](https://www.ghanaweb.com/GhanaHomePage/NewsArchive/KATH-closes-door-to-new-patients-221144) [ve/KATH-closes-door-to-new-patients-221144](https://www.ghanaweb.com/GhanaHomePage/NewsArchive/KATH-closes-door-to-new-patients-221144) |
| ME1 | Health Ministry to meet FWSC over Pharmacists strike | 02/04/2012 | Radioxyzonline | [https://www.ghanaweb.com/GhanaHomePage/NewsArchi](https://www.ghanaweb.com/GhanaHomePage/NewsArchive/Health-Ministry-to-meet-FWSC-over-Pharmacists-strike-234709) [ve/Health-Ministry-to-meet-FWSC-over-Pharmacists-](https://www.ghanaweb.com/GhanaHomePage/NewsArchive/Health-Ministry-to-meet-FWSC-over-Pharmacists-strike-234709) [strike-234709](https://www.ghanaweb.com/GhanaHomePage/NewsArchive/Health-Ministry-to-meet-FWSC-over-Pharmacists-strike-234709) |
| ME2 | Striking public sector workers to lose salary | 03/04/2012 | GNA | [https://www.ghanaweb.com/GhanaHomePage/NewsArchi](https://www.ghanaweb.com/GhanaHomePage/NewsArchive/Striking-public-sector-workers-to-lose-salary-234868)  [ve/Striking-public-sector-workers-to-lose-salary-234868](https://www.ghanaweb.com/GhanaHomePage/NewsArchive/Striking-public-sector-workers-to-lose-salary-234868) |
| ME3 | Pharmacists must be realistic & honest | 08/04/2012 | GW- Amenyo, Andrew | [https://www.ghanaweb.com/GhanaHomePage/features/P](https://www.ghanaweb.com/GhanaHomePage/features/Pharmacists-must-be-realistic-honest-235310) [harmacists-must-be-realistic-honest-235310](https://www.ghanaweb.com/GhanaHomePage/features/Pharmacists-must-be-realistic-honest-235310) |
| ME4 | Pharmacists in Government Hospitals commended for calling off strike | 22/04/2012 | GNA | [https://www.ghanaweb.com/GhanaHomePage/regional/P](https://www.ghanaweb.com/GhanaHomePage/regional/Pharmacists-in-Government-Hospitals-commended-for-calling-off-strike-236631)  [harmacists-in-Government-Hospitals-commended-for-](https://www.ghanaweb.com/GhanaHomePage/regional/Pharmacists-in-Government-Hospitals-commended-for-calling-off-strike-236631) [calling-off-strike-236631](https://www.ghanaweb.com/GhanaHomePage/regional/Pharmacists-in-Government-Hospitals-commended-for-calling-off-strike-236631) |

| ME5 | Hospital Pharmacists withdraw emergency services | 29/04/2012 | Joy Online | [https://www.ghanaweb.com/GhanaHomePage/NewsArchi](https://www.ghanaweb.com/GhanaHomePage/NewsArchive/Hospital-Pharmacists-withdraw-emergency-services-272451) [ve/Hospital-Pharmacists-withdraw-emergency-services-](https://www.ghanaweb.com/GhanaHomePage/NewsArchive/Hospital-Pharmacists-withdraw-emergency-services-272451)  [272451](https://www.ghanaweb.com/GhanaHomePage/NewsArchive/Hospital-Pharmacists-withdraw-emergency-services-272451) |
| --- | --- | --- | --- | --- |
| ME6 | Gov't pharmacists vow to continue strike | 22/05/2013 | GNA | [https://www.ghanaweb.com/GhanaHomePage/health/Go](https://www.ghanaweb.com/GhanaHomePage/health/Gov-t-pharmacists-vow-to-continue-strike-274715) [v-t-pharmacists-vow-to-continue-strike-274715](https://www.ghanaweb.com/GhanaHomePage/health/Gov-t-pharmacists-vow-to-continue-strike-274715) |
| ME7 | Government pharmacists call off strike | 11/04/2012 | Myjoyonline | [https://www.myjoyonline.com/government-pharmacists-](https://www.myjoyonline.com/government-pharmacists-call-off-strike/) [call-off-strike/](https://www.myjoyonline.com/government-pharmacists-call-off-strike/) |
| ME8 | Pharmacists in Government Hospitals commended for calling off strike | 20/04/2012 | GNA | [https://www.ghanaweb.com/GhanaHomePage/regional/P](https://www.ghanaweb.com/GhanaHomePage/regional/Pharmacists-in-Government-Hospitals-commended-for-calling-off-strike-236631) [harmacists-in-Government-Hospitals-commended-for-](https://www.ghanaweb.com/GhanaHomePage/regional/Pharmacists-in-Government-Hospitals-commended-for-calling-off-strike-236631)  [calling-off-strike-236631](https://www.ghanaweb.com/GhanaHomePage/regional/Pharmacists-in-Government-Hospitals-commended-for-calling-off-strike-236631) |
| ME9 | Non-migration of GHOSPA unto the SSSS | 04/07/2012 | GNA | [https://www.ghanaweb.com/GhanaHomePage/regional/N](https://www.ghanaweb.com/GhanaHomePage/regional/Non-migration-of-GHOSPA-unto-the-SSSS-243840) [on-migration-of-GHOSPA-unto-the-SSSS-243840](https://www.ghanaweb.com/GhanaHomePage/regional/Non-migration-of-GHOSPA-unto-the-SSSS-243840) |
| MF1 | Government Pharmacists call off strike | 25/09/2012 | GNA | [https://www.ghanaweb.com/GhanaHomePage/NewsArchi](https://www.ghanaweb.com/GhanaHomePage/NewsArchive/Government-Pharmacists-call-off-strike-251295)  [ve/Government-Pharmacists-call-off-strike-251295](https://www.ghanaweb.com/GhanaHomePage/NewsArchive/Government-Pharmacists-call-off-strike-251295) |
| MF2 | Striking KATH consultants likely to call off strike today | 17/09/2012 | Radioxyonline | [https://www.ghanaweb.com/GhanaHomePage/NewsArchi](https://www.ghanaweb.com/GhanaHomePage/NewsArchive/Striking-KATH-consultants-likely-to-call-off-strike-today-250634) [ve/Striking-KATH-consultants-likely-to-call-off-strike-](https://www.ghanaweb.com/GhanaHomePage/NewsArchive/Striking-KATH-consultants-likely-to-call-off-strike-today-250634)  [today-250634](https://www.ghanaweb.com/GhanaHomePage/NewsArchive/Striking-KATH-consultants-likely-to-call-off-strike-today-250634) |
| MF3 | Korle Bu surgery department to be closed down | 15/09/2012 | Radioxyonline | [https://www.ghanaweb.com/GhanaHomePage/NewsArchi](https://www.ghanaweb.com/GhanaHomePage/NewsArchive/Korle-Bu-surgery-department-to-be-closed-down-250543)  [ve/Korle-Bu-surgery-department-to-be-closed-down-](https://www.ghanaweb.com/GhanaHomePage/NewsArchive/Korle-Bu-surgery-department-to-be-closed-down-250543) [250543](https://www.ghanaweb.com/GhanaHomePage/NewsArchive/Korle-Bu-surgery-department-to-be-closed-down-250543) |
| MF4 | Handover government properties- Bagbin charges striking pharmacists | 14/09/2012 | GNA | [https://www.ghanaweb.com/GhanaHomePage/NewsArchi](https://www.ghanaweb.com/GhanaHomePage/NewsArchive/Handover-government-properties-Bagbin-charges-striking-pharmacists-250460) [ve/Handover-government-properties-Bagbin-charges-](https://www.ghanaweb.com/GhanaHomePage/NewsArchive/Handover-government-properties-Bagbin-charges-striking-pharmacists-250460)  [striking-pharmacists-250460](https://www.ghanaweb.com/GhanaHomePage/NewsArchive/Handover-government-properties-Bagbin-charges-striking-pharmacists-250460) |
| MF5 | I am ready to face the Pharmaceutical Council - Korle-Bu CEO | 12/09/2012 | GW- Kojo Frimpong | [https://www.ghanaweb.com/GhanaHomePage/NewsArchi](https://www.ghanaweb.com/GhanaHomePage/NewsArchive/I-am-ready-to-face-the-Pharmaceutical-Council-Korle-Bu-CEO-250221)  [ve/I-am-ready-to-face-the-Pharmaceutical-Council-Korle-](https://www.ghanaweb.com/GhanaHomePage/NewsArchive/I-am-ready-to-face-the-Pharmaceutical-Council-Korle-Bu-CEO-250221) [Bu-CEO-250221](https://www.ghanaweb.com/GhanaHomePage/NewsArchive/I-am-ready-to-face-the-Pharmaceutical-Council-Korle-Bu-CEO-250221) |
| MF6 | Korle-Bu CEO Breaks Into Pharmacy To Save Lives | 11/09/2012 | Al-Hajj | [https://www.ghanaweb.com/GhanaHomePage/NewsArchi](https://www.ghanaweb.com/GhanaHomePage/NewsArchive/Korle-Bu-CEO-Breaks-Into-Pharmacy-To-Save-Lives-250077) [ve/Korle-Bu-CEO-Breaks-Into-Pharmacy-To-Save-Lives-](https://www.ghanaweb.com/GhanaHomePage/NewsArchive/Korle-Bu-CEO-Breaks-Into-Pharmacy-To-Save-Lives-250077)  [250077](https://www.ghanaweb.com/GhanaHomePage/NewsArchive/Korle-Bu-CEO-Breaks-Into-Pharmacy-To-Save-Lives-250077) |
| MF7 | Pharmacists vow to continue strike action | 10/09/2012 | GNA | [https://www.ghanaweb.com/GhanaHomePage/health/Ph](https://www.ghanaweb.com/GhanaHomePage/health/Pharmacists-vow-to-continue-strike-action-250054) [armacists-vow-to-continue-strike-action-250054](https://www.ghanaweb.com/GhanaHomePage/health/Pharmacists-vow-to-continue-strike-action-250054) |
| MF8 | Pharmacists in Brong Ahafo Regional and Sunyani Municipal hospitals strike | 04/09/2012 | GNA | [https://www.ghanaweb.com/GhanaHomePage/regional/P](https://www.ghanaweb.com/GhanaHomePage/regional/Pharmacists-in-Brong-Ahafo-Regional-and-Sunyani-Municipal-hospitals-strike-249525) [harmacists-in-Brong-Ahafo-Regional-and-Sunyani-](https://www.ghanaweb.com/GhanaHomePage/regional/Pharmacists-in-Brong-Ahafo-Regional-and-Sunyani-Municipal-hospitals-strike-249525) [Municipal-hospitals-strike-249525](https://www.ghanaweb.com/GhanaHomePage/regional/Pharmacists-in-Brong-Ahafo-Regional-and-Sunyani-Municipal-hospitals-strike-249525) |
| MF9 | Pharmacists’ strike, 'neither here nor there' - FWSC | 04/09/2012 | GNA | [https://www.ghanaweb.com/GhanaHomePage/NewsArchi](https://www.ghanaweb.com/GhanaHomePage/NewsArchive/Pharmacists-strike-neither-here-nor-there-FWSC-249515) [ve/Pharmacists-strike-neither-here-nor-there-FWSC-](https://www.ghanaweb.com/GhanaHomePage/NewsArchive/Pharmacists-strike-neither-here-nor-there-FWSC-249515)  [249515](https://www.ghanaweb.com/GhanaHomePage/NewsArchive/Pharmacists-strike-neither-here-nor-there-FWSC-249515) |
| MF10 | Don't blame us for pharmacists strike - Fair Wages | 03/09/2012 | Radioxyonline | [https://www.ghanaweb.com/GhanaHomePage/NewsArchi](https://www.ghanaweb.com/GhanaHomePage/NewsArchive/Don-t-blame-us-for-pharmacists-strike-Fair-Wages-249412)  [ve/Don-t-blame-us-for-pharmacists-strike-Fair-Wages-](https://www.ghanaweb.com/GhanaHomePage/NewsArchive/Don-t-blame-us-for-pharmacists-strike-Fair-Wages-249412) [249412](https://www.ghanaweb.com/GhanaHomePage/NewsArchive/Don-t-blame-us-for-pharmacists-strike-Fair-Wages-249412) |
| MF11 | Pharmacists threaten strike.. | 12/07/2012 | GNA | [https://www.ghanaweb.com/GhanaHomePage/NewsArchi](https://www.ghanaweb.com/GhanaHomePage/NewsArchive/Pharmacists-threaten-strike-244699) [ve/Pharmacists-threaten-strike-244699](https://www.ghanaweb.com/GhanaHomePage/NewsArchive/Pharmacists-threaten-strike-244699) |

| MF12 | Pharmacists issue new strike warning | 24/03/2012 | Ghanasoccernet | [https://ghanasoccernet.com/pharmacists-issue-new-](https://ghanasoccernet.com/pharmacists-issue-new-strike-warning) [strike-warning](https://ghanasoccernet.com/pharmacists-issue-new-strike-warning) |
| --- | --- | --- | --- | --- |
| MF13 | Post single spine salary structure technical committee inaugurated | 24/11/2012 | Ghana Business News | [https://www.ghanabusinessnews.com/2012/11/23/post-](https://www.ghanabusinessnews.com/2012/11/23/post-single-spine-salary-structure-technical-committee-inaugurated/)  [single-spine-salary-structure-technical-committee-](https://www.ghanabusinessnews.com/2012/11/23/post-single-spine-salary-structure-technical-committee-inaugurated/) [inaugurated/](https://www.ghanabusinessnews.com/2012/11/23/post-single-spine-salary-structure-technical-committee-inaugurated/) |
| MG1 | Pharmacists call off partial strike | 01/09/2015 | GNA | [https://www.ghanaweb.com/GhanaHomePage/health/Ph](https://www.ghanaweb.com/GhanaHomePage/health/Pharmacists-call-off-partial-strike-379170)  [armacists-call-off-partial-strike-379170](https://www.ghanaweb.com/GhanaHomePage/health/Pharmacists-call-off-partial-strike-379170) |
| MG2 | Pharmacists pull plug on 2nd phase of strike | 14/08/2015 | Tv3network.com | [https://www.ghanaweb.com/GhanaHomePage/health/Ph](https://www.ghanaweb.com/GhanaHomePage/health/Pharmacists-pull-plug-on-2nd-phase-of-strike-375039)  [armacists-pull-plug-on-2nd-phase-of-strike-375039](https://www.ghanaweb.com/GhanaHomePage/health/Pharmacists-pull-plug-on-2nd-phase-of-strike-375039) |
| MG3 | GHOSPA suspends 'partial strike' | 01/09/2015 | 3 News | <https://3news.com/ghospa-suspends-partial-strike/> |
| MG4 | [Public sector pharmacists call off strike](https://www.graphic.com.gh/news/general-news/public-sector-pharmacists-call-off-strike.html) | 02/09/2015 | Graphic Online | [https://www.graphic.com.gh/news/general-news/public-](https://www.graphic.com.gh/news/general-news/public-sector-pharmacists-call-off-strike.html) [sector-pharmacists-call-off-strike.html](https://www.graphic.com.gh/news/general-news/public-sector-pharmacists-call-off-strike.html) |
| MH1 | Nurses call off strike | 08/10/2015 | StarfmOnline.com | [https://www.ghanaweb.com/GhanaHomePage/health/Nu](https://www.ghanaweb.com/GhanaHomePage/health/Nurses-call-off-strike-386529)  [rses-call-off-strike-386529](https://www.ghanaweb.com/GhanaHomePage/health/Nurses-call-off-strike-386529) |
| MH2 | Mahama blames Health Ministry for nurses' strike | 07/10/2015 | StarfmOnline.com | [https://www.ghanaweb.com/GhanaHomePage/NewsArchi](https://www.ghanaweb.com/GhanaHomePage/NewsArchive/Mahama-blames-Health-Ministry-for-nurses-strike-386267) [ve/Mahama-blames-Health-Ministry-for-nurses-strike-](https://www.ghanaweb.com/GhanaHomePage/NewsArchive/Mahama-blames-Health-Ministry-for-nurses-strike-386267) [386267](https://www.ghanaweb.com/GhanaHomePage/NewsArchive/Mahama-blames-Health-Ministry-for-nurses-strike-386267) |
| MH3 | Psychiatric nurses paid outstanding salary arrears | 07/10/2015 | Tv3network.com | [https://www.ghanaweb.com/GhanaHomePage/NewsArchi](https://www.ghanaweb.com/GhanaHomePage/NewsArchive/Psychiatric-nurses-paid-outstanding-salary-arrears-386151)  [ve/Psychiatric-nurses-paid-outstanding-salary-arrears-](https://www.ghanaweb.com/GhanaHomePage/NewsArchive/Psychiatric-nurses-paid-outstanding-salary-arrears-386151) [386151](https://www.ghanaweb.com/GhanaHomePage/NewsArchive/Psychiatric-nurses-paid-outstanding-salary-arrears-386151) |
| MH4 | Mental nurses abandon post« | 04/10/2015 | Guide | [https://www.ghanaweb.com/GhanaHomePage/health/Me](https://www.ghanaweb.com/GhanaHomePage/health/Mental-nurses-abandon-post-385598) [ntal-nurses-abandon-post-385598](https://www.ghanaweb.com/GhanaHomePage/health/Mental-nurses-abandon-post-385598) |
| MI1 | MoH commends nurses for calling off strike | 13/11/2016 | classfmonline | [https://www.ghanaweb.com/GhanaHomePage/NewsArchi](https://www.ghanaweb.com/GhanaHomePage/NewsArchive/MoH-commends-nurses-for-calling-off-strike-486309) [ve/MoH-commends-nurses-for-calling-off-strike-486309](https://www.ghanaweb.com/GhanaHomePage/NewsArchive/MoH-commends-nurses-for-calling-off-strike-486309) |
| MI2 | Nurses to decide way forward today | 11/11/2016 | classfmonline | [https://www.ghanaweb.com/GhanaHomePage/NewsArchi](https://www.ghanaweb.com/GhanaHomePage/NewsArchive/Nurses-to-decide-way-forward-today-485714) [ve/Nurses-to-decide-way-forward-today-485714](https://www.ghanaweb.com/GhanaHomePage/NewsArchive/Nurses-to-decide-way-forward-today-485714) |
| MI3 | Nurses and midwives give gov't ultimatum to address their concerns | 01/11/2016 | GNA | [https://www.ghanaweb.com/GhanaHomePage/health/Nu](https://www.ghanaweb.com/GhanaHomePage/health/Nurses-and-midwives-give-gov-t-ultimatum-to-address-their-concerns-482588) [rses-and-midwives-give-gov-t-ultimatum-to-address-their-](https://www.ghanaweb.com/GhanaHomePage/health/Nurses-and-midwives-give-gov-t-ultimatum-to-address-their-concerns-482588) [concerns-482588](https://www.ghanaweb.com/GhanaHomePage/health/Nurses-and-midwives-give-gov-t-ultimatum-to-address-their-concerns-482588) |
| MI4 | Nurses threaten national strike | 29/10/2016 | Daily Guide Africa | https:/[/www.ghanaweb.com/GhanaHomePage/health/Nu](http://www.ghanaweb.com/GhanaHomePage/health/Nu) rses-threaten-national-strike-481828 |
| MI5 | Nurses’ strike affecting mental healthcare - Chief Psychiatrist | 03/11/2016 | Todaygh.com | [https://www.ghanaweb.com/GhanaHomePage/NewsArchi](https://www.ghanaweb.com/GhanaHomePage/NewsArchive/Nurses-strike-affecting-mental-healthcare-Chief-Psychiatrist-483283) [ve/Nurses-strike-affecting-mental-healthcare-Chief-](https://www.ghanaweb.com/GhanaHomePage/NewsArchive/Nurses-strike-affecting-mental-healthcare-Chief-Psychiatrist-483283) [Psychiatrist-483283](https://www.ghanaweb.com/GhanaHomePage/NewsArchive/Nurses-strike-affecting-mental-healthcare-Chief-Psychiatrist-483283) |
| MI6 | Mahama’s comment on Psychiatric Hospital misconstrued - Victor Bampoe | 02/10/2016 | Starrfmonline.com | [https://www.ghanaweb.com/GhanaHomePage/NewsArchi](https://www.ghanaweb.com/GhanaHomePage/NewsArchive/Mahama-s-comment-on-Psychiatric-Hospital-misconstrued-Victor-Bampoe-483092)  [ve/Mahama-s-comment-on-Psychiatric-Hospital-](https://www.ghanaweb.com/GhanaHomePage/NewsArchive/Mahama-s-comment-on-Psychiatric-Hospital-misconstrued-Victor-Bampoe-483092) [misconstrued-Victor-Bampoe-483092](https://www.ghanaweb.com/GhanaHomePage/NewsArchive/Mahama-s-comment-on-Psychiatric-Hospital-misconstrued-Victor-Bampoe-483092) |
| MI7 | Psychiatric Hospital 'frees' patients over strike | 01/10/2016 | Starrfmonline.com | [https://www.ghanaweb.com/GhanaHomePage/health/Psy](https://www.ghanaweb.com/GhanaHomePage/health/Psychiatric-Hospital-frees-patients-over-strike-482753) [chiatric-Hospital-frees-patients-over-strike-482753](https://www.ghanaweb.com/GhanaHomePage/health/Psychiatric-Hospital-frees-patients-over-strike-482753) |
| MI8 | Psychiatric hospital risks shutdown | 01/10/2016 | Starrfmonline.com | [https://www.ghanaweb.com/GhanaHomePage/NewsArchi](https://www.ghanaweb.com/GhanaHomePage/NewsArchive/Psychiatric-hospital-risks-shutdown-482647) [ve/Psychiatric-hospital-risks-shutdown-482647](https://www.ghanaweb.com/GhanaHomePage/NewsArchive/Psychiatric-hospital-risks-shutdown-482647) |

| J1 | THE SINGLE SPINE PAY POLICY: CAN IGNORANCE DERAIL THE  BENEFITS IT HAS ON THE GHANAIAN PUBLIC SERVICE WORKER? | Apr-14 | Seniwoliba JA | Seniwoliba JA. The Single Spine Pay Policy: Can Ignorance Derail the Benefits it Has on the Ghanaian Public Service Worker?. European Scientific Journal. 2014 Mar 1;10(8). |
| --- | --- | --- | --- | --- |
| J2 | The imperative of evidence-based health workforce planning and implementation: lessons from nurses and midwives unemployment crisis in Ghana | Dec-20 | Asamani et al | Asamani JA, Amertil NP, Ismaila H, Akugri FA, Nabyonga- Orem J. The imperative of evidence-based health workforce planning and implementation: lessons from nurses and midwives unemployment crisis in Ghana.  Human resources for health. 2020 Dec;18(1):1-6. |
| J3 | When ‘solutions of yesterday become problems of today’: crisis-ridden decision making in a complex adaptive system (CAS)—the Additional Duty Hours Allowance in Ghana | Oct-12 | Agyepong et al | Agyepong IA, Kodua A, Adjei S, Adam T. When ‘solutions of yesterday become problems of today’: crisis-ridden decision making in a complex adaptive system (CAS)—the  Additional Duty Hours Allowance in Ghana. Health policy and planning. 2012 Oct 1;27(suppl_4):iv20-31. |
| J4 | Health worker (internal customer) satisfaction and motivation in the public sector in Ghana | Oct-04 | Agyepong et al | Agyepong IA, Anafi P, Asiamah E, Ansah EK, Ashon DA, Narh‐Dometey C. Health worker (internal customer) satisfaction and motivation in the public sector in Ghana.  The International journal of health planning and management. 2004 Oct;19(4):319-36. |
| J5 | Association between health worker motivation and healthcare quality efforts in Ghana | Dec-13 | Alhassan et al | Alhassan RK, Spieker N, van Ostenberg P, Ogink A, Nketiah- Amponsah E, de Wit TF. Association between health worker motivation and healthcare quality efforts in Ghana.  Human resources for health. 2013 Dec;11(1):1-1. |
| J6 | An evaluation of the effects of industrial unrest at the Korle-Bu Teaching Hospital (KBTH), Accra, Ghana | Aug-15 | Awori and Tettey-Enyo | Awori SN, Tettey-Enyo A. An evaluation of the effects of industrial unrest at the Korle-Bu Teaching Hospital (KBTH),  Accra, Ghana. |
| J7 | Ethical Leadership, Job Satisfaction, and Organisational Commitment among Health Workers in Ghana: Evidence from Central Region Hospitals | Feb-22 | Amoah et al | Amoah C, Jehu-Appiah J, Boateng EA. Ethical Leadership, Job Satisfaction, and Organisational Commitment among Health Workers in Ghana: Evidence from Central Region  Hospitals. Journal of Human Resource and Sustainability Studies. 2022 Feb 24;10(1):123-41. |
| J8 | Assessing the effects of industrial unrest on Ghana health service: A case study of nurses at Korle-Bu teaching hospital | Jan-11 | Gerald DG | Gerald DG. Assessing the effects of industrial unrest on Ghana health service: A case study of nurses at Korle-Bu  teaching hospital. International Journal of Nursing and Midwifery. 2011 Jan 31;3(1):1-5 |
| J9 | Ghana’s numerous workers strikes; a cause for concern | Sep-13 | Seniwoliba JA | Seniwoliba JA. Ghana’s numerous workers strikes; a cause for concern. |
| J10 | The impact of strike action by Ghana registered nurses and midwives on the access to and utilization of healthcare services | Oct-22 | Ampofo et al | Ampofo PO, Tenkorang-Twum D, Adjorlolo S, Chandi MG, Wuni FK, Asiedu E, Kukula VA, Opoku S. The impact of strike action by Ghana registered nurses and midwives on  the access to and utilization of healthcare services. Plos one. 2022 Oct 14;17(10):e0275661. |

| J11 | Challenges of Single Spine Salary Structure in Ghana | Dec-17 | Asare and Mpere | Asare BE, Mpere DL. Challenges of Single Spine Salary Structure in Ghana. The African Review: A Journal of  African Politics, Development and International Affairs. 2017 Dec 1:203-23. |
| --- | --- | --- | --- | --- |
| J12 | Single spine, double spine or multiple spine: solving labour conflicts and making public sector jobs more rewarding in Ghana | Dec-14 | Brako and Asah-Asante | Brako I, Asah-Asante K. Single spine, double spine or multiple spine: solving labour conflicts and making public sector jobs more rewarding in Ghana. Modern Africa:  politics, history and society. 2014 Dec 30;2(2):117-40. |
| J13 | IMPLEMENTATION OF SINGLE SPINE PAY POLICY IN PUBLIC  SECTOR OF GHANA: ANALYSIS AND RECOMMENDATIONS  FROM ORGANIZATIONAL DEVELOPMENT PERSPECTIVE | Dec-15 | Oppong, Dickson and Asument | Oppong S, Dickson E, Asumeng M. Implementation of Single Spine Pay Policy in Public Sector of Ghana: Analysis and Recommendations from Organizational Development Perspective. Poslovna izvrsnost. 2015 Dec 20;9(2):83-99. |
| J14 | The evolving partnership between the Government of Ghana and national faith-based health providers: leadership perspective and experiences from the Christian Health Association  of Ghana | May-17 | Yeboah and Buckle | Yeboah P, Buckle G. The evolving partnership between the Government of Ghana and national faith-based health providers: leadership perspective and experiences from the Christian Health Association of Ghana. Development in  Practice. 2017 Jul 4;27(5):766-74. |
| J15 | Towards universal health coverage: a mixed-method study mapping the development of the faith-based non- profit sector in the Ghanaian health system | Dec-18 | Grieve and Olivier | Grieve A, Olivier J. Towards universal health coverage: a mixed-method study mapping the development of the faith-based non-profit sector in the Ghanaian health  system. International Journal for Equity in Health. 2018 Dec;17(1):1-20. |
| J16 | Models of engagement between the state and the faith sector in sub-Saharan Africa–a systematic review | Jul-17 | Whyle and Olivier | Whyle E, Olivier J. Models of engagement between the state and the faith sector in sub-Saharan Africa–a  systematic review. Development in Practice. 2017 Jul 4;27(5):684-97. |
| J17 | The history of public‐ (faith‐based) private health sector partnership in Ghana. Report  for the Alliance for Health Policy and Systems Research, World Health Organisation: Geneva | 2018 | Olivier and Kwame | Olivier J, Kwamie A. 2017. The history of public‐ (faith‐based) private health sector partnership in Ghana. World Health Organisation, Geneva, Switzerland. |
| T1 | THE IMPLEMENTATION OF THE SINGLE SPINE SALARY STRUCTURE (SSSS) IN GHANA | Jul-15 | Larbe M | Larbi MD. The Implementation of the Single Spine Salary  Structure (Ssss) in Ghana (Doctoral dissertation, University of Ghana). |
| T2 | PUBLIC-PRIVATE PARTNERSHIP IN THE CONTEXT OF  GHANA’S HEALTH SECTOR REFORM: A CASE STUDY OF  PRIVATE NOT-FOR-PROFIT ORGANISATIONS IN THE VOLTA REGION OF GHANA | Dec-14 | Adzei F | Adzei FA. Public-Private Partnership in the Context of Ghana’s Health Sector Reform: A Case Study of Private  Not-For-Profit Organisations in the Volta Region of Ghana (Doctoral dissertation, University of Ghana). |
| T3 | THE LABOUR ACT 2003, ACT 651 AND ORGANIZED LABOUR STRIKES:  CASE STUDY OF GHANA MEDICAL ASSOCIATION | Sep-12 | Martinson C | Martinson C. The Labour Act 2003, Act 651 and Organized Labour Strikes: Case Study of Ghana Medical Association.  Unpublished Masters’ thesis. Kwame Nkrumah University of science and Technology: Kumasi. 2012 |
| T4 | STRIKE AS A TOOL FOR RESOLVING EMPLOYEE DISSATISFACTION  A CASE STUDY OF KOMFO ANOKYE TEACHING HOSPITAL (KATH) | Jan-14 | Addison M |  |

| T5 | Towards Universal Health Coverage: Mapping the Development of the Faith-Based Non-Profit Sector in the  Ghanaian Health System | Sep-17 | Grieve A |  |
| --- | --- | --- | --- | --- |
| T6 | An organisational typology of public-private engagement for health in Southern Africa: A systematic review | Mar-15 | Whyle E |  |
| P1 | Labour Act 651 Ghana |  |  | [https://www.ilo.org/legacy/english/inwork/cb-policy-](https://www.ilo.org/legacy/english/inwork/cb-policy-guide/ghanalabouract2003section109.pdf) [guide/ghanalabouract2003section109.pdf](https://www.ilo.org/legacy/english/inwork/cb-policy-guide/ghanalabouract2003section109.pdf) |
| P2 | Constitution of Ghana |  |  | [https://www.constituteproject.org/constitution/Ghana_1](https://www.constituteproject.org/constitution/Ghana_1996.pdf)  [996.pdf](https://www.constituteproject.org/constitution/Ghana_1996.pdf) |
| P3 | Pharmacy Act 1994 (Act 489) |  |  | [https://www.moh.gov.gh/wp-](https://www.moh.gov.gh/wp-content/uploads/2016/02/Pharmacy-Act-1994-Act-489.pdf) [content/uploads/2016/02/Pharmacy-Act-1994-Act-](https://www.moh.gov.gh/wp-content/uploads/2016/02/Pharmacy-Act-1994-Act-489.pdf)  [489.pdf](https://www.moh.gov.gh/wp-content/uploads/2016/02/Pharmacy-Act-1994-Act-489.pdf) |
| P4 | Memorandum of Understanding Ministry of Health and  Christian Health Associaton of Ghana |  |  |  |
| P5 | Draft Conditions of service | Jun-15 | Ministry of Health |  |
| C1 | Support for Doctors n CHAG-member facilities within the context of the ongoing doctors' strike action | Aug-15 | Christian Health Association of Ghana | |
| C2 | Withdrawal of OPD services by medical in government hospitals and clinics | Aug-15 | Christian Health Association of Ghana |  |
