# Supplementary material for: Public–Private engagement and health systems resilience in times of health worker strikes: a Ghanaian case study
Source: Health Policy Plan. 2024 Mar 18;39(5):469–85. doi: 10.1093/heapol/czae018 (PMC11095267; doi:10.1093/heapol/czae018)
Supplement: czae018_Supp [file czae018_supp.zip › Supplementary 3. Expanded Table Characteristics of Strikes Incidents .docx]

**Supplementary 3**

Characteristics of health workers strike incidents in Ghana from 2010-2016 (Full Table)

| **Strike Incident** | **Striking body** | **Onset characteristics** | **Macro socio-economic and political factors** | **Resolution** | **Impact** |
| --- | --- | --- | --- | --- | --- |
| 7 Oct-27 Oct 2011  Code: D | GMA | Dispute between GMA and FWSC over alleged distortions in grading structures SSSS and determination of market premium by the FWSC. MD3, MD5, MD7, MD9, MD20, MD21, MD22, MD27, J1, J8, J9, T4 | -NDC gov led by President John Atta Mills.  -Period of relatively strong economic growth, driven in part by the discovery of large oil reserves. In 2010 the country achieved middle-income status. MD5, MD8, MD28, J1 | -Appeals by civil groups (esp. clergy) for strikes to end  -NLC intervention, compelling doctors to undergo compulsory arbitration. NLC rules in FWSC favor of FWSCMD2, MD11, MD7, MD13, MD14, J9 | -Doc withdrew OPD services at most gov hospitals. Inpatient and emergency services continued during strike.  -QHI struggle to handle increased patient load  -Strikes have disproportionate effect on Northern regions with fewer private hospitals.  -Strikes result in low staff motivation MD9, MD12, MD23, J5(2) |
| 1 April- 22 April 2012  Code: E | GHOSPA | Dispute between FWSC and GHOSPA about implementation of the NLC ruling on grading structure, market premium and conversion difference of pharmacists being migrated onto the SSSS. (ME1, ME5, ME8, J1, J8, J9, J10, J11(212), T4) | - Death of President Mills, President Mahama accedes as president on the 24th of July  - Growing political tensions -general elections December 2012 and expected to be highly contentious.(ME9, J1) | -Grievance Review Committee set up by gov to attend to impasse between GHOSPA and FWSC. (ME4, ME7) | -Strikes began with the withdrawal of OPD. Reports indicate that the strikes escalated to a point where HIV patients and those with mental illnesses were also denied services.  -High revenue losses incurred by clinics  -Strikes result in low staff motivationME6, ME8, ME9, J5(2), P3 |
| 4 September-25 September 2012  Code: F | GHOSPA | Dispute between FWSC and GHOSPA over delay in migration of government pharmacists onto the SSSS. FWSC claims the delay was due to the leadership of GHOSPA who sought a delay in their migration due to errors it perceived with salary grade structure. (MF1, MF4, MF7, MF9, MF11, MF12, J1, J8, J9, J11(212), J13, T4) | - Death of President Mills, President Mahama accedes as president on the 24th of July  - Growing political tensions -general elections December 2012 and expected to be highly contentious. (ME9, J1) | -FWSC and GHOSPA to appear before a compulsory arbitration panel. According to one report GHOSPA lost the case.  -In November 2012, the government inaugurated the Single Spine Post Migration Technical Committee to respond to all issues of migration onto the SSSS. (MF1, MF4, MF13, J9) | -Emergency services were withdrawn from the 11^th^ of September.  -Heightened tensions between hospital administrators and Pharmacists. R  -Some surgery department had to close down.  -Financial strain on hospitals as significant revenue brought in by pharmacy sales.  -Low staff motivation (MF2, MF3, MF5, MF6, MF7, MF8, J5(2)) |
| 8 April-8 May 2013  Code: A | GMA | -Doctors striking for payment of their market arrears which were outstanding since being migrated onto the SSSS. On March 27, 2013, the NLC adopted a payment schedule drawn up by the FWSC in which payment of outstanding arrears would occur in three instalments. This payment plan was rejected by the GMA.  -GMA also striking against reduction in their pension after migration onto the SSSS. MA1, MA4, MA8, MA12, MA12, J5, J11, J8, J9, J11(212), J12 | -NDC President Mahama won December 2012 elections with a narrow margin h  -Several public sector strikes occurring in the nation- primary, secondary teachers and university lecturers on strike.  -Energy crisis, economic slowdown and budget deficit.  MA4 | Strike called off after a ruling by the NLC that the gov should restore the conversion difference being sought after by the striking doctors. (MA3, MA7, J9, J11(212)) | -OPD were withdrawn by doctors from 8-14 April. Doctors threatened that after the 22^nd^ of April they would suspend all emergency services in hospitals.  -Disproportionate harm on vulnerable patient groups i.e., diabetic patients.  - Crippling public sector as strike occurred congruent with other strikes in the primary and tertiary sector  MA1, MA7, MA8, MA12, MA34, |
| 8 April- 18 June  2013  Code: A | GHOSPA | Dispute between GHOSPA and the FWSC over salary grading structure and market premiums. GHOSPA was demanding that the FWSC place them on the correct grading structure as per the ruling of the NLC which was made in their favor of the 16^th^ of April that year. J8, J9, J11(212) |  | Strike ended after the NLC took the FWSC to court to compel the FWSC to enforce their directive given to the FWSC for the payment of conversion differences. The Accra High Court ruled in favor of FWSC.J9, J11(212) | Pharmacists initially withdrew OPD services and escalated their strike action to withdraw emergency services as well from the 29^th^ of April. Pharmacists also withdrew services to patients with HIV and those living with mental illness.MA1, MA7, MA8, MA12 |
| 30 July- 24 August 2015  Code: B | GMA | GMA demanding gov finalize their conditions of service; an issue which had been outstanding including how additional doctors work each month was calculated and reimbursed. According to the GMA they received no response from government after seven months.  MB1, MB4, MB5, MB9, MB14, MB20, MB22, MB28, MB30, MB1, MB9, MB14, MB16, MB30, J8, P5, C1 | -Political tension rising as 2016 due to contentious elections.  -Slow economic growth in Ghana characterized by rising debt and a ballooning public wage bill.  - IMF loan condition for gov to reduce spending on public wages. MB1, MB4, MB5, MB9, MB14, MB20, MB22, MB28, MB30 MB17, MB22, MB24, MB47, MB54, J1, J2 | -GMA called off strike after calls from multiple groups for the doctors to return to hospitals.  -Executive meeting held by GMA where decision to end strike was taken; but decision not welcomed by all in the GMA.  -Directive issued by NLC for GMA to engage in compulsory arbitration  MB1, MB2, MB6, MB7, MB35, MB41, MB3, MB8, P5 | -In the first week of the strike outpatient services were withdrawn with inpatient and emergency services continuing. In the second week emergency services were.  -Reports that 500 patients had died since onset of strike. MB17, MB8, MB9, MB14, MB19, MB21, MB28, MB31, MB42, MB45, MB61 |
| 3 August- 31 August 2015  Code: G | GHOSPA | GHOSPA grievances over gov handling off and inadequately dealing with dispute over SSSS. Complaints from pharmacists that after migration onto the SSSS their salaries turned out to be lower. MG1, MG2, MG3, MG4, J8 |  | Decision to scale up strike withdrawn after assurances form government that it would attend to grievances. Suspension also in response to persistent please from public. MG1, MG3 | Partial strike- Pharmacists withdrew dispension of drugs in OPD as well as night and weekend services. (MG1, MG2, MG3) |
| 1 October-8 October 2015  Code: I | Coalition of unpaid nurses and psychiatric nurses | 7000 junior nurses and midwives striking due to delay in salary payments. The coalition claimed outstanding salaries for periods ranging from six to eleven months. MI1, M12, M13, J2, J8 |  | Psychiatric nurses strike ended after the Controller Accounts General transferred outstanding salary arrears to nurses.  M13, MI4 | Psychiatric hospital potentially needing to release patients and risk closing down. (MI4) |
| 5 September- 10 October 2016  Code: C | GHOSPA | GHOSPA demanding that their salary grade structure, interim market premium and conditions of service should be reviewed by the FWSC (MC1, MC2, MC3, MC6, T1, MC3, MC4, MC5, MC6, MC9, MC10, MC11, MC12, MC13, MC14, J8) | -Rising political tension over National elections to be held in October.  Continued slow economic growth.  MC12, MC14, J8 | -Appeals from civil society for an end to the strikes  -At a National Executive meeting of GHOSPA on 10 October where members agreed to end strike after mounting public pressure.  -MOH instituted a committee to investigate strike.  MC12, MC14, J8 | Shutdown of gov pharmacies. Only inpatients attended to. (MC1, MC5, MC7, MC11, MC14, *MC6, MC7)* |
| 7 November- 11 November 2016  Code: J | GRNMA | -GRNMA grievances over unpaid salary arrears, posting of nursing graduates, charging of promotional fees and inadequate logistics for public facilities. Association claimed 2000 members employed as far back as 2014 yet to receive payment  -Long-standing issue of poor conditions of service for nurses. MJ2, M J3, MJ4, MJ5, MJ6, MJ8, J6, J8, J9 | -Rising political tension over National elections to be held in October. Continued slow economic growth. | Strike called off after o- GRNMA gives government until February of the next year to attend to their grievances. MJ1, MJ2 | Psychiatric hospitals during the strike had to release patients and risked being shut down. MJ4, MJ5, MJ6, MJ8 |

Shaded cells= embedded strike incidents. NDC National Democratic Congress, Doc Doctor, GMA Ghana Medical Association, GHOSPA Government Hospital Pharmacists Association, GNRMA Ghana Registered Nurses and Midwives Association, QHI Quasi-governmental health institutions, Gov Government, SSSS, Single Spine Salary Structure,
