# Supplementary material for: Public–Private engagement and health systems resilience in times of health worker strikes: a Ghanaian case study
Source: Health Policy Plan. 2024 Mar 18;39(5):469–85. doi: 10.1093/heapol/czae018 (PMC11095267; doi:10.1093/heapol/czae018)
Supplement: czae018_Supp [file czae018_supp.zip › Supplementary 4- Themes context of health worker strikes (embedded strike incidents).docx]

## Supplementary 4: Themes context of health worker strikes (embedded strike incidents)

| **Themes** | **2013 GMA Strike** | **2015 GMA Strike** | **2016 GHOSPA Strike** |
| --- | --- | --- | --- |
| **Duration** | 8 April 2013-8 May 2013 | July 30, 205-August 24, 2015 | 5 September 2016-10 October 2016 |
| **Actors** | *Professional workers union*  -Doctors represented by the Ghana Medical Association (MA9-MA14), J5  *Fair wages and salary commission*  *-*Doctors seeking for payment schedule proposed by FWSC to be overturned.  MA8, MA11, MA12, J5  *National Labour Commission*  *-*Doctors seeking the NLC to overturn payment schedule of FWSC  -Strike resolved in part by intervention from NLC MA9, MA10, MA11, MA3, J5, J11  *Minister of Health*  *-*Condemns doctors for striking, referring to the strikes as unlawful.  -Directed by Presidency to ensure protection of health of citizens.  MA10,  *Prominent political figures and groups calling for end to strikes*  *-*Kofi Annan  -Clergy calls for resolution MA7, MA14  *Condemnation from other governmental bodies*  -The government federation of labour condemned doctors for striking  *MA9, MA5* | *Pharmacists represented by GHOSPA*  MB26 (partially withdrawn services)  -Psychiatric nurses (MB50, 57, 67)  *National Labour Commission*  -Gave directive for doctors to return to work. MB1, MB2  -Unable to enforce own ruling. Credibility as arbitrator questioned (MB9)  MB1, MB2, MB9, MB39,  *President Mahama*  -Describes strikes as illegal MB23  -Declares not a single cedi outside the budget on unplanned expenditure  MB24, MB25  -Comments aggravating strikes MB36 MB23, MB24, MB25, MB36  *Prominent political figures and groups calling for end to strikes:*  MB15, MB26  *Minister of Health*  -Condemns doctors for striking, referring to the strikes as unlawful.  -Threatened to freeze the salaries of doctors (did not manifest) MB25  -Issue directive for doctors to return to work  -Bringing in Cuban doctors to work in hospitals (MB12, MB13)  *MB5, MB12, MB13, MB18, M25*  *Minister of Employment and Labour Relations*  -Agreement with doctors to sign codified conditions of service by September 30.  MB4, MB5, MB9, | *Professional workers union*  -600 members of the Government Hospital Pharmacist Association. MC1-MC16  *Fair wages and salary commission*  -Being asked by pharmacists to address the issue of their market premiums MC1, MC2, MC3, MC5, MC6, MC8, MC13  *National Labour Commission*  -Blamed for delay in responding to pharmacists grievances  -Took GHOSPA to court (MC8) MC1, MC2, MC3, MC4, MC5, MC6  *Employment and Labour Relations Minister*  -Minister states there is no budget to pay striking pharmacists as the demanded for market premium adjustment was not accounted for in budget  MC8,  *Labour Division of the High Court*  -NLC filed a complaint against GHOSPA at the labour division of the High Court MC8  *Ministry of Health*  -Called on by GHOSPA to resolve their challenges MC12  -Gave directive for other health workers to act as pharmacists in public hospitals  MC11 |

| **Strike Onset** | *Salary Disputes*  -Decrease in salaries due to placement of doctors on the Single Spine Salary Structure.  MA1, MA4, MA8, MA12, MA12, J5, J11  *SSSS*  -Complaint of reduction of pensions of doctors since being placed on the SSSS.  MA1, MA4, MA8, MA12, MA13, J11, T1  *Delays by Fair Wages and Salary Commission*  -Failure of the Fair Wages and Salary Commission to pay the difference between salaries prior to movement onto SSSS.  MA1, MA4, MA8, MA12, MA13, J11  *Conditions of service*  MA12, MA13, J11 | *Conditions of service*  -Uncodified conditions of service for Ghanaian doctors. Issue had been a long-standing issue. GMA notified government of request to formalise conditions of service seven months prior.   - include a formula for calculating how additional hours doctors work each month are calculated and remunerated   MB1, MB4, MB5, MB9, MB14, MB20, MB22, MB28, MB30  *Delayed response*  -GMA states government did not respond to request after 7months (MB16) and therefore embarked on strike action.  MB1, MB9, MB14, MB16, MB30  *Breakdown in negotiations*  MB13, MB15, MB18, MB23, MB29, MB44  *Salary dispute*  -Strikes not directly related to salaries but conditions of service affect overall salaries.  -Salary not the main issue   - Pharmacists complaint over salaries MB22, MB30, MB43, MB48, MB68,67,69 MB48, MB68, MB67,69   *Payment arrears*  -Psychiatric nurses demanding government pay back their payment arrears MB50, MB57 | *Salary disputes*  -GHOSPA demanding that their salary grade structure, interim market premium and conditions of service should be reviewed by the Fair Wages and Salaries Commission.  MC1, MC2, MC3, MC6, MC9, MC12, MC13  *Wage disparities*  *-*Pharmacist claim that market premium they received was lower than other pharmacists at quasi-government facilities  MC1, MC2, MC3, MC6, MC9, MC14, T1  *SSSS*  -The pharmacists claimed that since the inception the SSSS seven years ago, pharmacists in government hospitals had been receiving lower salaries than their counterparts in other quasi-government institutions such as the Legon Hospital and the Food and Drugs Authority.  MC1, MC2, MC3, MC6, T1  *Delayed Response*  -Ongoing issue for six years with no resolution  MC3, MC4, MC5, MC6, MC9, MC10, MC11, MC12, MC13, MC14  *Breakdown in negotiations*  MC3, MC4, MC5, MC6, MC8, MC9, MC10, MC11, MC13, MC14  *Conditions of service*  *-demand by pharmacists for their condition of service to be reviewed MC1, MC3, MC6, MC13*  *Previous unsuccessful strikes*  *-Previous strikes reported by GHOSPA in 2012 and 2013 MC12* |
| --- | --- | --- | --- |
| **Strike resolution** | -*National Labour Commission intervened* and ordered government to pay the conversion difference to doctors in three instalments.  -Meeting between GMA, Trade Union Congress for the formation of a Single Spine Post Migration Committee to address the conversion difference and pension  MA3  *Persuasion by external actor* | *Persuasion by external actors*  -A number of actors joined forces to persuade the doctors to end the strike, including the National House of Chief, various religious organisations, political groups  MB2, MB6, MB7, MB35, MB41,  -GMA executive due to pressure from actors to end strike decided to call of the strikes and to continue negotiations with government.  -Decision to end strike not welcomed by all in the GMA. | *Committee to review*  The MOH instituted a committee to look into the strike which developed a report titled: “Road map for Implementation of Recommendations on GHOSPA.  -An emergency national meeting was held by GHOSPA on 10 October 2016 to decide if the strike should be called off. At the meeting GHOSPA members agreed to call of the strike and return to work |

|  | *MA7* | MB3, MB8,  -Directive from NLC to return to negotiating table MB1, |  |
| --- | --- | --- | --- |
| **Strike characteristics and impact** | *Withdrawal of outpatient and emergency services*  -Outpatient services were initially withdrawn, then in third week emergency services were withdrawn by doctors at almost all public hospitals in the nation.  MA1, MA7, MA8, MA12  *Inpatients attended to MA1, MA8 Congruent with other public sector strikes*  - *Primary and secondary school teachers and university lecturers were also on strike MA4* | *Withdrawal of outpatient and emergency services*  -Outpatient services were initially withdrawn, then in third week emergency services were withdrawn by doctors at almost all public hospitals in the nation- including district hospitals (MB9). Doctors threatened to resign in masse if their demands were not met, but strikes ended before this occurred (MB28, MB58, MB60). | *Withdrawal of services*  Led to a complete shutdown of national pharmacies  -Only inpatients attended to. MC1, MC5, MC7, MC11, MC14 |
|  |  | MB8, MB9, MB14, MB19, MB21, MB28, MB31, MB42, MB45, MB61 | *Inpatients attended to* |
|  |  | *Court involvement* |  |
|  |  | -Case brought against the GMA by the Union Front Party MB23 | *-Inpatients admitted prior to the onset of the strikes were attended to MC6, MC* |
|  |  | *Increased mortality* |  |
|  |  | -According to HIPSGA approximately 500 patients died during the first two weeks of the strikes. Number is estimated to be related to strikes as many patients died while being transported from one hospital to another. Number suspected to be under-reported due to rural numbers not being included. |  |
|  |  | *Nurses at hospitals providing basic care* MB42, MB44 |  |
|  |  | *Perception of severe impact on patients* |  |
|  |  | *-*Minister pleading with doctors to go back for fear of strikes having lasting public health impacts MB29 |  |
|  |  | -vulnerable patient groups i.e. diabetes MB34, *MB10, MB11, MB13, MB16, MB17, MB19, MB21, MB28, MB29, MB32, MB24, MB42* |  |
| **Macro political and economic factors** | *Congruent with other public sector strikes MA4*  *Primary and secondary school teachers and university*  *lecturers were also on strike* | *Slow economic growth, rising debt*  -Ghanaian economy at time characterized by slow economic growth, rising debt and a large budget deficit primarily due to the 2012 passage of a public sector wage bill. | *Economy*  *-Continued slow economic growth and rising debt MC4, MC5* |
|  | *MA4* | -IMF loan to Ghana with condition of reducing public sector wage bill  from 55% to 30%. | *Elections* |
|  | *Political Elite (MA4)*  *Economy*  *-Energy crisis, economic slowdown and budget deficit* | -Energy crisis (MB26,MB47,MB54) MB17, MB22, MB26, MB47, MB54  *Political Elections* | *-*Rising political tensions ahead *of* the 2016 election year in Ghana with elections held in December. There was growing discontent with the NDC Mahama administration. MC4, MC5 |
|  | *MA4* | -Rising political tension in nation due to national government elections in the following year. |  |

|  |  | MB17, MB22, MB24, MB47  Narrative of strikes used by political parties  -Case brought against the GMA by the Union Front Party (MB23)  -NPP accused of being behind the doctors strikes (MB24, 47)  -NPP condemns NDC handling of strikes (MB24, MB55, MB58)  -GMA claims strikes not politically fuelled (MB48) MB11, MB17, MB22, MB26, MB47, MB48, MB55, MB58  *Congruent with other public sector strikes* MB58  -University Teachers Association of Ghana also on strike MB41 |  |
| --- | --- | --- | --- |
| Aggravating factors and impact | *Allowances paid to parliamentarians*  -Prior to onset of strikes, fifty thousand Ghana Cedis was paid to Ghanaian parliamentarians as rent money. Six weeks later, forty six thousand Cedis was paid as exgracia. | *Condemnation from other governmental bodies MB10*  *Directives from government perceived as intimidation*  *MB12,* | -Directive issues by government  Directive was issued by government for the pharmacists to return to work, heightens tensions  *Perception of severe impact on patients* |
|  | *Condemnation from other governmental bodies* | *Language* | *Patients affected* |
|  |  | *-Aggressive language used by those in the government (especially party communicators in the media and the GMA increasing tensions and other political commentators)* | MC1,MC5 |
|  |  | *MB3, MB6, MB7, MB10, MB15, MB20, MB26, MB28, MB30, MB31, MB31, MB34, MB38, MB59* |  |
|  |  | *-Between political parties MB24, MB26* |  |
|  |  | *-Using of unprintable words MB59* |  |
|  |  | *Allowances paid to parliamentarians MB17,* |  |
