# Supplementary material for: Public–Private engagement and health systems resilience in times of health worker strikes: a Ghanaian case study
Source: Health Policy Plan. 2024 Mar 18;39(5):469–85. doi: 10.1093/heapol/czae018 (PMC11095267; doi:10.1093/heapol/czae018)
Supplement: czae018_Supp [file czae018_supp.zip › Supplementary 5. Expanded Table Quotes Contributing to Themes.docx]

## Supplementary 5 Expanded table themes from embedded cases - engagement of CHAG and GoG during health worker strikes

|  | **2013** | **2015** | **2016** |
| --- | --- | --- | --- |
| **Theme:** | **Sub-theme: Patients publicly redirected to** | **Sub-theme: Patients publicly redirected to CHAG facilities** | **Sub-theme: Patients publicly redirected to CHAG** |
| **Coordination at** | **CHAG facilities** | MB44, MB18, MB70, MB71, J14, J15, T2, C1 | **facilities** |
| **national level to** | MA20, E7, S1, J14, J15, T2 | *-“Patients were referred to private hospitals and quasi-government institutions due* | MC1, MC31, J14(770), T2, P3 |
| **continue service** | *“Managers of health facilities must be in* | *to the GMA withdrawing emergency and OPD services during the strike.”* | *“Accordingly, the general public could access* |
| **delivery** | *constant communication with quasi health* | *-“Managers of health facilities must be in constant communication with quasi health* | *pharmaceutical services in those facilities,” the* |
|  | *institutions to ensure the delivery of* | *institutions to ensure thedelivery of seamless emergency care during the period”* | *statement said in part.” (MC31) (MC31)* |
|  | *seamless emergency care during the* | *List of availablehospitals released- MB18,23, 28* | *“It also directed all service delivery Agencies (GHS,* |
|  | *period”* | *-“Friends from the media, additionally, about 25 quasi health facilities, 178* | *CHAG, Teaching Hospitals) to comply with some* |
|  | **Sub-theme: CHAG non-striking position** | *hospitals/clinics of the Christian Health Association of Ghana (CHAG), private health* | *interim directives whilst they try to resolve the issues”* |
|  | **ensures facilities remain open** | *careproviders and retired doctors across the country are supporting in delivering* | *(MC31) (MC31)(43)* |
|  | MA20, E7 | *healthcare”- (MB70) (MB71)* | **Sub-theme: CHAG non-striking position ensures** |
|  | *“the government is grateful to the Police,* | *-“I take this opportunity to thank all members of the GMA and other health workers* | **facilities remain open** |
|  | *Military, Christian Health Association* | *who have continued to work; the Christian Health Association of Ghana (CHAG)”* | MC31, J14(770) |
|  | *hospitals, all other health professionals and* | *(MB70)* | *“We are also pleased to announce to the public that* |
|  | *traditional medicine practitioners for the* | **Sub-theme: CHAG non-striking position ensures facilities remain open** | *CHAG Facilities are fully operational in line with their* |
|  | *dedicated services they rendered during the* | MB33, MB73, T2, C1 | *non-strike tradition” Minister of Health (MC31)* |
|  | *strike action”(MA20) (E7)* | *“In line with our convention, inspired by Christian values, ethics and unique identity,* | **Sub-theme: Challenges to CHAG non-striking** |
|  | **Sub-theme: Continuation of services in** | *we are obliged and restraint from embarking upon strike action to resolve matters* | **positionE**MC16 |
|  | **public facilities** | *of such nature. Their decision further contravenes the policy guidelines for house* | *Workers at CHAG facilities asked to stand in solidarity* |
|  | E5, MA22, S3 | *officer training as circulated by our office. Consequently, management finds the* | **Sub-theme: Continuation of services in public** |
|  | *Doctors from Cuba- "government is* | *actions of the House Officers as a breach of these principles and thus undesirable in* | **facilities** |
|  | *bringing in more doctors from Cuba, as* | *our system.” (MB33)* | **MC20, MC21** |
|  | *part of contingency measures to deal with* | **Sub-theme: Challenges to CHAG non-striking position** | *Deploying other professionals: To open up space for* |
|  | *the three-week-old strike by doctors" (E5)* | M33, C1 | *pharmacist clerks to work, but appears contingency* |
|  | *"Government is bringing in more doctors* | *“The Ghana Medical Association should take them (NCHS) on. I amnot amused by* | *plans are not being followed (MC20) (MC21)* |
|  | *from Cuba, as part of contingency* | *the stance taken by the Catholic Church. The principle that they have adopted* |  |
|  | *measures to deal with the three-week-old* | *means they are adopting slave labor in Ghana”. (MB33)* |  |
|  | *strike by doctors"(MA22)* | “The catholic church was saying they give young doctors top-up: that’s arbitrary, it |  |
|  | *“Seeking to put emergency plans in place”* | doesn’t have a symmetry, they can stop any time.” (MB33) |  |
|  | *S3* |  |  |
|  | **Sub-theme: Traditional Healers** | **Sub-theme: CHAG facilities difficulty managing patient load** |  |
|  | MA20, MA22 | MB44, MB72, C1 |  |

|  | “And traditional medicine practitioners for | *“Conditions in private hospitals are challenging due to the overflow of patients.”* |  |
| --- | --- | --- | --- |
|  | the dedicated services they rendered | *(MB44)* |  |
|  | during the strike action” | *“Already there was pressure on us before the Ghana Medical Association strike and* |  |
|  |  | *currently the situation is getting worse day in day out” Peter Yeboah stated.”* |  |
|  |  | *(MB72)* |  |
|  |  | **Sub-theme: Continuation of services in public facilities** |  |
|  |  | **Quasi-government institutions** |  |
|  |  | MB49, MB18, MB70,MB71, MB74, MB46 |  |
|  |  | “Police hospital for instance had to recall staff on leave, while tents have been |  |
|  |  | erected to manage the numbers” (MB46) |  |
|  |  | “At a district level we have asked medical assistants and retired medical doctors to |  |
|  |  | offer a helping hand to patients” (MB49) |  |
|  |  | “The Ministry of Health has not only presented assorted medical supplies but also |  |
|  |  | posted additional nurses and midwives to the Police and 37 Military Hospitals to |  |
|  |  | assist their colleagues to provide adequate and proper healthcare. Similar |  |
|  |  | interventions are being rolled out in other health facilities” |  |
|  |  | Doctors from Cuba: “Government is also retaining all the Cuban doctors who have |  |
|  |  | completed their rotations and are programmed to leave for Cuba anytime soon.” |  |
|  |  | MB77, |  |
|  |  | “Physicians and Medical Assistants are being deployed wherever possible under the |  |
|  |  | supervision of Doctors who are ready and willing to work.” (MB70) |  |
|  |  | **Sub-theme: Sharing of resources** |  |
|  |  | **MB49,** |  |
|  |  | *“GHS had, in the meantime, linked up with the private sector doctors by giving them* |  |
|  |  | *access to public health facilities and encouraging them to bear more of the load of* |  |
|  |  | *public health needs in the capital*.” |  |
| **Theme: Financial** | **Sub-theme: NHIS card holders assured** | **Sub-theme: NHIS card holders assured free service at NHIS facilities** |  |
| **Protection** | **free service at NHIS facilities** | MB18, MB70, MB71, MB76 | **Sub-theme: Challenges with NHIS system** |
|  | **MA14,** | *“NHIS card holders are eligible for treatment in 1000 NHIS0credentialed private and* | MC27 |
|  | **Sub-theme: Challenges with NHIS system** | *faith-based healthcare facilities and community-based health planning services* | NHIS tariffs to increase by 27% |
|  | MA24, MA25, MA26, MA27, MA28, MA29, | *across the country.”* | **Patients turning to traditional healers** |
|  | MA30 | **Sub-theme: Challenges with NHIS system** | MC19 |
|  | *"Five labor groups have embarked on* | MB78, MC22, E54 | *Extend NHIS to cover herbal medicines - Monic Star* |
|  | *strikes this year, they are the Christian* |  | *Center CEO (E39) (MC24)* |

|  | *Health Association, who suspended services to NHIS cardholders” (MA24)*  *"CHAG announced medical facilities will not continue to provide services to NHIS cardholders effective Monday, March 2013. The association said that the NHIA owed its members GH50million as of January 31*  *2013" (MA25)* | *Threaten to take NHI to court: “we would not stop agitating for our institutions to be paid for the services rendered to patients who have paid their premiums and taxes expected to fund their care”.* Rev. Afrifa-Agyekum (MB78)  Private hospital denies patients with NHIS cards during strike due to debt owed to them (E54)  **Sub-theme: Affordability of services in CHAG facilities MC18**  *Expenseof services at CHAG facilities (MC18)* |  |
| --- | --- | --- | --- |
| **Theme: Advocacy** | **Sub-theme: CHAG acting as mediator**  MA14  *“Concerned Clergy Association of Ghana, CCG, is appealing to the leadership of the Ghana Medical Association, to reconsider their intended industrial action slated for*  *the 11th of February 2013* | **Sub-theme: CHAG acting as mediator**  MB72, C1  *“We are all appealing to the government and the GMA to settle the issue amicably because the up and down is delaying and it is not healthy for our country currently” CHAG director stressed.”* |  |
