# Supplementary material for: Public–Private engagement and health systems resilience in times of health worker strikes: a Ghanaian case study
Source: Health Policy Plan. 2024 Mar 18;39(5):469–85. doi: 10.1093/heapol/czae018 (PMC11095267; doi:10.1093/heapol/czae018)
Supplement: czae018_Supp [file czae018_supp.zip › Adapted scoping literature review.pdf]

Study Protocol

Introduction

There is widespread acceptance of the necessity for governments to progress toward universal healthcoverage (UHC). Health has been identified as a critical consequence and determinant of all elements of the 2030 Sustainable Development Goals (SDGs), and UHC is a crucial objective (1). Although several methods for achieving UHC have been described, the goals of UHC remain the same: *"ensuring that all people have access to the effective promotive, preventive, curative, rehabilitative, and palliative health services they require, and that the use of these services does not expose the user to financial hardship"* (2). For this purpose, governments in many nations, such as Ghana, have implemented efforts to achieve UHC (3). For instance, Ghana's 2020 National Health Policy is centered on attaining SDG 3 and emphasizes the interdependent and indispensable role of UHC in achieving the SDGs (4).

A motivated, trained, and equitably distributed health workforce is essential to achieve UHC. Human resources for health (HRH) are one of the six key building blocks of a health system established by the World Health Organization (WHO) and is arguably one of the most essential components of a health system (5). A health worker[7] is any individual who offers health services either through direct contact as clinical workers (doctors, nurses, physiotherapists, occupational therapists) or indirectly as assistants' helpers or laboratory technicians (non-clinical staff) (6). Health worker density is among the most critical factors determining UHC service availability in low and middle-income countries (LMICs) (7). Health service coverage depends extensively on health professionals' availability, accessibility, and competency to provide high-quality, patient-centered care (7). Countries at all levels of socio-economic development face varying degrees of challenges in one of these areas (5).

Noting the importance of HRH, it is of crucial concern for attaining health system goals and, ultimately, the SDGs when health workers go on strike. Health worker strikes are a worldwide phenomenon and are becoming a more common occurrence in LMICs such as Ghana (8). A 'strike[8]' can be defined as *"the collective withholding of labor/services by a category of professionals to extract concessions or benefit"* (9). Strike action might be confined to a single hospital or region, or it can take the shape of national public sector strikes, the latter of which is significant due to the nationwide interruption of health services (10). Though research is undecided as to the impact of health worker strikes on overall mortality (11-14), public sector strikes in LMICs have been found to have significant health systems effects, for example leading to a substantial decrease in in-patient and outpatient services (13,15,16); breakdown in trust between community and health workers (17,18); decreased health worker motivation (17,19), and entrenching inequalities as the effects of health worker strikes are disproportionately felt by the poorest in society (14,17,20).

Important contextual considerations for understanding health worker strikes in LMICs include the systemic inadequacies linked to human resources challenges such as staff shortages and inequitable staff allocation (21-23). Common causes of health worker strike in the public sector include inadequate poor working conditions and the failure of employers (often the government) to comply with collective bargaining agreements (10).

---

[7] For the purposes of this study, we only focused on health workers that have direct contact with patients such as doctors, nur ses, physician assistants and pharmacists.

[8] Unless otherwise states 'strike' as used in this paper refers generally to health worker strikes. We use 'strikes' and 'health worker strikes' interchangeably throughout the paper.

Few studies have examined the experiences and impacts of strikes in LMICs and how health systems respond during strikes (8,17,18). Most literature on strikes has focused on assessing the effect of strikes on mortality (13) and examining the ethical concerns of strikes (17). Studies that have examined responses to strikes in LMIC settings found that the ability of the population to obtain alternative health care in private facilities is vital to ensuring the continuation of services (8,17,18). In many contexts, patients commonly turn to the private sector when public sector health worker strikes occur (17).

However, without system-level interactions with the private sector to better facilitate this coordination, accessing health services in private facilities may have significant financial consequences for poor and vulnerable populations and compromise the quality of care received in these facilities (10,17,18). Thus, governments in these contexts many need to build more formal relationships with the private sector to develop strike response capabilities. Importantly, there is an ever-increasing understanding of the significant role the private sector can play in LMICs in achieving health system strengthening and responsiveness (7,24,25). Due to resource difficulties in LMICs, the public sector's ability to achieve UHC goals alone is significantly undermined. Consequently, cooperation between the public and private sectors has become increasingly important in these contexts (26).

In light of the potential repercussions and ramifications of health worker strikes on the attainment of UHC in Ghana and other LMICs, every effort must be taken to prevent health worker strikes and respond effectively when strikes occur (20). There is a need for more health systems-based research focused on understanding resilience capacities and strategies nations can adopt to cope with strikes. Despite the important role private providers can play in offering health systems resilience during this time, it is of great concern that no study has explored this interaction in any significant detail (17).

Scoping literature review

A scoping literature review was conducted to explore the context of health worker strikes, the engagement between the private and public sector and health system resilience in Ghana and LMICs. The search strategy used is discussed in detail in this protocol's methods section, and the results are presented below.

Health worker strikes

Health worker strikes are a global phenomenon. Between 1996 and 2005, there were approximately 620 reported strikes in the health sector, and almost one-fifth occurred outside of sub-Saharan Africa (SSA) (13). In 2015 alone, health worker strikes were reported in India, Australia, Ghana, Nigeria, Venezuela, and the United States (12). A review of health worker strikes in low-income countries by Russo et al (2020) found that the annual median number of strikes in these settings was six strikes annually (the highest number of strikes were recorded in 2014 and 2018), and the annual number of working days lost to strikes in low-income settings was projected to be 77.5 days (on average, a strike in the health sector was taking place in a low-income country every third working day) (8).

The subject of health workers' right to strike is laden with significant ethical disputes and has been reported on in detail elsewhere (9,10,12,17,27,28). Practitioner strikes generate tension between healthcare personnel's responsibilities to safeguard their patients' health and their rights as employees (12,28). The primary purpose of a strike by an employee is to inflict some loss, usually financial, on an employer to convince them to terminate the impasse in negotiations and concede to the workers' demands (10). In the case of public sector health worker strikes, where the government is often the employer, the financial impact is indirect, and

the strikes' ability to negatively impact patients is more direct (27). Therefore, some patients must suffer harm or inconvenience as a result of a health worker's strike for it to succeed (27)

The impact of strikes on a nation and patients is not immediately catastrophic and is highly context-dependent and determined by several factors, such as the length of the strike, the precise measures taken to maintain a level of service supply during the strike, and the population's ability to acquire alternative care (8,17). In most studies evaluating the impact of health worker strikes on patients and the general population, mortality has served as the primary outcome measure and paints a divided picture. Some studies indicate that mortality rates fall or remain unchanged during strikes (11-13), whereas others indicate an increase in mortality (14,29). However, analyzing the consequences of strikes on patients based purely on mortality provides a very narrow and constrained view of the overall impact of strikes. In most situations, death is an uncommon occurrence that is mostly unaffected by extreme shocks (11). Several of the primary causes of death, for instance, are immune to medical therapy (11). In addition to preventing death, the health system aims to reduce pain and disability and enhance the overall quality of life and well-being (21). The effect of strikes on these objectives are not represented in mortality statistics.

However, consistent across all settings, regardless of income level, is that strikes commonly affect the delivery of health services, with the potential to have significant financial and political ramifications for a nation (8,11,12). In high income settings, massive cancellations of elective surgeries and hospital consultations have resulted from strikes (8,12,30). In 2012, for instance, doctor strikes in the United Kingdom led to a 45.5% reduction in outpatient services (12,30). The effect of strikes in LMIC settings has been argued to be worse (8,13,17). These adverse health system outcomes have been linked to infrastructural challenges, existing resource challenges, already-limited human resource capacity in these settings, weak institutional arrangements, and lack of access to affordable alternative healthcare sources during strikes (8,17).

In SSA, countries that often experience health worker strikes include Ghana, Kenya, Zimbabwe, and Nigeria. These strikes often take the form of national public sector strikes (13). For example, in Kenya in 2017, doctors embarked on a 100-day strike, followed by nurses and shortly after that nurses embarked on a 110-day strike (17,18). In 2015 and 2016 in Ghana, various health professionals, including pharmacists and doctors, embarked on nationwide strikes, including a three-week doctor strike in August 2015 (31). Public sector health worker strikes of this nature often result in the suspension of outpatient services and even emergency services (15).

The impact of health worker strikes in In LMICs is disproportionately felt by the poor, which further entrenches the health system's underlying social inequalities. A sizable proportion of the population in LMICs depends on government-run public services (8,10,17). Even when patients have access to private healthcare facilities, doing so sometimes comes at a considerable cost, further entrenching the poverty of vulnerable individuals who must rely on private healthcare (10,17,18). Furthermore, all health worker strikes reported in these settings affect outpatient services (8). A study by Scanlon et al (2021) revealed that pregnant women in Kenya were significantly less likely to receive sufficient prenatal care, such as vaccinations and antenatal check-ups, during strike periods (18,32).

Strikes may also have unquantifiable harmful consequences on ‘software’[9] components of the health system (17,18,33). The loss of trust between health personnel and the populations they serve is an essential consequence of health worker strikes in LMICs (17,18,33). Trust is vital in health systems because the health

---

[9] The Ortiz Aragon framework is commonly used in health systems to describe the capacities of an organisation. Hardware refers to resources such as infrastructure, technology and finances (105). Software if divided into two components (105). Tangible software refers to capacities such as formal management procedures and processes and intangible software refers to values, norms, power and relationships (105).

system is seen by most as being social and relational (17). The effect of health worker strikes on trust is worrying as trust has been associated with improving patient outcomes such as the adoption of preventative behaviors and adherence to treatment (18).

Recognizing that strikes are a typical occurrence in LMICs with potential negative impacts on the health system, proper planning and preparation must precede any potential crisis (12,15,17,22). Due to the influenceof strikes on health care delivery and other health system dynamics, health worker strikes can be considered a chronic or 'acute' health system stressor or shock. A stressor is a persistent disruption of the health system'soperation, while a shock is an acute disruption (34). Consequently, it is crucial that governments thoroughly understand the dynamics of strikes and establish resilience and response capacities for when they arise.

Health system resilience during health worker strikes

In recent years, the concept of 'health system resilience' has undergone significant modification and discussion. The West African Ebola outbreak and the COVID-19 pandemic have influenced the prioritization of systems resilience in the global agenda (35). Most definitions and concepts of resilience characterize resilience as the ability of the system to absorb shocks, respond to shocks, and emerge from those shocks even stronger than before (36). Kruk et al (2015) define health systems resilience as *"the capacity of health actors and institutions and populations to prepare for and effectively respond to a crisis, maintain core functions when a crisis hits and informed by lessons learned during the crisis, reorganize if conditions require it"* (36).

In addition, 'everyday resilience' as described by Barasa et al (2017) and Gilson et al (2017) emphasizes the need for systems to respond effectively to day-to-day chronic health system difficulties in addition to acute shocks - which were often the focus of health systems resilience literature (34,37). An 'acute shock' is sudden occurrence and transient in nature. 'Chronic stressors' are described as persistent and recurrent challenges over a long period of time (34,37). Broadly there are three types of resilience strategies utilized by health institutions, absorptive, adaptive, and transformative (37). Absorptive strategies refer to strategies that allow the system to continue delivering services without any meaningful change to its original structure (34,38). In contrast adaptive strategies require the system to make some adjustment in response to the shock (34,38). At a point where a system must implore strategies that fundamentally alter its original structure then the strategy is regarded as transformative (34,38).

Notably, resilience can be viewed as both an outcome and an ability of a system (39). As an outcome, resilience is seen as a quantitative measure of the performance of a system in dealing with shocks (40). Health system resilience as an ability, on the other hand, refers to the actions and strategies that are put in place to build and maintain the ability of a health system to withstand and recover from disruption (39). In this study, we focus on resilience as an ability of a system in line with the conceptualization of health systems as socially constructed complex adaptive systems[10] (34,37,39). Thus, in understanding resilience, there is a particular emphasis on understanding what gives the system the ability to absorb, adapt and transform to shocks and stressors (39)- including important effects of software elements in a system (such as power, values) and the interactions between actors (34).

Though health systems resilience has gained prominence on the global health agenda, there is widespread agreement that the term is still primarily theoretical in the field of Health Policy and Systems Research (HPSR). Saulnier et al (2021) report on the findings of the global health system consortium established to identify priority areas for health systems resilience research. One of the critical issues noted was the need for research

[10] We unpack the concept of health systems being conceptualized as complex adaptive systems later (see theoretical underpinning).

on how private providers can contribute to the resilience of health systems in the face of shocks and stressors (41). In health systems resilience literature, it has also been emphasized that there is a need for greater understanding of the types of shocks that health systems confront and how systems respond to specific shocks (as studies have commonly focused on the resilience of the system as a whole) (35).

A scoping review of studies that mention health system resilience during health worker strikes found nine studies that addressed this in some detail (Table 1). These studies show that in some LMIC settings, there are often no system-level mechanisms for responding to and managing strikes (17,33,42,43). Typically, individual health providers and communities undertake resilience methods that are ad hoc, inconsistent, and difficult to sustain (17,33,42,43). The studies that directly analyzed health system resilience during strikes utilized the 'everyday resilience framework' and were primarily focused on Kenya (14,17,20). These studies classify the resilience strategies in Kenya as absorptive and adaptive, meaning that the strategies were short-term and limited (17,18). These studies also found no evidence of 'transformative strategies' and noted that the Kenyan health system was poorly prepared to face future strikes (17,18).

Nearly all studies on health system resilience during health worker strikes noted that access to private health services as an alternative form of care was essential for the continuation of services and resilience of the overall system (13,14,17,20,33,42-44). A study by Ong'ayo et al (2020) in the Lancet which found that mortality rates in Kenya remained constant during strike periods attributed their findings in part to the continuation of services in private facilities (13). A study in Nigeria found that during strikes there was a 66% increase in referrals to private facilities (44).

In addition, the literature indicates that the private sector may not always be able to accommodate the surge of patients (14,17,42). A study by Adam et al (2018) at a faith-based hospital during the 100-day doctor strike in Kenya revealed an increase in hospital mortality (14). The study found that although patients may be redirected to private facilities for care, the lack of effective strategies to manage the patient load may cause these institutions to become overwhelmed, compromising the quality of care received (14).

Some studies have highlighted the need for more system level coordinated strategies between public and private health facilities during strikes (8,14,17,20,42,43). This coordination might facilitate the sharing of resources and the referral of patients during emergency situations, such as strikes. For example, Waithaka et al (2020) found that during the strike period examined in Kenya, some managers adopted informal systems of performing operations in the public sector and then transferring patients to private facilities for post-operative care (17). Some sub-country managers also supplied resources to the private sector to improve their ability to provide services to the community (17). However, these strategies were adopted by individual health providers in Kenya and were not a state approach to the strikes. Waithaka et al (2020) state that further research is required to determine whether and how private providers might provide resilience capacities the public sector can lean on during strikes (17).

Public-private engagements in LMICs

The majority of LMICs have a 'mixed' health system consisting of both a public (state actors) and a private sector (non-state actors), also known as a public/private mix (45). LMICs often face numerous health system challenges, including infrastructural and resource limitations (46). These obstacles make it difficult for the public sector in LMICs to attain UHC and the provision of health care for all (26,47,48). As a result, policymakers are progressively establishing channels for private sector involvement (49). It is well-established that formal engagement and integration of the private sector into the health system in LMICs can help strengthenhealth systems improve resilience (26,48-53).

The WHO defines the private health sector as "*Individuals and organizations that are neither owned nor directly controlled by governments and are involved in provision of health services. It can be classified into subcategories as for profit and not for profit, formal and informal, domestic and international"* (54). The perceptions on private sector involvement in the provision of health services underwent significant transformation in the 1980s and 1990s. Previously, the consensus was that the state was the sole and rightful provider of health services (55,56). However, it became apparent that many LMICs lacked the resources to increase public spending on health, and a sizable portion of individuals in LMICs relied on private sector services, often at a significant financial cost (49). A report by the International Finance Corporation in 2011 found that the private sector was responsible for 50% of health services in Africa (49). The World Health Assembly passed a resolution in 2010, encouraging nations to engage the private sector in delivering basic healthcare services (55).

Understanding the role private providers can play in achieving health system objectives requires understanding the mechanisms by which a government engages private providers (51). Commonly, these mechanisms are known as public-private engagements (PPEs) (26). PPE can be defined as *"deliberate and systematic collaboration, in accordance with national health priorities, between the state and the private health sector"* (45). It is important here to draw a distinction between PPE and public-private partnerships (as these two terms are sometimes used interchangeably in the literature) (56,57). PPE refers to a broad category of different models of collaboration between the state and private sector and is inclusive of public-private partnerships-which represent one model (56,57). The public private partnership is defined as *"long term contract between a private party and the government entity for providing a public service, in which the private party bears significant risk and management responsibility, and remuneration is linked to performance*" (56,57).

Well-designed PPEs have been linked to financial advantages, quality enhancement, and access to health services (53). PPEs can take on many forms and for various purposes (26,45,49,58). Prior research, albeit extremely limited in the context of LMICs, has attempted to describe PPE arrangements in countries, classify the different types of PPEs, and begin to comprehend why some arrangements succeed while others fail (22,24,25,48,59). PPE arrangements have been noted to contribute to LMICs progressing towards UHC (24,25,48,59), assisting with the prevention and management of disease burdens such as TB and HIV (46,60), and responding to human resource challenges (5,22,24). Though these arrangements are in wide existence in SSA, it has been noted that several PPEs fail to meet their objectives and goals (49,57,58). However few studies have analyzed PPE arrangements in Africa and the elements that contribute to their success or failure (45,58).

Whyle and Olivier (2016) developed a typology for classifying PPE arrangements in Southern Africa (58). They identified eight distinct PPE models, including contracting out, voucher programmes, development partnership (DP) regulation, and public-private partnership (PPP). The contracting-out model and public- private partnerships are examples of frequent PPE arrangements discussed in SSA (45,48,58,61). A contracting-out model is the *"delegation of a health-related responsibility by the state to a provider for a price"* (58). This PPE arrangement is characterized by the transfer of duty for services to private suppliers while the public sector retains control over choices on resource allocation. Typically, these agreements have a brief and highly specialized time frame (45,58). In contrast, PPP models are characterized by a long-term relationship, the existence of a contract, a high level of risk sharing, and collaborative relationships. Importantly, the PPE models are influenced by a range of 'software factors' including trust, values and power relationships (58).

Faith-based health providers and PPE

In SSA, faith-based health providers (FBHPs) are one of the most prominent private providers (45,62,63). These providers are typically religiously affiliated and non-profit organizations (64). FBHPs refer to health facilities that provide health services, along with their governing bodies, linking networks, and affiliated entities (45). Twenty years ago, there was a lack of literature on the scope, function, and integration of FBHPs within health systems (62). However, a revived emphasis on the importance of FBHPs in African health systems and the role of religion in development during the past decade has resulted in an increasing body of work on FBHPs (62,65). It is commonly acknowledged that FBHPs play an important and prominent role in the provision of health services in SSA (45,58,62,63,65).

Most FBHPs in SSA are affiliated with the Christian faith. The desire to better coordinate services and negotiate the role of FBHPs in post-independent African states led to the founding of the umbrella organizations referred to as Christian Health Associations (CHAs) (45,59). FBHPs trace their origins to the arrival of Christian health missionaries in Africa during the colonial era (62). Although their aim, function, and activities have evolved, several FBHPs have retained a strong presence post-independence (45). Post-independence many FBHPs gained greater independence from their international denominational authorities, which resulted in a decline in denominational funding over time (45,59,63). This increased the dependence of FBHPs on their governments (63). In the 1960s, there was a shift toward developing national networks to improve the coordination of services and encourage collective participation in governance to facilitate engagements with governments (63). There are currently CHA networks in 23 of Africa's 54 nations, including Ghana, Uganda, Malawi, and Kenya (63). Though the organizational structure and nature of CHAs vary greatly from country to country, with some being loose, poorly integrated networks and others being strong, robust networks, they share some commonalities, such as the objective to improve and better organize collective negotiation with government (63).

Table 1 Relevant studies for scoping review on resilience responses to strikes in LMICs

Source	Title	Country of focus	Focus and/or Methodology	Relevant findings
Waithaka et al (2014)	Prolonged health worker strikes in Kenya perspectives and experiences of frontline health managers and local communities in Kilifi County	Kenya	An embedded research approach was conducted on the Kenyan West coast, where qualitative data was collected through informal observations, reflective meetings and interviews with frontline health managers and local communities.	Showed that frontline managers adopted a range of innovative strategies to maintain services - including collaboration with the private sector. Applies 'everyday resilience' and described these strategies as 'absorptive' and 'adaptive' as strategies were ad-hoc, inconsistent and difficult to sustain. Found limited evidence of 'transformative strategies and preparedness for strikes. Noted that patients commonly turned to the private sector
Scanlon et al (2021)	It was hell in the community: a qualitative study of maternal and child health care during health care worker strikes in Kenya	Kenya	Explores the perspectives of community health workers and nurses during health worker strikes in Kenya through in-depth interviews and focus group discussions	The study applied the 'everyday resilience' lens to evaluate the resilience of the healthcare system during strikes. The results showed that the tactics used during the strikes were primarily temporary and inadequate, lacking in system-level approaches to handle the strikes. The study suggests maintaining partnerships and connections with the private sector as a way to preserve services.
Adam et al (2018)	Pediatric and obstetric outcomes at a faith-based hospital during the 100-day public sector physician strike in Kenya	Kenya	Reports on experiences at AIC-Kijabe Hospital, a not-for-profit, faith-based Kenyan hospital. Routine data on admissions and deaths of newborn and pediatric patients for a period.	The research found that there was a rise in fatalities at the faith-based hospital, showing that the private hospital was unable to handle the increased number of patients due to the strikes, leading to its inability to manage the patient load.
Irimu et al (2018)	Tackling health professionals strikes an essential part of health system strengthening in Kenya	Kenya	Reports on strikes in Kenya between 2010 and 2017	The conclusion of the report states that government's knee-jerk reactions, such as intimidating healthcare workers, are ineffective. The report emphasizes the importance of setting up solid connections with private sector partners and creating risk management plans that are established prior to times of emergency.
Salama, McIsaac and Campbell (2019)	Health worker strikes: a plea for multi-sectoral action	LMICs	Editorial piece on health worker strikes in LMICs	The report underscores the necessity for collaboration across different sectors to effectively prevent disputes and for the involvement of the private sector during strikes.
Nyagetuba and Adam (2019)	Health worker strikes: are we asking the right questions?	LMICs	Commentary on the current nature of strikes in Kenya and other LMIC settings	The report acknowledges that strikes by healthcare workers are a persistent and significant issue that negatively affects the delivery of health services across Africa. It recognizes the impact that strikes have on trust and the motivation of healthcare workers.
Russo et al (2019)	Health workers' strikes in low-income countries: the available evidence	LMICs	A systematic review of grey literature on health worker strikes in lower-income countries from 2005-2018	Presents a framework for evaluating individual strike occurrences, taking into consideration the micro and macro factors that contribute to strikes in lower-income nations.
Ong'ayo et al (2019)	Effect of strikes by health workers on mortality between 2010 and 2016 in Kilifi, Kenya: a population-based cohort analysis	Kenya	Uses daily mortality data obtained from demographic surveillance data to estimate changes in mortality during strike periods from 2010 to 2016.	The report observed that during strikes in Kenya, public hospitals experienced disruptions in service delivery, while services were maintained in private and faith-based hospitals. This was noted to play a role in the overall conclusion that mortality rates remained unchanged between the strike and non-strike periods.
Oleribe et al (2020)	Industrial action by healthcare workers in Nigeria in 2013–2015: an inquiry into causes, consequences and control—a cross-sectional descriptive study	Nigeria	Cross-sectional descriptive survey using self-administered questionnaires.	The study discovered that in Nigeria, healthcare worker strikes resulted in 66% of patients being referred to private healthcare facilities. Unlike other studies, the primary reason for the strikes found for strikes was poor leadership and management.

Whyle and Olivier (2017) contend that Christian Health Health Associations (CHAs) have a distinct public-private engagement (PPE) model with their governments, referred to as the *"CHA-State PPE model" (*Appendix 11) (45). They found that this model embodies characteristics of both contracting out and PPP models. Like contracting out models, the CHA-State model exhibits characteristics of "*duty transfer to CHAs*", a "*hierarchical relationship in which the state retains control"*, and "*restricted resource sharing"* (45). It also mirrors a PPP model in its long-term, collaborative nature and significant risk transfer (45). What is particularly interesting about the CHA-State PPE model is that the relationship between the state and CHAs is usually formalized through a memorandum of understanding (MOU) as opposed to a legal biding contract (45). Currently, 11 of the 23 African states with CHAs have signed an MOU with their respective governments (63). The non-binding nature of these MOUs means that they often are not legally enforceable and rely heavily on trust between both parties to fulfill the respective agreements (58,61). Despite the unique elements displayed by the PPE model, few studies have explored this model and how it interacts with the health system.

Arguably, this unique PPE model influences (or can influence) the role of FBHPs during health worker strikes and is worth exploring in further detail. The CHA-State PPE being based on significant trust and values might position FBHPs as dependable partners during health worker strikes. In the case of health worker strikes, studies have revealed that the poor typically seek assistance from FBHPs as opposed to for-profit providers because these providers are generally perceived to be more affordable (62). In addition, for-profit private providers in some contexts are more prominent in urban regions (62,66,67), but FBHPs are often prominent in rural and semi-urban areas (62,66,67), potentially making them more accessible to the public during strikes. Despite the potential of FBHPs to contribute to health system resilience during strikes, this issue has never been explored.

Public-private engagement in Ghana

As mentioned, health worker strikes have been frequently reported in Ghana (68,69). The second largest private sector in Ghana is the FBHP sector – thought to provide around 20-25% of all health services (66). It is suggested that the relationship between the Christian Health Association of Ghana (CHAG) and the Government of Ghana (GoG) provides resilience capacities to the health system during this time (70,71). Consequently, Ghana provides an interesting case study for examining the interaction of private providers with the state during health worker strikes. The following section will review the Ghanaian health system regarding the private-public mix, the context of health worker strikes in Ghana, and CHAG-state PPE model.

Ghana's health system is a pluralistic health system consisting of public and private providers and Teaching hospitals. The public sector is comprised of two institutions: the Ministry of Health (MOH), which oversees *"policy formulation and resource mobilization"* in Ghana, and the Ghana Health Services (GHS), an independent administrative entity responsible for implementing national policies (66,72).[11] The Ghanaian health system consists of three tiers: tertiary services supplied by Teaching Hospitals[12], secondary level facilities (mostly GHS and private facilities), and primary level institutions (District hospitals, polyclinics, and Community-based Health Planning Services) (73).

---

[11] This separation between the GHS and MOH was as a result of the of the GHS and Teaching Hospitals Act 525 of 1996. The act established the GHS as in independent agency with the mandate of providing primary and secondary health services (73).

[12] Teaching Hospitals are semi-autonomous national referral hospitals tasked with providing tertiary care, research, and training (73).

The Ghanaian health system performs relatively well compared to other countries in SSA. The nation has achieved considerable strides in decreasing newborn and child mortality and nearly doubling the number of assisted deliveries in the past decade (72). As of 2017, the life expectancy in Ghana was 64 years (compared to 53 in other African nations), and the maternal mortality rate was 32.8 deaths per 1000 births (versus the average of 47 in other SSA nations) (72). Despite these efforts, the nation's health system still faces numerous obstacles, such as a double burden of disease characterized by infectious and non-infectious diseases, inequitable access to health care, and the ongoing HRH crisis (72,74,75).

Two major health sector reforms characterize the Ghanaian health system. The first was the introduction of a universal health system in the form of the National Health Insurance System (NHIS)[13], which was established in 2003 and came into effect in 2005 (59,60). At present the scheme covers approximately 60% of the nation and offers an extensive benefits package (covering nearly 95% of the disease burden in Ghana) (74). Public and private providers are part of the scheme, and scheme holders can seek care at any of the accredited facilities (74). Despite studies indicating that the NHIS has increased the utilization of health care services in Ghana and decreased the financial burden of care on patients, the scheme faces several challenges, such as reports of fraud, mismanagement of funds, and lengthy delays in paying providers (46,66). These challenges negatively impact all public and private providers in Ghana (46,66).

In response to the escalating fragmentation of the health system, the second major reform centered on supporting development via a Sector Wide Approach (SWAP) and fostering deeper connections between the public and private sectors (66,76). This emphasis on the need for better PPE led to the creation of the Private Health Sector Policy in 1999 to support expanding and integrating the private sector within the health system (74). The National Health Policy (2020) is founded on this principle. It is based on a whole-of-government, whole-society approach to the delivery of health services - recognizing the fundamental role of the private sector in health system strengthening (72).

Approximately 50-60% of all health services in Ghana are currently provided by the private sector (46,66). For-profit providers had the highest proportion of these services, followed by FBHPs, who are almost exclusively comprised of CHAG-networked facilities (66). For-profit providers are more prevalent in urban areas, whereas CHAG facilities are more prevalent in rural and semi-urban regions (64,66). Although the interaction between the public sector and private providers varies widely, the most common model of engagement between the GoG and the private sector often takes the form of an MOU (more below) (45,46,66,72). Currently, in Ghana, an MOU exists between the public sector and nearly every listed private provider (46).

The Christian Health Association of Ghana

The cooperation between CHAG and the MOH has been cited in many studies as an example of an effective PPE model in Africa (58,63,66). Currently, more than three hundred facilities in Ghana are networked through CHAG (70). CHAG Member institutions (referred to as CMIs) supply a range of curative, preventative, and rehabilitative services, including immunization, health education, maternity and child health, and reproductive health services (77). CHAG was one of the first CHAs established in Africa and has been an integral part of the Ghanaian health system pre- and post-independence. In 2003, CHAG and the MOH signed an MOU to formalize their engagement. The MOU highlights the importance of CHAG to the Ghanaian health system and

[13] The NHIS was created as a policy response to the unpopular "Cash and Carry" system. The model made it compulsory for patients to pay before they could receive care which led to a significant rise in out-of-pocket expenditure in Ghana.

formalizes as an agency within the MOH. The MOU also affirms the autonomy and independence of CHAG, noting that though the government would supply financial other logistical support to CHAG facilities, CHAG would remain an independent agency. The MOU also appoints some CHAG facilities as district hospitals (70,71).[14]

CHAG and the MOH are said to have a strong and trustworthy relationship although there have been times of severe tension. Most of this conflict stemmed from disagreements regarding HRH management (78). There have been multiple attempts and agreements between CHAG and the MOH to manage human-resource-related issues to maintain fairness. One solution adopted in 2000 was the secondment strategy, in which the government would be solely responsible for the payment and staffing of select government employees in CHAG facilities to integrate better the HR systems of both sectors (71,78). However, the secondment approach faced numerous obstacles and has since been amended. Currently, most health workers in CHAG facilities are paid by the MOH, but they are administered and managed by CHAG (70). This modification to the secondment policy also ensures that CHAG employees receive the same terms of employment and benefits as GHS employees- even employment benefits obtained because of strikes.

Health worker strikes in Ghana

Most public sector health worker strikes in Ghana have occurred in the past two decades (69). Few peer-reviewed studies have analyzed the background, characteristics, and effects of strikes in Ghana despite their prevalence. Most information on these strikes is found in media reports on Ghana Web, the Ghana News Agency and other grey literature. (Appendix summarizes the relevant papers and documents on the context of health worker strikes in Ghana).

Health worker strikes have been reported in Ghana in 2005 (79), 2013 (68,69,79,80), 2015 (59,68,69,73,79,81), 2016 (70) and threats of strikes continue today. Strikes occur for various reasons, but the most frequent causes include concerns over low wages, salary disparities between professions and disputes over conditions of service (69). Most recently, in July 2021, several health profession boards threatened to embark on strike action if the government did not agree to pay 20% of their cost-of-living allowance. As shown in Appendix 1, the most reported strike incident in Ghana is the three-week national doctor strike in the public sector which occurred in August 2015. This strike was caused by a long-standing dispute between the Ghana Medical Association (GMA) and the MOH (59,68,69,73,79,81). During the strike, outpatient services at all public facilities ceased, and as the strike escalated, emergency and in-patient services by doctors were also withdrawn (31,82,83).

Health worker strikes often occur in response to and contribute to an already-persistent HRH crisis in Ghana. In 2006, the World Health Organization identified Ghana as one of 57 countries facing a human resource crisis characterized mostly by a shortage of health workers and inequitable distribution of workers (75). Though the nation considerable progress in responding to these challenges, human resource challenges continue to persist (75). According to the Ghana Medium Term Review (2018), the HRH sector is characterized by low wages, poor working conditions, low staff motivation, unequal distribution of health workers, and paradoxical unemployment among some health employees (75). A study by Asamani et al (2020) showed that the GHS had a staffing shortage of 41% (84). Health worker strikes have also been found to sometimes initiate a cascade of policy reforms that can have unintended consequences for the health system (79,84). For example, health

[14] This means that some CHAG facilities function as public sector referral hospitals but are ‘owned’ by CHAG.

worker strikes that took place in 2005-2006 in response to the implementation of an Additional Duty Hours Allowance policy led to a significant increase in government expenditure on health worker salaries (79). Nearly 98% of GoG's allocation of funds to the MOH is spent on health workers' salaries (79).

Similar to strikes in other LMIC contexts, it is frequently documented that public sector health worker strikes in Ghana bring the public health system to a near halt (59,68,69,73,79,81), many leading to the cessation of emergency and outpatient services. Again, like in other LMIC settings, a key element of maintaining the provision of services during strikes is the ability of the population to seek healthcare from private facilities. However, unlike other LMIC settings, the GoG is reported to have some system-level engagements with the private sector to aid with the continuation of services (71). According to media reports during the 2015 strikes, the GoG published a list of quasi-government hospitals that were not on strike and reached out to retired physicians for assistance in government and private institutions (31,85-88).However, redirecting patients to private hospitals does not guarantee that private providers can handle the additional patient load or that the most vulnerable members of the population can afford private hospital care.

What is known about the role of CHAG during health worker strikes?

As previously mentioned, the ability of the population to access health care at private institutions during public sector health worker strikes is essential to the systems resilience. It is argued by some that the close and long-standing relationship between the MOH, and CHAG contributes to the resilience of the Ghanaian health system during health worker strikes (59,70,71). However, the nature of this relationship in the context of health worker strikes in Ghana has never been explored before. A range of factors that are unique to CHAG- state PPE model in Ghana could influence if and how this engagement provides resilience capacities that the Ghanaian health system can use during times of health worker strikes.

In addition to the unique PPE model between CHAG and GoG, it has been argued that the revised secondment policy between CHAG and the MOH has contributed to this interaction during health worker strikes (59). As previously explained, the amendment to the secondment policy has resulted in the majority of workers in CHAG facilities receiving wages from the MOH while being overseen and administered by CHAG (64,75). This amendment also ensures that CHAG personnel receive the same conditions of service and benefits package as GHS workforce, and if this package is modified during a strike by public health workers, these advantages accrue to CHAG staff members as well (75).

Moreover, CHAG facilities reportedly have a stringent non-striking policy that is tied to their religious values, which are said to play a significant role in CHAG's identity (70,71,87). According to Yeboah and Gilbert (2017), this non-striking position helps to offset the harmful effects of strikes on the population by ensuring the continuation of health services (70,78). For example, in September 2016, when pharmacists in the public sector went on strike, the MOH officially and publicly advised patients to seek care in CHAG facilities, recognizing CHAG's non-strike stance (70,78).

Problem statement

Globally, health worker strikes pose a threat to the fundamental human right to the highest level of health and the realization of UHC. The potential negative health system effects of public sector health worker strikes include the avoidable death of some patients, the entrenchment of health system inequalities, the reduction of health worker motivation, and the erosion of health system trust. In LMICs where most of the population

depends on public health services and where public systems face many resource and infrastructural challenges, these effects are often more pronounced. To ensure the continuation of essential services, health systems must build resilience capacities to prepare for and respond to strikes when they occur. During health worker strikes in LMICs, private providers have been identified as crucial to maintaining service continuity. No study has yet examined how the private and public sectors interact during health worker strikes and whether this can contribute to overall health system's resilience.

Ghana has been the site of many public sector-, nationwide health worker strikes over the past two decades. Frequently, these strikes bring the public health system to a halt. According to some, the interaction between CHAG and the GoG provides resilience capabilities to the Ghanaian health system during these times. However, this idea has never been explored in any significant detail. Ghana's complex health system, including the HRH crisis and the unique public-private engagement (PPE) model between CHAG and GoG, may influencethe interaction of the public and private sector during health worker strikes. To unpack the contribution of private providers to resilience capacities in Ghana during health worker strikes, an in-depth examination of the context of strikes in Ghana, the nature of the relationship between the state and private providers, and the unique factors that influence this relationship will be required. The reality is that the threat of health worker strikes in Ghana and other LMICs persists, and the health systems in these countries must plan and prepare for this future possibility to safeguard the right of individuals to the highest quality of health care. Assuch, the following study aims to answer the question:

Does public-private engagement provide health system resilience during public sector health worker strikes in Ghana?

The terms 'public-private engagement', 'health workers' ', strikes', and 'health system resilience' have been operationalized in the scoping literature review above. The table below gives a summary of the terms:

Table 2 Concepts for study

Concept	Definition
Public-private engagement	"Deliberate and systematic collaboration, in accordance with national health priorities, between the state and the private health sector" (45)
Health workers	"A health worker is any individual who offers health services either through direct contact as clinical workers (doctors, nurses, physiotherapists, occupational therapists) or indirectly as assistants' helpers or laboratory technicians (non-clinical staff)" (6).
Strikes	"The collective withholding of labor/services by a category of professionals, for the purpose of extracting concessions or benefits" (9).
Health system resilience	"The capacity of health actors and institutions and populations to prepare for and effectively respond to a crisis, maintain core functions when a crisis hits and informed by lessons learned during the crisis, reorganize if conditions require it" (36)

Methods

Purpose and objectives

To answer the above question, this study will describe the case of the engagement between CHAG (private sector) and the GoG (public sector) during health worker strikes in Ghana from 2010-2016 and explore how this engagement may contribute to health system resilience.

The objectives of the study are:

1. To describe the context of public sector health worker strikes in Ghana from 2010-2016.

2. To describe the role of CHAG in responding to public sector health worker strikes from 2010-2016.
3. To explore the factors influencing the engagement between CHAG and the GoG during health worker strikes from 2010-2016.
4. To explore the ways in which the engagement between CHAG and GoG may have contributed to health system resilience during health worker strikes from 2010-2016.

Sub-study arrangement

This research project is a sub-study of a larger WHO - Alliance for Health Policy and Systems Research (AHPSR)-a funded project entitled "Systems integration towards UHC: Strengthening the collaborative relationship between faith-based non-profit providers and the Ghanaian public health system" (89)' (see Appendix 4 for main study brief). The AHPSR study investigated the historical and present relationship between FBHPs and the Ghanian health system. The findings revealed that the contribution of CHAG to the nation's health system's resilience during health worker strikes was a recurring and underexplored theme (59,78). This study aims to contribute by examining this interaction in greater detail and describing the role of PPE during health worker strikes with the aim of furthering our understanding of how faith-based non-profit health providers can contribute to health system resilience. The researcher will add to the broader AHPSR study by performing a secondary analysis of the interview transcripts collected.

Methodology

A single case study will be conducted, that is primarily qualitative in nature, and flexible in design (see Box 1). Given the iterative and exploratory nature of the study, the precise specifications of a fixed study design will not be suitable for answering the research question (90). This is a macro-level study since it seeks to investigate national-level interactions and strategies that occur during health worker strikes.

Yin (2009) states that case studies are suited for studies that aims to *"understand a complex phenomenon in its real-life context and when comprehension of that phenomenon depends on assessing context"* (91). Each PPE arrangement has its own inter-relational dynamics (58), and each strike incident occurs in an environment

Box 1 Key characteristics of study design

- **Research design**: Single case study in three phases, following an iterative and flexible design: scoping literature, single case study, consolidation and synthesis of results.
- **Purpose**: To *describe* the engagement between CHAG and the GoG during health worker strikes in Ghana from 2010-2016 and to explore how this engagement may have contributed to the strike-related resilience of the health system.
- **Study design**: Macro level retrospective single-case study with the case defined as 'the engagement between CHAG and the GoG during health worker strikes in Ghana'
- **Theoretical Underpinnings**: Study is situated in the field of HPSR research with its relevant theoretical frameworks. Study will draw on theory related to health worker strikes in LMICs, PPE models in LMICs and health system resilience frameworks.
- **Data collection**: Data for the case study will be collected from multiple sources including peer-reviewed literature, grey literature and secondary data analysis of interview transcripts from primary AHPSR project.
- **Data Analysis**: Data analysis for the case will use a qualitative thematic approach where themes will be decided deductively and inductively. Based in the critical realism epistemological framework.

influenced by a variety of macro and micro-level contextual factors (8). Without a comprehensive understanding of context, it is nearly impossible to investigate the engagement between CHAG and the GoG; therefore, the case study design is suitable. Alternative study methods, such as quantitative surveys or cross-

sectional studies, would be unable to capture and investigate the context surrounding health worker strikes and PPE to the same extent as case study designs (92).

In addition, a unique strength of the case study design is its capacity to accommodate a wide range of evidence. This advantage also distinguishes it from another approach that was considered for this study - a historical study. The advantage of a purely historical study is its capacity to examine the 'dead past' when there are almost no relevant actors or current evidence to describe past events (92). In case study designs, theability to integrate a wide range of evidence, such as recent interview transcripts, current documents, and reports, is especially advantageous (92). Moreover, as demonstrated by the scoping review described in the preceding section, information about health worker strikes in Ghana is abundant but primarily exists in unpublished data and is poorly synthesized. This study will incorporate multiple sources of evidence to gain acomprehensive understanding of the context of health worker strikes in Ghana and the interaction between CHAG and GoG during this time.

In case study designs it is important to define the 'case' and 'context' of the study (92,93). The case is defined as 'the engagement between CHAG and the GoG during health worker strikes in Ghana'. This was deemed the 'case' because the phenomenon being studied is 'PPE' during health worker strikes in Ghana,' and CHAG-state engagement represents one private provider in Ghana.

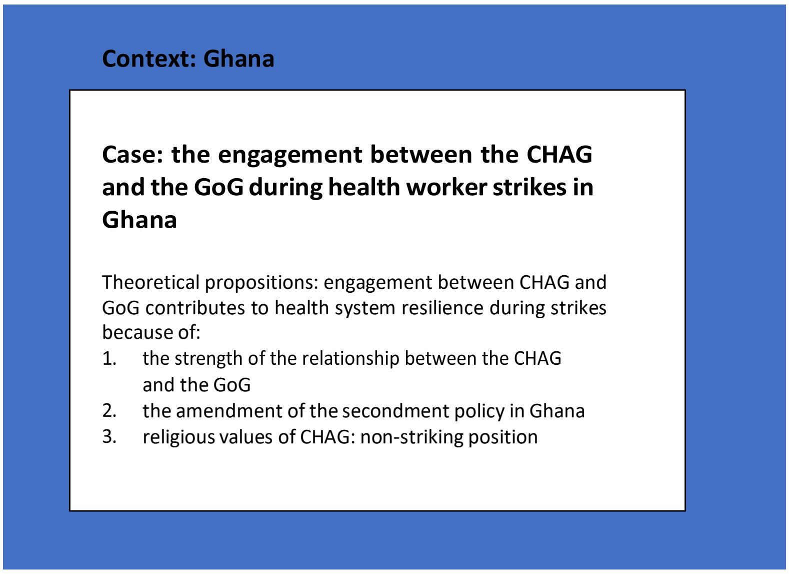

Figure 1 Single-case study approach (source: author adapted from Yin 2014)

The rationale for adopting a single case approach is that CHAG-state PPE represents a critical case. A critical case is a case one where *"theory specifies a clear set of propositions about how explanatory variables affect outcomes, as well as the conditions within which the propositions are believed to be true"* (94). Single case studies are preferable to multiple case studies when a researcher seeks to thoroughly explore these theoretical propositions (94). The theoretical propositions underlying this case (as shown in Figure 1) are that the engagement between CHAG and GoG contributes to the resilience of the health system during health worker strikes because of: 1) The strength of the relationship between CHAG and the GoG, and 2) the amendment of the secondment policy in Ghana, and 3) the distinctive values of CHAG, namely its non-strikingposition. This set of propositions is unquestionably specific to CHAG and GoG relationship. In addition, CHAG is one of Ghana's most prominent private providers. Unlike other providers, they have a long-standing relationship with the MOH that has been maintained through years of cooperation and shared values

(23,25,66). CHAG has become increasingly integrated into the health system, making it a critical case in the context of Ghana (23,25,66). The decision to focus on the period 2010-2016 was influenced by the rapid scoping review, which revealed that this was a particularly turbulent period for the Ghanaian public sector, with frequent strikes by health workers; providing sufficient data for analysis (68,69).[15] In addition this period was encompassed by the broader study which was completed in 2017.

Theoretical underpinnings

Noting that this is the first study of its kind to explore the articulation of the public and private sector during health worker strikes in Ghana, this study will incorporate several theoretical frameworks. Figure 3 shows how these theoretical concepts and frameworks converge in this study to address the objectives of the study.

This study is framed as HPSR and utilizes the theoretical frameworks relevant to this field that emphasizes a systems-based approach to research. Health systems are frequently conceptualized as complex adaptive systems in HPSR (95,96). The WHO identifies six fundamental components of health systems. Although these components are distinct, the system can only be understood through recognizing the interdependence of the different elements and the evolving nature of the health system in response to multiple feedback loops (79,95). Due to the multiple feedback loops, interaction in one area of the system can have unintended consequences for another (79,95). This is particularly pertinent in the case of health worker strikes in Ghana, noting that the HRH interventions have previously led to unintended strike action (79). Health systems can also be understood as consisting of 'hardware,' 'tangible software,' and 'intangible software' dynamics, as exemplified by the Ortiz Aragon framework (95,96). Notably, health systems are intrinsically social systems, and the numerous actors engaged contribute significantly to the complexity of the system (95,96). Actors (individuals, groups, or organizations) have their motivations and ideals and engage with one another through power-influenced social relationships (95,96). These theoretical notions are essential for comprehending the system in which CHAG and the MOH interact and the larger context of health worker strikes.

The first objective of this study is to understand the context of health worker strikes in Ghana in the period 2010-2016. In addition to the theory on health worker strikes in low- and middle-income countries (LMICs) reported in the scoping literature review of this protocol, the conceptual framework for health sector strikes in low-income countries created by Russo et al (2019) (Box 2) will be used to guide the 'rich description of context' (8). The framework's objective is to examine the features, frequency, causes, results, and stakeholders of health worker strikes (8). The framework emphasizes the importance of describing the micro and macro elements of health worker strikes, stressing that these strikes are context-specific (8).

> **Box 2:** Key elements of Russo et al (2020) Conceptual framework for health worker strikes in low income countries
>
> - Framework highlights the need to consider:
>   - The role of influencing actors (and their interactions) such as unions, government, political parties.
>   - Pre-existing economic conditions of a country i.e., economic growth, wage and unemployment levels.
>   - Pre-existing legal conditions i.e. existence of mechanisms for resolution of disputes.
>   - Strike onset characteristics and resolution.
> - Framework highlights the need to understand the interactions and linkages between strike characteristics, pre-existing conditions and actors at both a health sector and country level.

This study's second and third objectives are to investigate the interaction between CHAG and GoG during health worker strikes and the factors that facilitate or impede this interaction. To comprehend the PPE model between CHAG and the GoG, a comprehensive understanding of the public/private mix in Ghana and other LMICs is necessary. As noted earlier, Whyle and Olivier (2017) propose the existence of a unique PPE model between

[15] [i]As our study progressed from the contextual phase, we identified ten strike incidents that occurred in Ghana from 2010-2016. Three of those incidents (described in Part B) had rich information about the interaction of CHAG and the GoG during strikes. We purposively selected these incidents as embedded studies for deeper analysis and cross-comparison. Our analysis focused on these selected embedded units but was not exclusive to these strike incidents.

CHAs and their governments in LMICs, which they refer to as CHAG-state PPE model (23). They contend that this model is distinctive because it combines elements of 'contracting out' models and 'public-private partnerships'. Consequently, this model will provide essential insights for comprehending the nature of CHAG and GoG's relationship and engagement.

The final objective of the study is to investigate how the interaction between CHAG and GoG may contribute to the resilience of the health system during health worker strikes. To investigate and describe this contribution, a comprehension of health system resilience is essential; therefore, theories from the growing body of literature on health system resilience will be utilized. Previous studies assessing resilience during health worker strikes employed the everyday resilience lens. Everyday resilience can be defined as *"the ability of the system to maintain positive adjustment in the context of chronic shocks and stressors in ways that allow the organization to emerge from those conditions strengthened and more resourceful"* (34). Barasa et al (2017) state that the concept of everyday resilience is especially significant in LMICs, where managers of health facilities and systems are constantly confronted with structural and political instability (37).

Using the concept of everyday resilience, Kagwanja et al (2020) develop a conceptual framework that combines two key concepts of resilience thinking: 'resilience capacities' and 'resilience strategies'(38). Cognitive capacities represent the system's capability to understand uncertainty and identify appropriate solutions, while behavioral capacities refer to the system's ability to take action and implement regular or unconventional responses, leveraging learned resourcefulness and readiness (38). Contextual capacities, on the other hand, consist of connections and resources obtained from a mix of strong social capital and extensive resource networks (38). Thus, interactions between CHAG and GoG can confer one or more of these 'resilience capacities' on the system. In contrast, resilience 'strategies' refer to how a system responds to a shock or stressor, which can be classified as absorptive, adaptive, or transformative (as discussed earlier). These strategies may sometimes result in negative or maladaptive health system strategies (34).

The question then becomes how to determine when a resilience strategy during health worker strikes is positive and not maladaptive for the health system. In this regard, Chima (2013) proposes that the objective should be 'ethical strikes' in which effective responses would maintain emergency and essential services and protect individuals from significant financial losses when seeking health care services (10). In addition, Scanlon et al (2021) describe the effects of health worker strikes on vulnerable populations such as pregnant persons. It is thus arguable that ethical strikes would also preserve services for these populations (20). Thus, strategies that achieve these objectives can be viewed as imparting some resilience to the system during strikes.

Phases of study design

The study will be conducted iteratively in three phases with the goal of increasing comprehension from phase to phase (Figure 2).

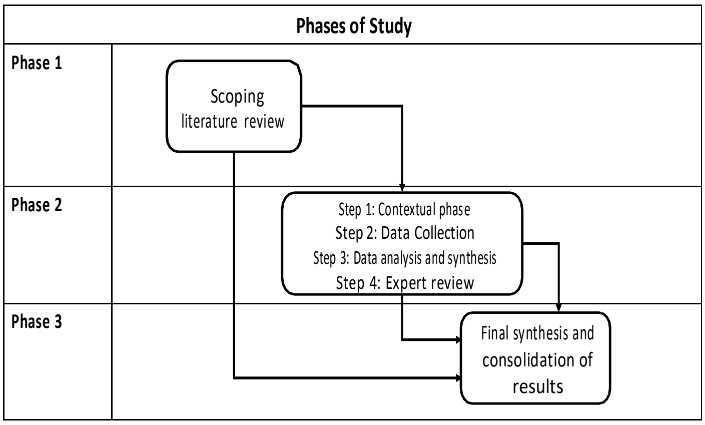

Figure 2 Phases of study *(Source: Author)*

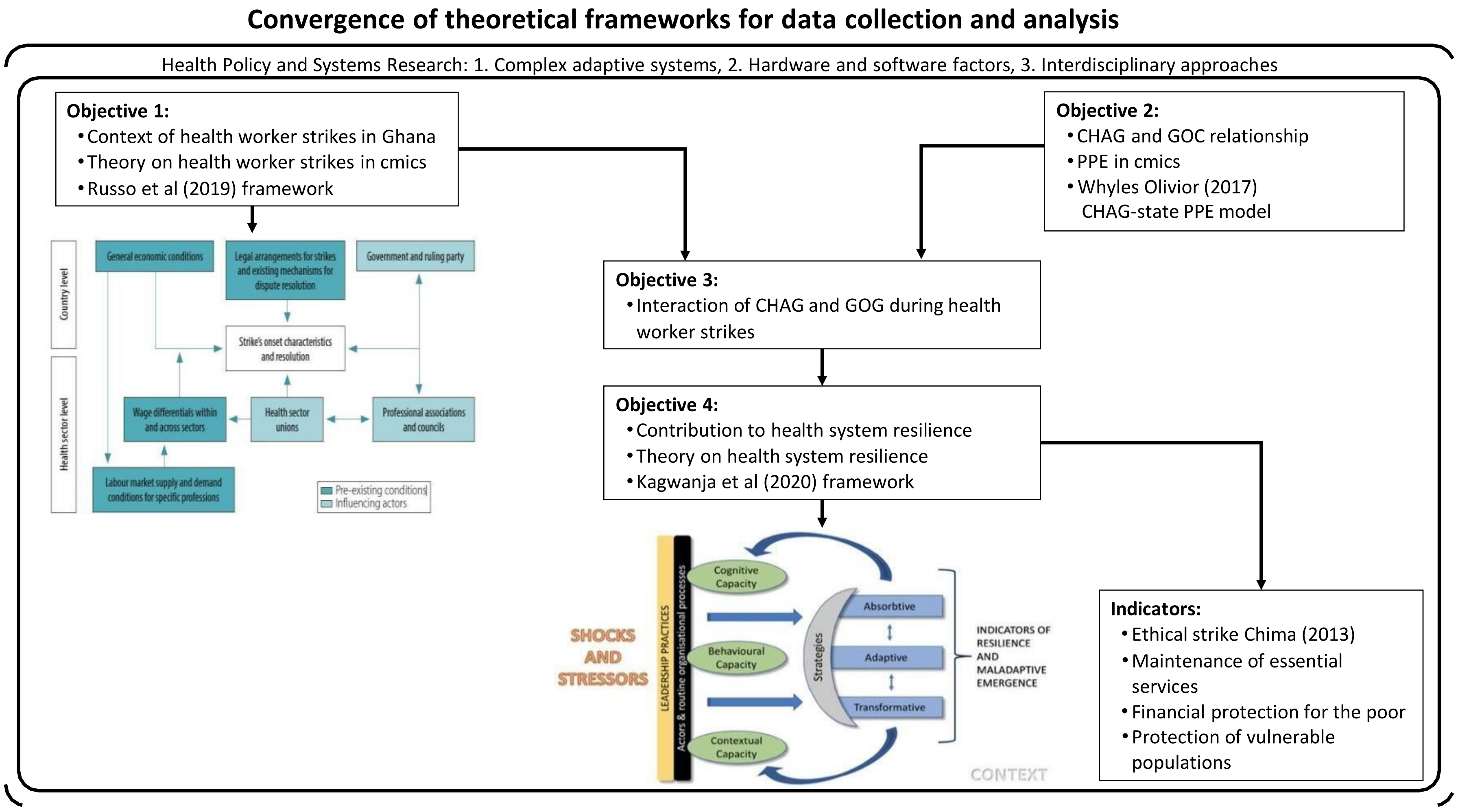

Figure 3 Theoretical frameworks underpinning the study *(source: Author)*

Phase 1: Scoping literature review

At first, the scoping review aimed to identify existing literature on health worker strikes in LMICs (search A).[16] The databases searched included Google Scholar, PubMed, and EBSCOhost. The search period was limited to 2000-2022, although relevant literature that fell outside of the time was also included. Only publications in English were included. Citation tracking was used to identify further pertinent articles by reviewing the included studies' reference lists. A general search on google for grey literature was also performed. The concept of health system resilience came up in studies on health worker strikes in LMICs, and this term was added to the search as well. Important studies relevant to this are summarized in Appendix 1.

After conducting search A, it became apparent that private providers were particularly important during times of health worker strikes, and their role could not be understood without further examination of the public-private mix in LMICs. A second search (search B) was conducted to understand the public-private mix in LMIC health systems. Results from search A and search B informed the decision to focus on health worker strikes in Ghana because of the interesting and unique PPE frequently reported in the nation. There was then a need to conduct a further search on the context of health worker strikes in Ghana. Relevant studies for search C are summarized in Appendix 1.

Table 3 Main search terms and relevant variables for search A

Cluster	Variations
Health worker strikes	'doctor strikes', 'medical professional protests', 'nurse strikes', 'disruptions', 'health worker disputes', 'unrest'
Low-middle income countries	'sub-Saharan Africa', 'developing'
Health System Resilience	'responsiveness', 'preparedness'

Table 4 Main search terms and relevant variations for search B

Cluster	Variations
Public-private mix	'public-private partnerships', 'private sector', 'interactions', 'relationships'
Health systems	'health policy', 'HPSR', 'public health'

Table 5 Main search terms and relevant variations for search C

Cluster	Variations
Health worker strikes in Ghana	'doctor protest Ghana', 'nurse protest Ghana', 'worker unrest', 'human resources for health'
PPPE Ghana	'faith-based providers', 'private providers', 'NGOs'

Phase 2: Single case study of 'engagement between CHAG and GoG during health worker strikes in Ghana from 2010-2016'

Stage 1: Contextual analysis

This phase will begin with a contextual analysis of the case by synthesizing information gathered in the scoping review, with the goals of 1) describing the context of the Ghanaian health system between 2010 and 2016,

[16] In this MPH minor dissertation style, this section of the Methods is written in the past tense, as it has already been conducted at this stage, and has been reported earlier in the Literature Review section of the protocol

importantly the private-public mix, and 2) describing the larger history and context of health worker strikes in Ghana. Detailed descriptions of context facilitate rigor in case studies (92); therefore, it is essential to situate the case within the broader context of health worker strikes and PPE in Ghana and LMICs. The framework by Russo et al (2019) (Box 2) for health worker strikes in low-middle income countries will be used to identify critical themes such as micro and macro contextual features of strikes. to facilitate reporting and analysis of the context.

Stage 2: Data collection

Once the case context is fully understood, data pertinent to the case will be gathered utilizing multiple sources of evidence (Table 6) such as media archives, grey literature, and peer-reviewed journal articles. In addition, we will conduct secondary data analysis of interview transcripts from the primary study. As shown in Table 6, nearly all evidence sources are currently accessible, albeit poorly synthesized, and a significant objective of this study will consist of synthesizing and integrating the various evidence sources. Noting that this study makes use exclusively of secondary resources an additional expert verification process (discussed below) will be conducted to enhance rigor.

Table 6 Data collection sources

Type	Name	Data Type, Source/Location and Availability
Secondary Literature (Peer-reviewed journal articles)	• When 'solutions' of yesterday become problems of today': crisis-ridden decision-making in a complex adaptive system (CAS)- the Additional Duty Hours Allowance in Ghana<br>• An evaluation of the effects of industrial unrest at the Korle-bu Teaching Hospital (KBTH), Accra, Ghana<br>• Ghana's numerous worker strikes; a cause of concern<br>• The imperative of an evidence-based health workforce planning in implementation: lessons from nurses and midwives' unemployment crisis in Ghana.<br>• Single Spine Pay Policy, Can Ignorance delay the benefits it has on the Ghanaian public service worker<br>• Towards universal health coverage: a mixed-method study mapping the development of the faith-based non-profit sector in the Ghanaian health system.<br>• The evolving partnership between the Government of Ghana and national faith-based health providers: leadership perspective and experiences from CHAG	Qualitative<br>Openly available published peer-reviewed articles: Google, Google Scholar, PubMed, EBSCOhost<br>Have (will add on)
Reviews	• Literature Review: Whyle EB, Olivier J. Models of public-private engagement for health services delivery and financing in Southern Africa: a systematic review. Health Policy Plan 2016;31(10):1515-1529.<br>• Whyle E, Olivier J. Models of engagement between the state and the faith sector in sub-Saharan Africa–a systematic review. Development in Practice 2017;27(5):684-697.	Qualitative<br>Openly available<br>Have (will add on)
Media Archives	• Ghana Web Articles<br>• Ghana News Agency Articles<br>• Mojo Online	Qualitative<br>Openly available<br>Have (will explore further)
Institutional websites	• CHAG Website<br>• https://chag.org.gh/<br>• Ministry of Health Ghana<br>• https://www.moh.gov.gh/<br>• Ghana Medical Association<br>• https://ghanamedassoc.org/<br>• The Ghana Registered Nurse and Midwives Association	Qualitative<br>Openly available<br>Have (will explore further)

	• https://ghananurses.org/	
Government briefings and press statements	• Official government briefings and communications on social media<br>• Memorandum of understanding and draft condition of agreements.<br>• Press statements.	Qualitative<br>Openly available<br>Have (will explore further)
Social Media Pages	• CHAG Facebook page<br>• Ghana Medical Association Facebook Page<br>• News Agency Facebook pages	Qualitative<br>Openly available<br>Have (will explore further)
Thesis	• Towards Universal Health Coverage: Mapping the Development of the Faith-Based Non-Profit Sector in the Ghanaian health system.<br>• Partnerships that support health systems resilience over time a study of non-state, faith-based health providers in Africa.	Qualitative<br>Available on UCT Open Access and Google Scholar<br>Have (will add on)
*Secondary Data	Interview transcript from primary study	Qualitative<br>Can gain access to

In addition to the data collected in the preceding table, secondary data from the broader APHSR study will also be analyzed. This consists of transcriptions of interviews conducted with CHAG officials in which the role of CHAG during health worker strikes was discussed. The information from these interviews will also be used for triangulation of data.

Stage 3: Data analysis

During this stage, three questions will guide the analysis and interpretation of the data gathered in the preceding phase. 1) What role did CHAG play during health worker strikes in Ghana between 2010 and 2016? 2) What was the engagement between CHAG and GoG health worker strikes in Ghana from 2010-2016? 3) Did this engagement contribute to the resilience of the health system during this time? The data collected in the preceding phase will be examined to determine its relevance to these questions to create the final 'data set' from which data will be analyzed.

As our data is based on secondary sources of information and documents, the analysis of the documents themselves is crucial to this study. To analyze the documents collected we will implore elements of qualitative document analysis approaches (97,98). The goal of qualitative document analysis is to understand the perspectives, experiences, and meanings that are expressed in the text and how these relate to the research question being studied (97). Gibson and Brown (2011) advise that when analyzing documents for the first time, seeking to understand the time the document was created, the author, purpose, audience and perspective is essential (98).

The data will be analyzed using a thematic analysis method which is a technique for *"identifying, analyzing, and reporting patterns (themes) within data"* (99). Thematic analysis is especially useful as our study will seek to synthesize data from multiple sources (99). In this method, determining what constitutes a theme is crucial. Importantly, a theme is not necessarily determined by the frequency with which it appears in the data set but rather by whether it captures something crucial in relation to the overall research question. What constitutes a theme; will be decided on deductively and inductively (98). Deductive thematic analysis will be guided by the theoretical foundations established in the scoping review. Noting that this is an exploratory study of a topic that has not previously been studied before, it is also essential that the study methodology allows for new themes to emerge from the data.

The scoping review reported on in phase 1 of this protocol established the theoretical foundation that will guide and inform the interpretation of the findings. As noted previously and shown in Figure 1, three important theoretical propositions will be examined for this case. In addition, the interaction between CHAG and GoG

could contribute to the resilience of the health system by conferring specific resilience capacities (cognitive, behavioral, or contextual), which can lead to specific strategies (absorptive, transformative, or adaptive). These theoretical propositions (shown in Figure 3) will form the initial themes that will guide the interpretation of data. However, as this is study is an initial exploration into an understudied topic, it is very possible that new themes will emerge from the data and current themes may need to be adapted. Thus, themes will be decided both inductively and deductively.

Our approach to thematic analysis will be based in the critical realism epistemological paradigm. This paradigm is based on the perspective that there exists a world independent of our perceptions, but that our access to this world is mediated by our cognitive and social structures (100,101). This paradigm highlights the significance of comprehending the relationship between our knowledge of the world and its social and historical context. It seen as the middle position between positivism and relativism (100,101). Similar to positivism the paradigm seeks to comprehend the causal mechanisms that influences a phenomenon, but critical realism does not see these causal mechanisms as an objective reality that can always be measured (101). Like relativism this perspective also seeks to understand how actors make sense of their experience and the influence this has on their perspective of reality. In this study we are seeking to understand public and private sector engagement during health worker strikes. Thus, our focus is understanding the mechanisms that can explain this interaction i.e., the context of the Ghanaian health system.

Stage 4: Expert checking

As this case study relies primarily on publicly available data and secondary data analysis, it is crucial that the interpretation and conclusions reached be reviewed by experts to improve the trustworthiness of the study. Experts are individuals who are not directly involved in the research study but have specialized knowledge of the research field and can provide an informed and objective assessment of the project's methods and findings. Checking of study findings by experts can provide invaluable insights into a study, provide additional resources, and shed light on areas or topics that may require additional exploration. Expert reviews are also beneficial for counteracting the subjectivity that is inherent in all qualitative research. A few (2-3) experts from CHAG and the MOH will be contacted based on their expertise and familiarity with the topic, and involvement in the previous study. The checking process will either involve sharing of initial analysis (written), followed by an electronic phone or Teams call, or an email exchange.[17] These experts were previously expert participants in the broader study for which this is a sub-study. Their involvement then included the signing of a consent form (the conditions of which still cover the information, as they agreed to the continued checking of subsequent research outputs, with no time limit imposed).

Phase 3: Synthesis and write up

During this stage, the feedback from the experts will be incorporated and used to correct any errors in the study or to investigate specific themes in greater depth. The final analysis and expert feedback will be consolidated and synthesized to produce a comprehensive report on how private-public engagement in Ghana can contribute to the health system's resilience during health worker strikes.

Rigor

Due to the nature of qualitative work and case-study design, the researchers will ensure rigor in all phases of the research. Vital to achieving rigor is the formulation of this detailed study protocol that outlines and clearly

[17] No new primary data will be collected from these experts – their role is to check the analytics emerging from this study

defines the case being studied, the reasons for selecting the case, and the underlying propositions and assumptions that inform the case (91,92). Generally, the rigor of case study research is also improved by ensuring complete and detailed reporting of all data collection and analysis methods so that the reader can determine whether the analysis and interpretations are credible (91,92). To ensure complete reporting of results, the researcher will take the necessary steps, including maintaining an active and detailed evidence trail for all collected data. A database of all media reports, grey literature, and secondary literature collected will be maintained and will be made accessible alongside the study's final report. This database will also seek to establish a transparent chain of evidence.

In addition, rigor in case study design, particularly for single-case studies like this one, necessitates a detailed description of context. and comprehensive case contextualization (92,93). Consequently, this study's first and second phases are concentrated on providing an in-depth description of the context of health worker strikes in Ghana, CHAG and state PPE, and the larger context of health worker strikes and PPE models in LMICs. It has also been noted that theory is essential for ensuring rigor by enhancing transferability and confirmability (92,93). As described in detail above, this study is based on multiple theoretical foundations that will be explored iteratively throughout the study. Theoretical principles will direct the collection of data and analysis of data.

Noting that most data collected for this study will be from unpublished sources such as media reports, institutional databases, media statements, student theses and online reports, it is essential to conduct continuous quality checks on materials, and also collect data from multiple sources to avoid bias (98). These data sources will be triangulated with other forms of evidence, such as peer-reviewed literature and secondary data analysis of interview transcripts, to enhance rigor. In addition, the final conclusions and interpretations of this study will be reviewed by experts prior to publication.

Furthermore, all phases of qualitative studies are susceptible to researcher bias, as the researcher has a substantial impact on study design, data collection, and result interpretation (92,93). Noting this, researcher reflexivity is always important. The researcher identifies as a Ghanaian-South African woman of color. The researcher has a background in medicine and is currently a student of public health; consequently, she possesses certain ideological biases that will unavoidably affect the research. Importantly in the context of this study (in which the case is a Christian FBHP) the researcher aligns with Christian values. The researcher will aim to remain reflexive by constantly evaluating their positionality in relation to the study and engaging in frequent checkpoints with the study's supervisor. The study also seeks to reduce the influence of bias by employing expert reviews to assess the validity of the results.

Ethics

Throughout this study, all research ethical principles and practices will be adhered to in accordance with the Helsinki Declaration (102) and international, national, and institutional standards. Ethical approval will be sought from the Human Research Ethics Committee (HREC) of the University of Cape Town (UCT). The ethical risk is deemed minimal. This study will abide by the ethical clearance that has already been granted for the main AHPSR study. This clearance was obtained from the WHO Research Ethics Committee, the Ghana Health Service Review Committee, and the UCT HREC (see Appendix 4).

Noting that this study uses secondary interview transcripts from the primary study, all ethical principles were adhered to in the primary study. Participants were informed of the study's purpose, the risks associated with participation, and their right to withdraw at any time. No participant was compensated for their participation

in the study. The interviews were conducted in confidence and were recorded for transcription purposes. The transcription notes have been made anonymous. In the primary study, ethical principles regarding data handling were also adhered to. All data were stored and backed up on a password-protected drive, and it will be deleted three years after the conclusion of the study. On receipt of the interview transcripts for this sub-study, the same ethical principles will continue to govern data management. Once utilized, the researcher will delete the interview transcripts provided.

This research also utilizes expert analysis to validate its findings. It's important to note that experts are not considered as participants in the study since data collection and examination will be completed before their involvement (103). The expert review process only involves evaluating the study's existing results and interpretations and not collecting new information from the experts. Before being provided with the data for review, the experts will be required to sign a confidentiality agreement (Appendix 2). This is a completely voluntary process, and experts will be informed that they may opt-out at any time. Experts can provide feedback digitally or through a phone conversation, depending on their preference. In the case of telephone conversations, however, experts' words will not be recorded or taken down verbatim.

Risks and benefits

In addition to the ethical risks and considerations discussed previously, the following (albeit minimal and unlikely) risks associated with conducting health system research will be considered.

HPSR studies can present unique risks, which the author will consider throughout the study. Occasionally, HPSR studies can have unintended effects on the health system (104). This study has the potential to unintentionally undermine the function of FHBPs in the Ghanaian health system. The relationship between CHAG and the GoG is constantly evolving and can at times become tense (70). In addition, health worker strikesin Ghana is highly politicized (69), with numerous ethical disputes regarding their moral correctness. Thus, theauthor will ensure accurate reporting and interpretation of the results and conclusions. Importantly, this study will make no moral judgments about health worker strikes in Ghana. The focus of this study is not whether CHAG health workers should join in public sector strikes, rather, it is a retrospective study on the documentedrole of private providers during health worker strikes and the implications of this for health system resilience.Before publication, the author will also present the results and findings to experts to confirm the interpretation of the results and ensure that the way results are being communicated will not pose a risk to the health system.

The research aims to benefit policymakers in Ghana and other LMICs by examining the role private providers can play during health worker strikes and how these capacities may be utilized to ensure the continuation of essential health services. This is the first study of its kind, and it will also address areas where there is a dearth of literature, such as providing a comprehensive contextual synthesis of the history and context of health worker strikes in Ghana. In addition, policymakers interested in the factors influencing the relationship between public and private providers in Ghana and other LMICs will find this study useful. It will also contribute to the much-needed literature on FBHPs and their effect on the resilience of health systems, as well as to our comprehension of the CHA-State PPE.

Communication of findings

This research study will be submitted to the University of Cape Town as a thesis in partial fulfilment of the Master of Public Health degree requirements. Thus, the thesis will be accessible via the open database of

theses at UCT. The final output of this study is a journal article which will be submitted to peer-reviewed journals. Journals with a particular focus on health systems and equity in health service provision will be targeted.

Additionally, research findings will be disseminated to stakeholders. The final journal article will be distributed to the reviewers of the study, as well as to CHAG and the MOH. This study will produce a summary of the context of health worker strikes in Ghana from 2010 to 2016. This contextual analysis will also be provided to officials at the MOH in Ghana. The author will also utilize platforms they are part of, such as the Mandela Rhodes Foundation and the International Working Group for Health System Strengthening, to disseminate the final journal article and findings.

Timeline

The expected timeline of this study is as follows:

Table 7 Timeline for study

Activity	May 2022	Jun 2022	Jul 2022	Aug 2022	Sep 2022	Oct 2022	Nov 2022	Dec 2022	Jan 2023
Phase 1: Scoping literature review									
Draft protocol									
Final protocol submitted to ethics									
Phase 2: data collection and analysis									
Expert reviews									
Phase 3: Consolidation and synthesis of results									

References

(1) Tangcharoensathien V, Mills A, Palu T. Accelerating health equity: the key role of universal health coverage in the Sustainable Development Goals. BMC Med. 2015;13(1):1-5.

(2) World Health Organization. Health in 2015: from MDGs, millennium development goals to SDGs, sustainable development goals. 1st ed. Geneva, Switzerland: World Health Organization. 2015.

(3) Reich MR, Harris J, Ikegami N, Maeda A, Cashin C, Araujo EC, et al. Moving towards universal health coverage: lessons from 11 country studies. Lancet. 2016;387(10020):811-816.

(4) Ministry of Health Ghana. National Health Policy: Ensuring healthy lives for all (REVISED EDITION). Ministry of Health 2020 Jan,:5-15.

(5) World Health Organization. Global strategy on human resources for health: workforce 2030. World Health Organization 2016:11-16.

(6) Joseph B, Joseph M. The health of the healthcare workers. Indian J. Occup Health. 2016;20(2):71.

(7) Reid M, Gupta R, Roberts G, Goosby E, Wesson P. Achieving Universal Health Coverage (UHC): Dominance analysis across 183 countries highlights importance of strengthening health workforce. PLoS One. 2020;15(3):e0229666.

(8) Russo G, Xu L, McIsaac M, Matsika-Claquin MD, Dhillon I, McPake B, et al. Health workers' strikes in low-income countries: the available evidence. Bull World Health Organ. 2019;97(7):460.

(9) Wolfe S. Strikes by health workers: a look at the concept, ethics, and impacts. Am J Public Health. 1979;69(5):431-433.

(10) Chima SC. Global medicine: Is it ethical or morally justifiable for doctors and other healthcare workers to go on strike? BMC Med. Ethics. 2013;14(1):1-10.

(11) Cunningham SA, Mitchell K, Narayan KV, Yusuf S. Doctors' strikes and mortality: a review. Soc Sci Med. 2008;67(11):1784-1788.

(12) Metcalfe D, Chowdhury R, Salim A. What are the consequences when doctors strike? BMJ. 2015;351.

(13) Ong'ayo G, Ooko M, Wang'ondu R, Bottomley C, Nyaguara A, Tsofa BK, et al. Effect of strikes by health workers on mortality between 2010 and 2016 in Kilifi, Kenya: a population-based cohort analysis. Lanc Glob Health. 2019;7(7):e961-e967.

(14) Adam MB, Muma S, Modi JA, Steere M, Cook N, Ellis W, et al. Paediatric and obstetric outcomes at a faith-based hospital during the 100-day public sector physician strike in Kenya. BMJ Glob Health. 2018;3(2):e000665.

(15) Nyagetuba JM, Adam MB. Health worker strikes: are we asking the right questions? Lanc Glob Health. 2019;7(7):e831-e832.

(16) Oleribe OO, Ezieme IP, Oladipo O, Akinola EP, Udofia D, Taylor-Robinson SD. Industrial action by healthcare workers in Nigeria in 2013–2015: an inquiry into causes, consequences and control—a cross-sectional descriptive study. Hum Resour Health. 2016;14(1):1-10.

(17) Waithaka D, Kagwanja N, Nzinga J, Tsofa B, Leli H, Mataza C, et al. Prolonged health worker strikes in Kenya-perspectives and experiences of frontline health managers and local communities in Kilifi County. Int J Equity Health. 2020;19(1):1-15.

(18) Scanlon ML, Maldonado LY, Ikemeri JE, Jumah A, Anusu G, Chelagat S, et al. 'It was hell in the community': a qualitative study of maternal and child health care during health care worker strikes in Kenya. Int J Equity Health. 2021;20(1):1-12.

(19) Alhassan RK, Spieker N, van Ostenberg P, Ogink A, Nketiah-Amponsah E, de Wit TFR. Association between health worker motivation and healthcare quality efforts in Ghana. Hum Resour Health. 2013;11(1):1-11.

(20) Scanlon ML, Maldonado LY, Ikemeri JE, Jumah A, Anusu G, Bone JN, et al. A retrospective study of the impact of health worker strikes on maternal and child health care utilization in western Kenya. BMC Health Serv Res. 2021;21(1):1-7.

(21) World Health Organisation. Universal Health Coverage. 2023; Available at: https://www.who.int/health-topics/universal-health-coverage#tab=tab_1. Accessed 3 Aug, 2022.

(22) Hongoro C, McPake B. How to bridge the gap in human resources for health. Lancet. 2004;364(9443):1451-1456.

(23) Chen L, Evans T, Anand S, Boufford JI, Brown H, Chowdhury M, et al. Human resources for health: overcoming the crisis. Lancet. 2004;364(9449):1984-1990.

(24) Ravishankar N, Dunworth A, O'Hanlon B, Wawire S. Private capacity, public payment: private business participation in government initiatives to improve access to critical health services. BEAM Exchange. 2016 Jun,:19-39.

(25) McPake B, Hanson K. Managing the public–private mix to achieve universal health coverage. Lancet. 2016;388(10044):622-630.

(26) Suchman L, Hart E, Montagu D. Public–private partnerships in practice: collaborating to improve health finance policy in Ghana and Kenya. Health Policy Plan. 2018;33(7):777-785.

(27) Thompson SL, Salmon JW. Strikes by physicians: a historical perspective toward an ethical evaluation. Int J of Health Serv. 2006;36(2):331-354.

(28) Manthous CA. Labor unions in medicine: the intersection of patient advocacy and self-advocacy. Med Care. 2014;52(5):387-392.

(29) Bhuiyan M, Machowski A. Impact of 20-day strike in Polokwane hospital (18 August-6 September 2010). SAMJ. 2012;102(9):755-756.

(30) Ruiz M, Bottle A, Aylin P. A retrospective study of the impact of the doctors' strike in England on 21 June 2012. J R Soc Med. 2013;106(9):362-369.

(31) Ghana Web. Doctors' Strike: Out-Patient Departments empty (Photos). 2015; Available at: https://www.ghanaweb.com/GhanaHomePage/NewsArchive/Doctors-Strike-Out-Patient-Departments-empty-Photos-373860. Accessed Aug 7, 2022.

(32) Scanlon ML, Maldonado LY, Ikemeri JE, Jumah A, Anusu G, Bone JN, et al. A retrospective study of the impact of health worker strikes on maternal and child health care utilization in western Kenya. BMC Health Serv. 2021;21:1-7.

(33) Nyagetuba JM, Adam MB. Health worker strikes: are we asking the right questions? Lancet Glob Health. 2019;7(7):e831-e832.

(34) Gilson L, Barasa E, Nxumalo N, Cleary S, Goudge J, Molyneux S, et al. Everyday resilience in district health systems: emerging insights from the front lines in Kenya and South Africa. BMJ Glob Health. 2017;2(2):e000224.

(35) Ismail SA, Bell S, Chalabi Z, Fouad FM, Mechler R, Tomoaia-Cotisel A, et al. Conceptualising and assessing health system resilience to shocks: a cross-disciplinary view. Wellcom Open Res. 2022;7:151.

(36) Kruk ME, Myers M, Varpilah ST, Dahn BT. What is a resilient health system? Lessons from Ebola. Lancet. 2015;385(9980):1910-1912.

(37) Barasa EW, Cloete K, Gilson L. From bouncing back, to nurturing emergence: reframing the concept of resilience in health systems strengthening. Health Policy Plan. 2017;32(suppl_3):iii91-iii94.

(38) Kagwanja N, Waithaka D, Nzinga J, Tsofa B, Boga M, Leli H, et al. Shocks, stress and everyday health system resilience: experiences from the Kenyan coast. Health Policy Plan. 2020;35(5):522-535.

(39) Biddle L, Wahedi K, Bozorgmehr K. Health system resilience: a literature review of empirical research. Health Policy Plan. 2020;35(8):1084-1109.

(40) Crowe S, Vasilakis C, Skeen A, Storr P, Grove P, Gallivan S, et al. Examining the feasibility of using a modelling tool to assess resilience across a health-care system and assist with decisions concerning service reconfiguration. J Oper Res Soc. 2014;65(10):1522-1532.

(41) Saulnier DD, Blanchet K, Canila C, Muñoz DC, Dal Zennaro L, de Savigny D, et al. A health systems resilience research agenda: moving from concept to practice. BMJ Glob Health. 2021;6(8):e006779.

(42) Salama P, McIsaac M, Campbell J. Health workers' strikes: a plea for multisectoral action. Bull World Health Organ. 2019;97(7):443.

(43) Irimu G, Ogero M, Mbevi G, Kariuki C, Gathara D, Akech S, et al. Tackling health professionals' strikes: an essential part of health system strengthening in Kenya. BMJ Glob Health. 2018;3(6):e001136.

(44) Oleribe OO, Ezieme IP, Oladipo O, Akinola EP, Udofia D, Taylor-Robinson SD. Industrial action by healthcare workers in Nigeria in 2013–2015: an inquiry into causes, consequences and control—a cross-sectional descriptive study. Hum Resour Health. 2016;14(1):1-10.

(45) Whyle E, Olivier J. Models of engagement between the state and the faith sector in sub-Saharan Africa–a systematic review. Dev Pract. 2017;27(5):684-697.

(46) Amo-Adjei J. Conforming to partnership values: a qualitative case study of public–private mix for TB control in Ghana. Glob Health Action. 2016;9(1):28000.

(47) Stallworthy G, Boahene K, Ohiri K, Pamba A, Knezovich J. Roundtable discussion: what is the future role of the private sector in health? Global Health. 2014;10(1):1-5.

(48) Mills A, Brugha R, Hanson K, McPake B. What can be done about the private health sector in low-income countries? Bull World Health Organ. 2002;80:325-330.

(49) International Finance Corporation. How Governments Can Engage the Private Sector to Improve Health In Africa. World Bank 2011:9-32.

(50) Kernaghan K. Partnership and public administration: conceptual and practical considerations. Can Public Adm. 1993;36(1):57-76.

(51) Birungi H, Mugisha F, Nsabagasani X, Okuonzi S, Jeppsson A. The policy on public-private mix in the Ugandan health sector: catching up with reality. Health Policy Plan 2001:80-87.

(52) Basu S, Andrews J, Kishore S, Panjabi R, Stuckler D. Comparative performance of private and public healthcare systems in low- and middle-income countries: a systematic review. PLoS Med. 2012;9(6):e1001244.

(53) Public private partnerships: systematic review of available models for improving health care services. 5th Annual International Conference on Public Administration and Development Alternatives; Oct 7, 2020; Virtual Conference: International Conference on Public Administration and Development. 2020.

(54) Klinton J. The private health sector: an operational definition. World Health Organization. 2020:2-4.

(55) Buso DL. Public-Private health sector mix-way forward. S Afr Fam Prac. 2004;46(9):5-8.

(56) E. Whyle. An organisational typology of public-private engagement for health in Southern Africa: A systematic review. University of Cape Town; 2015.

(57) Cruz CO, Marques RC. Integrating infrastructure and clinical management in PPPs for health care. J Manage Eng. 2013;29(4):471-481.

(58) Whyle EB, Olivier J. Models of public–private engagement for health services delivery and financing in Southern Africa: a systematic review. Health Policy Plan. 2016;31(10):1515-1529.

(59) Grieve A, Olivier J. Towards universal health coverage: a mixed-method study mapping the development of the faith-based non-profit sector in the Ghanaian health system. Int J Equity Health. 2018;17(1):1-20.

(60) Olivier J, Smith S. Innovative faith-community responses to HIV and AIDS: Summative lessons from over two decades of work. Rev Faith Int Aff. 2016;14(3):5-21.

(61) Boulenger D, Barten F, Criel B. Contracting between faith-based health care organizations and the public sector in Africa. Rev Faith Int Aff. 2014;12(1):21-29.

(62) Olivier J, Tsimpo C, Gemignani R, Shojo M, Coulombe H, Dimmock F, et al. Understanding the roles of faith-based health-care providers in Africa: review of the evidence with a focus on magnitude, reach, cost, and satisfaction. Lancet. 2015;386(10005):1765-1775.

(63) Dimmock F, Olivier J, Wodon Q. Network development for non-state health providers: African Christian health associations. Dev Pract. 2017;27(5):580-598.

(64) Olivier J, Shojo M, Wodon Q. Faith-inspired health care provision in Ghana: market share, reach to the poor, and performance. Rev Faith Int Aff. 2014;12(1):84-96.

(65) Olivier J. Hoist by our own petard: Backing slowly out of religion and development advocacy. HTS Theological Studies. 2016;72(4):1-11.

(66) Bitran RA. Private health sector assessment in Ghana. Washington, DC: World Bank Publications; 2011.

(67) Dimmock F, Olivier J, Wodon Q. Half a century young: the Christian Health Associations in Africa. The Role of Faith-Inspired Health Care Providers in Sub-Saharan Africa and Public-Private Partnerships. 2012:71.

(68) Seniwoliba JA. The Single Spine Pay Policy: Can Ignorance Derail the Benefits it Has on the Ghanaian Public Service Worker? ESJ. 2014;10(8).

(69) Seniwoliba JA. Ghana's numerous workers strikes; a cause for concern. MRJER. 2013 SEP,;1(8):161-171.

(70) Yeboah P, Buckle G. The evolving partnership between the Government of Ghana and national faith-based health providers: leadership perspective and experiences from the Christian Health Association of Ghana. Dev Pract. 2017;27(5):766-774.

(71) P. Yeboah. Harmonization challenges of recent health reforms for the Christian health association of Ghana (CHAG)Royal tropical institute (KIT). 2008.

(72) MOH. National health policy: Ensuring healthy lives for all. Ministry of Health. 2020 Jan.

(73) Asamani JA, Ismaila H, Plange A, Ekey VF, Ahmed A, Chebere M, et al. The cost of health workforce gaps and inequitable distribution in the Ghana health service: an analysis towards evidence-based health workforce planning and management. Hum Resour Health. 2021;19(1):1-15.

(74) Ministry of Health. Ghana's roadmap for attaining Unviersal Health Coverage 2020-2030. Ministry of Health Ghana. 2020 Jan,:1-8.

(75) Ministry of Health. Holistic assessment of the health sector programme of work 2014. Ministry of Health. 2018 Jul,:6-19.

(76) MOH. Public health sector development plan . Ministry of Health. 2012 Apr.

(77) Boateng J, Surnye H, Mensah A, Boateng B, Nyarko P, Munguti N, et al. Costs of reproductive health services provided by four Christian Health Association of Ghana (CHAG) hospitals. Population Council 2006:3-8.

(78) Olivier J, Kwamie A. The history of public- (faith-based) private health sector partnership in Ghana. World Health Organization. 2017.

(79) Agyepong IA, Kodua A, Adjei S, Adam T. When 'solutions of yesterday become problems of today': crisis-ridden decision making in a complex adaptive system (CAS)—the Additional Duty Hours Allowance in Ghana. Health Policy Plan. 2012;27(suppl_4):iv20-iv31.

(80) Akwasi Sarpong. Doctors in Ghana continue to strike over salary dispute. 2013; Available at: https://www.bbc.com/news/av/world-africa-22128960. Accessed Aug 7, 2022.

(81) Awori SN, Tettey-Enyo A. An evaluation of the effects of industrial unrest at the Korle-Bu Teaching Hospital (KBTH), Accra, Ghana. ISJ 2015;7(2):424-428.

(82) The Finder. Doctors' strike: 500 dead in 17 days. 2015; Available at: https://www.ghanaweb.com/GhanaHomePage/NewsArchive/Doctors-strike-500-dead-in-17-days-375648. Accessed Aug 7, 2022.

(83) Daily Guide. Pain & anguish as Doctors dump hospitals. 2015; Available at: https://www.ghanaweb.com/GhanaHomePage/NewsArchive/Pain-anguish-as-Doctors-dump-hospitals-373844. Accessed Aug 7, 2022.

(84) Asamani JA, Amertil NP, Ismaila H, Akugri FA, Nabyonga-Orem J. The imperative of evidence-based health workforce planning and implementation: lessons from nurses and midwives unemployment crisis in Ghana. Hum Resour Health 2020;18(1):1-6.

(85) Joy News. Press statement delivered by the Minister of Health, Hon. Alex Segbefia, on the ongoing illegal strike action by the GMA on 18th August. 2015; Available at: https://pt-br.facebook.com/JoyNewsOnTV/posts/press-statement-delivered-by-the-minister-for-health-hon-alex-segbefia-on-the-on/943787905693116/. Accessed Aug 5, 2022.

(86) The Finder. Negotiations: No invite from govt to GMA. 2015; Available at: https://www.ghanaweb.com/GhanaHomePage/NewsArchive/Negotiations-No-invite-from-govt-to-GMA-375651. Accessed Aug 7, 2022.

(87) KasapaFm. Catholic Church adopting slave labour in Ghana – ex-Minister. 2015; Available at: https://www.ghanaweb.com/GhanaHomePage/NewsArchive/Catholic-Church-adopting-slave-labour-in-Ghana-ex-Minister-374511. Accessed Aug 7, 2022.

(88) Tv3 Network. No pressure on us – 37 Military Hospital. 2015; Available at: https://www.ghanaweb.com/GhanaHomePage/NewsArchive/No-pressure-on-us-37-Military-Hospital-373577. Accessed Aug 7, 2022.

(89) A. Grieve. Towards Universal Health Coverage: Mapping the Development of the Faith-Based Non-Profit Sector in the Ghanaian Health System. University of Cape Town; 2017.

(90) Robson C. Real world research: A resource for social scientists and practitioner-researchers. 2nd ed.: Wiley-Blackwell; 2002.

(91) Yin RK. Case study research: Design and methods. 3rd ed. United Kingdom, London: Sage; 2009.

(92) Yin RK. Case study research : design and methods. 5th ed. United Kingdom, London: Sage; 2014.

(93) Gustafsson J. Single case studies vs. multiple case studies: A comparative study. 2017.

(94) Villamayor-Tomas S, Oberlack C, Epstein G, Partelow S, Roggero M, Kellner E, et al. Using case study data to understand SES interactions: a model-centered meta-analysis of SES framework applications. Curr Opin Environ Sustain 2020;44:48-57.

(95) Gilson L, Hanson K, Sheikh K, Agyepong IA, Ssengooba F, Bennett S. Building the field of health policy and systems research: social science matters. PLoS Med 2011;8(8):e1001079.

(96) Gilson L, Raphaely N. The terrain of health policy analysis in low and middle income countries: a review of published literature 1994–2007. Health Policy Plan. 2008;23(5):294-307.

(97) Bowen GA. Document analysis as a qualitative research method. Qual Res J. 2009;9(2):27-40.

(98) Gibson W, Brown A. Working with qualitative data. Sage Publications. 2009.

(99) Clarke V, Braun V. Using thematic analysis in counselling and psychotherapy research: A critical reflection. Couns Psychother Res 2018;18(2):107-110.

(100) Schiller CJ. Critical realism in nursing: an emerging approach. Nurs Philos. 2016;17(2):88-102.

(101) Gilson L, World Health Organization. Health policy and systems research: a methodology reader. World Health Organization. 2012.

(102) General Assembly of the World Medical Association. World Medical Association Declaration of Helsinki: ethical principles for medical research involving human subjects. J Am Coll Dent. 2014;81(3):14-18.

(103) Amy Whiting. Mapping the multiple intersectoral spaces for civil society participation and responsiveness strengthening in the South African health system–focusing on the Western Cape. University of Cape Town; 2021.

(104) Olivier J, Scott V, Molosiwa D, Gilson L. Systems approaches in health systems research: approaches for embedding research. In: Savigny D, Blanchet K, Adam T, editors. Applied systems thinking for health systems research: a methodological handbook. New York: McGraw-Hill Education; 2017. p. 10-19.

(105) Aragón AO. A case for surfacing theories of change for purposeful organisational capacity development. IDS Bulletin. 2010;41(3):36-46.

Appendices

Appendix 1: Table of relevant results for scoping review on health worker strikes in Ghana

Source	Source Type	Title	Focus and/or methodology	Relevance
Awori and Tettey-Eno	Journal article	An evaluation of the effects of industrial unrest at the Korle-bu Teaching Hospital (KBTH), Accra, Ghana	Assessed the impact of industrial unrest from the perspective of a cross-section of 50 patients and nurses at KBTH from the period 2000-2008. Statistical data was gathered from randomly selected study subjects	Explored the views of nurses regarding the reasons behind the health worker strikes that occurred from 2004 to 2008 and the effects of these strikes on the healthcare system. The nurses identified unequal pay between themselves and other health workers as the main reason for the strikes. Most of the nurses agreed that the strikes had a negative impact on their patients. Additionally, they felt that the government did little to effectively address the strikes. The study suggested that the government should work towards reducing the wage gap in Ghana and establish crisis management committees at hospitals during strikes to prevent further harm to patients.
Agyepong et al (2012)	Journal article	When 'solutions' of yesterday become problems of today': crisis-ridden decision-making in a complex adaptive system (CAS)- the Additional Duty Hours Allowance in Ghana	The paper explores the implication of the ADHA policy on increasing health worker strikes in the early 2000s. The study implored a case study methodology of decision-making processes around the Additional Duty Hours Allowance Policy in Ghana through the analysis of secondary data and selected key-informant interviews.	The paper examines the factors behind the health worker strikes in Ghana in the early 2000s, primarily in reaction to the ADHA policy. The strikes are portrayed as highly political and complex events that are influenced by various players. The paper emphasizes the necessity of strike resolution policies and strategies to comprehend the complexities of adaptive systems and the influence of interest, power, and interactions among different stakeholders.
Seniwoliba 2013	Journal article	Ghana's numerous worker strikes; a cause of concern	The study reports on stories and issues on labor unrest in Ghana and factors that cause labour unrest in Ghana in all sectors, including the health sector. Data was collected from internet sites such as ghanaweb, myjoyonline, citinews, and Google.	The paper delves into the broader context of labor unrest in Ghana that contributes to health worker strikes. This includes both economic factors such as compensation, wages, bonuses, and allowances, and non-economic causes such as the failure to implement policies, discrimination, and segregation. The paper also examines the Labor Act of 2003 and its impact on the health worker strikes in Ghana. In conclusion, the paper suggests that the labor laws in Ghana should be re-evaluated to give the National Labor Commission the power to impose penalties on individuals who ignore Alternative Dispute Resolution provisions. This would lead to more effective practices and resolve disputes more efficiently.

Asamani et al (2020)	Journal article	The imperative of evidence-based health workforce planning in implementation: lessons from nurses and midwives unemployment crisis in Ghana	The paper explores policy decisions made by the Government of Ghana to expand and liberalise the training of health workers in Ghana and the policy lapses that resulted in unintended paradoxical unemployment in the nation.	The paper explains the causes of strikes amongst unemployed trained nurses and midwives. The liberalisation of training of health workers allowed the profit-driven private sector to train nurses and midwives. This led to a large number of applicants beyond what the health system could absorb and resulted in strikes by unemployed nurses and midwives for health sector jobs.
Seniwoliba 2014	Journal article	Single Spine Pay Policy, Can Ignorance delay the benefits it has on the Ghanaian public service worker	The Paper explores the history of the Single Spine Salary Structure in Ghana and the tensions it created for public service workers.	The Paper explains how the Single Spine Salary Structure contributed to increasing strikes in public sectors in Ghana, including healthcare workers.
Daily Guide (2015, August 2020)	Media report (GNA)	Doctors defy Gov't order	2015 doctor strike	Explains the context of the 2015 doctor strikes in Ghana. Relevance to resilience for strikes- discussion of labour laws in place to allow for resolution of strikes.
The Finder (2015, August 17)	Media report (GNA)	Doctors' strike: 500 dead in 17 days	2015 doctor strike	The paper examines the impact of the 2015 doctor strikes in Ghana. According to the Health Insurance Service Provider Association, 500 people lost their lives during the nationwide strike. The report suggests that these deaths were likely a result of the strike. The paper also explores the economic situation in Ghana at the time that could have potentially fuelled the strike.
Reuters (2015, August 14)	Media Report (GNA)	Doctors likely to call off strike, union says	2015 doctor strike	Reports on some of the reasons behind the 2015 doctor strike in Ghana. The Ghana Medical Association (GMA) highlights that the strikes were not solely about payment but rather about the government's failure to provide a clear and defined set of working conditions for doctors.
KasapaFM (2015, August 12)	Media Report (GNA)	Withdrawal of doctors' salary on point – Mahama	2015 doctor strike	The Progressive People's Party (PPP) advises government to engage with striking doctors in the 2015 doctor strike in a respectful manner and desist from political polys and insults. Comments that the government should not resolve labour issues by using party communicators in the media. Accused both the NDC and the NPP of using the strikes as a political opportunity. Accuses the government of leaking negotiation documents to the media and urged the government to negotiate in good faith.
Daily Guide (2015, August 8)	Media Report (GNA)	Pain & anguish as Doctors dump hospitals	2015 doctor strike	Explores conditions during the 2015 doctor strike. Doctors withdrew emergency and OPD services leaving many to have to rely on services in the private sector.

Ghana Web (2015, August 8)	Media Report (GNA)	Doctors' Strike: Out-Patient Departments empty (Photos)	2015 doctor strike	The report provides information on the situation in public and private hospitals during the 2015 doctor strikes in Ghana. As a result of the Ghana Medical Association's (GMA) decision to withdraw emergency and OPD services during the strike, patients were redirected to private hospitals and quasi-government institutions. The report mentions that the increased number of patients in private hospitals created difficult and challenging conditions due to the overflow of patients.
Tv3 Network (2015, August 7)	Media Report (GNA)	No pressure on us – 37 Military Hospital	2015 doctor strike	The report focuses on the conditions in private hospitals during the 2015 doctor strikes in Ghana. To deal with the increased patient load during the strike, a private hospital implemented various strategies, including recalling staff members who were on leave and setting up tents to provide additional space for patients. These measures were taken to ensure that the hospital could continue to provide services to patients despite the strain on the healthcare system caused by the strikes.
Ghanaian Times (2015, August 7)	Media Report (GNA)	GHS boss appeals to doctors to end strike	2015 doctor strike	Describes the strategies used by the government during the 2015 doctor strike in Ghana to ensure the provision of healthcare services. The Ghana Health Service (GHS) collaborated with private sector doctors by granting them access to public health facilities and encouraging them to take on a greater share of the public health needs in the capital. This was done to address the shortage of healthcare providers caused by the strike and ensure that patients continued to receive the necessary medical care.
KasapaFM (2015, August 11)	Media Report (GNA)	Catholic Church adopting slave labour in Ghana – ex-Minister	2015 doctor strike	National Catholic Health Service dismisses 14 employees for joining the national doctor strike, in doing so, affirming their non-striking position.
Sarapong (2013)	Media Report (GNA)	Doctors in Ghana continue to strike over a salary dispute	2013 Nurse strike	Reports on the 2013 national doctor strike in the public sector in Ghana. During the strike, doctors suspended outpatient and emergency services, but continued to provide in-patient services. The strike was a result of a long-standing salary dispute between the Ghana Medical Association (GMA), the Ministry of Health (MOH), and the Social Security and National Insurance Trust (SSNIT). Patients were directed to seek medical assistance from private institutions and the government stated that it would support these institutions in handling the increased patient load. The report provides information on the events of the 2013 national doctor strike and its impact on the healthcare system.

Olivier and Kwamie (2017)	Journal paper	The history of public- (faith-based) private health sector partnership in Ghana.	historically-focused mixed-methods study using geospatial mapping to explore the historical role of CHAG in Ghana towards UHC	Discusses the secondment strategy between the Christian Health Association of Ghana (CHAG) and the Ministry of Health (MOH) has changed the way CHAG staff are compensated and managed. Currently, the majority of CHAG staff receive their salaries from the MOH but are managed and administered by CHAG, and they receive the same conditions of service and benefits package as the Ghana Health Service (GHS) staff. Additionally, CHAG has a strict policy against strikes, and during health worker strikes, patients are redirected to CHAG facilities by the government.
Yeboah and Buckle (2017)	Journal paper	The evolving partnership between the Government of Ghana and national faith-based health providers: leadership perspective and experiences from the Christian Health Association of Ghana	Narrative of the evolving relationship between CHAG and the MOH	CHAG holds a strict non-striking stance and this allows for continuous delivery of health services even during strikes by health workers in Ghana, thereby reducing the negative impact on vulnerable populations. 2) Issues with delayed payments from the NHIA to CHAG and MOH have added tension to their relationship. 3) CHAG played a critical role during the indefinite strike by government pharmacists in September 2016, as the MOH publicly directed patients to receive services at CHAG facilities.
Negotiations: No invite from govt to GMA	Government Briefing	Negotiations: No invite from govt to GMA	2015 doctor strike	During the 2015 doctor strikes, the government instructed the public to seek care from alternative healthcare facilities that were still in operation. The government also released a list of these facilities and emphasized that NHIS card holders were eligible for treatment at a large number of private and faith-based healthcare facilities as well as community-based health planning services across the country. This was an effort to ensure that the healthcare needs of the population were still being met despite the strikes.
**Duho (2016, September 6)**	Media Report (GNA)	Pharmacists in govt hospitals begin indefinite strike	2016 Pharmacist strike	In September 2016, pharmacists in the public sector in Ghana embarked on an indefinite strike for improved salaries and conditions of service. During the strike, the public was directed to receive pharmaceutical services from private providers and facilities.
MOH (2015, June 26)	MOH Draft Document	Draft Conditions of service for medical doctors and dentists	The draft conditions of service that were negotiated between the MOH and the GMA during the 2015 strikes	The context for 2015 strikes and the nature of the HRH crisis persistent in Ghana, and the history of this crisis.
Joy News (2015, August 18)	Press statement	Press statement delivered by the Minister of Health, Hon. Alex Sebgefia on the ongoing illegal strike action by the GMA on 18th August	2015 doctor strike	A public press statement by the Minister of Health during the 2015 doctor strikes directed patients seeking care to CHAG facilities and other private facilities. Minister of Health notes steps taken by the government to respond to strikes, including providing resources to private facilities and re-enlisting retired doctors. Minister also publishes a public list of facilities still in operation during the strike.

Appendix 5: Russo et al (2019) Framework

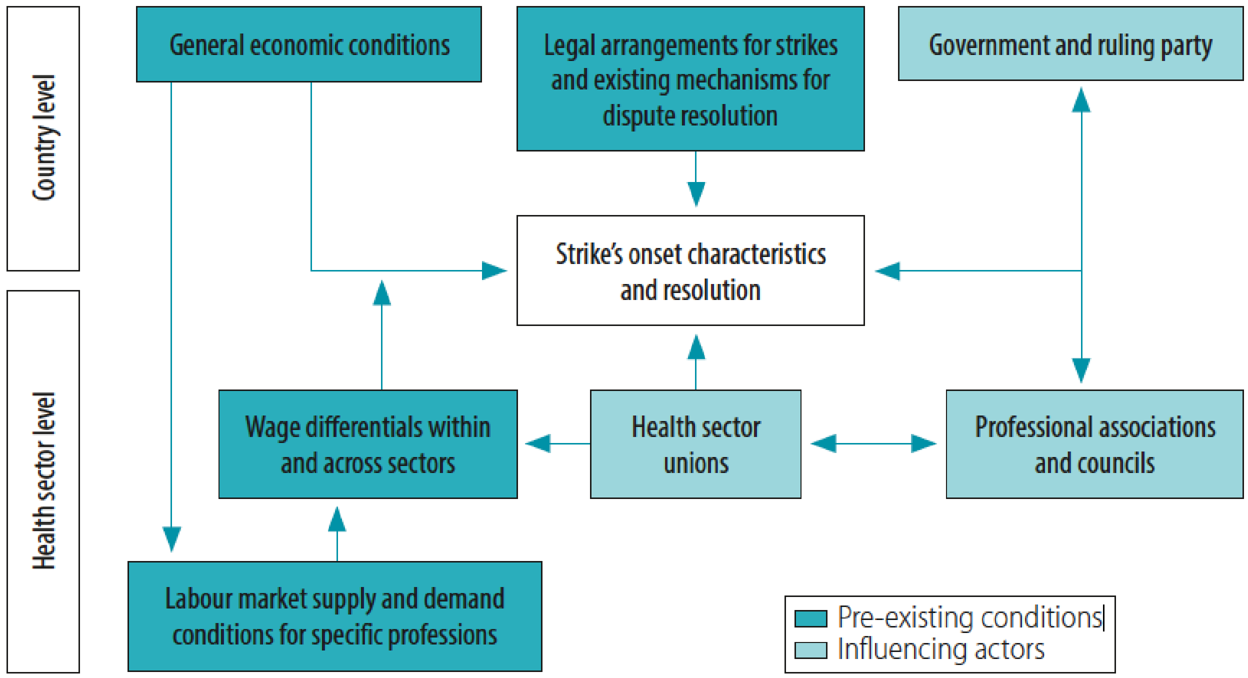

Appendix 6: Kagwanja et al (2020) framework

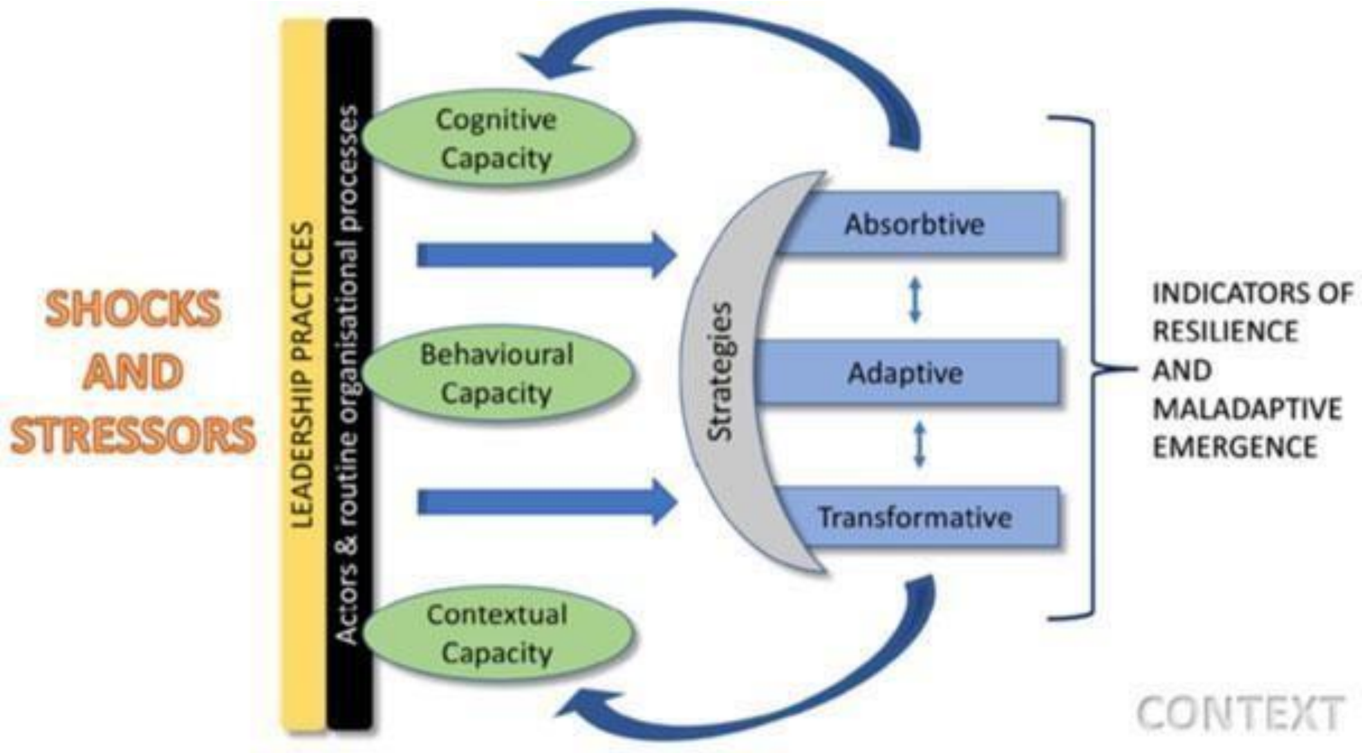

Appendix 7 Theoretical Frameworks for data collection and analysis

Convergence of theoretical frameworks for data collection and analysis

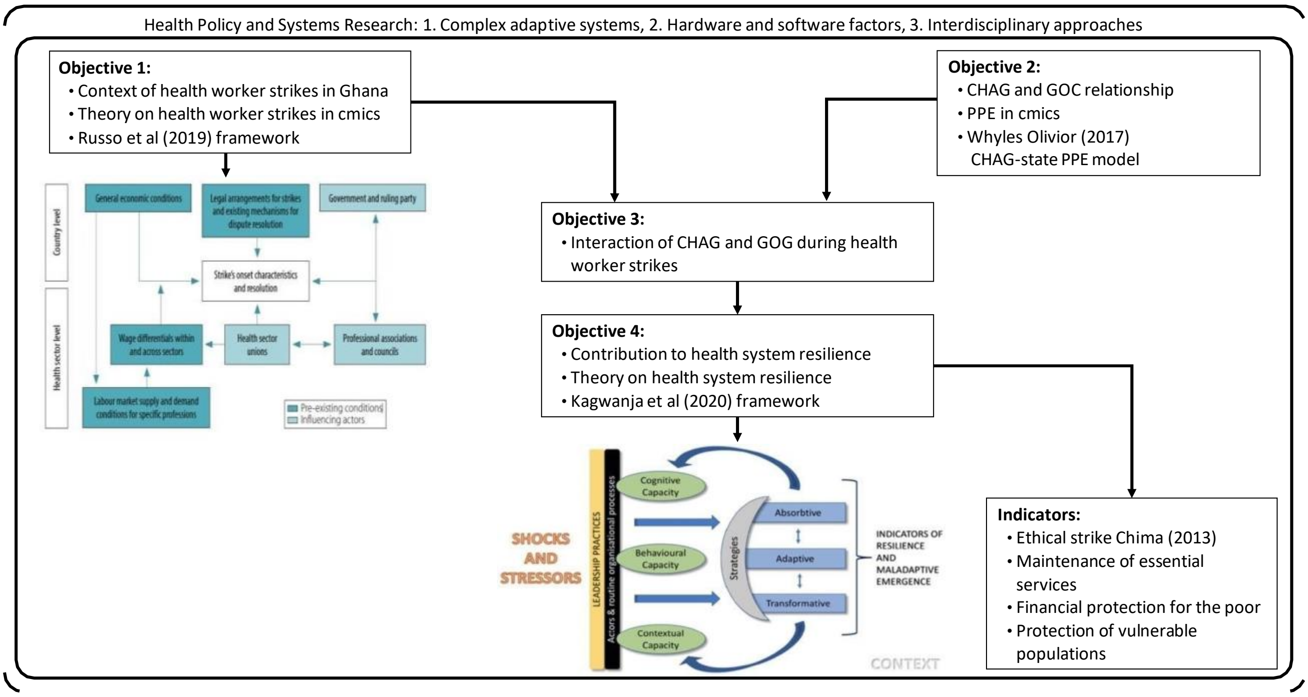

Appendix 11 CHA-State PPE Model (Source Whyle and Olivier (2017))

Table 2. The common CHA–State PPE found in SSA – lying across PPP and contracting out models.

	PPP	Contracting-out
Management and decision-making	Shared-decision making	Transfer of decision-making responsibiliti
Degree of collaboration	Close collaboration, large degree of negotiation and trust	Hierarchical relationship – public sector "buys" services
Knowledge transfer	High levels of knowledge transfer	No knowledge transfer due to low levels collaboration
Risk-sharing	Always has a risk-sharing component	Can involve risk-sharing but does not necessarily do so.
Resource sharing	Sharing of financial, infrastructural, and/or human resources	Limited resource sharing aside from trans of funds
Payment mechanisms	Always includes some form of performance-based payment	Private partner paid directly by public authority, not necessarily performance-based
Payment schedule	Private party bears all initial costs and is paid in instalments, moving payments "off-budget"	Various: no shifting of costs "off-budget"
Service-mix/bundling	Mixed/bundled services contracted out to a single private partner, streamlining incentives and increasing efficiency	No bundling, each contract involves the provision of a single set of services
Length of contract	Long-term contract	Generally shorter-term contract
